# A Systematic Literature Review of the Effectiveness of CDK 4/6 Inhibitors for First-Line Treatment of HR+/HER2− Advanced/Metastatic Breast Cancer: A Comparison of Real-World Evidence

**DOI:** 10.3390/cancers18142362

**Published:** 2026-07-22

**Authors:** Timothy Pluard, Thomas Grinda, Rodrigo Dienstmann, Marc Thill, Beata Korytowsky, Connie Chen, Sofiya Portuhay, Elizabeth M. Salvo-Halloran, Imtiaz A. Samjoo, Nadia Harbeck

**Affiliations:** 1 Hematology and Medical Oncology, St. Luke’s Cancer Institute, Kansas City, MO 64111, USA; tpluard@saint-lukes.org; 2 Department of Cancer Medicine, Gustave Roussy Institute, 94800 Villejuif, France; thomas.grinda@gustaveroussy.fr; 3 Vall d’Hebron Institute of Oncology (VHIO), Vall d’Hebron Barcelona Hospital Campus, 08035 Barcelona, Spain; rdienstmann@vhio.net; 4 Department of Gynecology and Gynecologic Oncology, Agaplesion Markus Hospital, 60431 Frankfurt am Main, Germany; marc.thill@agaplesion.de; 5 Pfizer, Inc., New York, NY 10001, USA; beata.korytowsky@pfizer.com (B.K.); connie.chen@pfizer.com (C.C.); 6 Value & Evidence, EVERSANA^TM^, Burlington, ON L7N 3H8, Canada; sofiya.portuhay@eversana.com (S.P.); elizabeth.halloran@eversana.com (E.M.S.-H.); 7 Breast Center, Department of Gynecology and Obstetrics and Comprehensive Cancer Center (CCC Munich LMU), LMU University Hospital, 81377 Munich, Germany; nadia.harbeck@med.uni-muenchen.de

**Keywords:** CDK4/6 inhibitors, palbociclib, ribociclib, abemaciclib, real-world evidence, metastatic breast cancer, real world progression-free survival, overall survival, comparative effectiveness

## Abstract

**Background/Objective:** Cyclin-dependent kinase 4/6 inhibitors (CDK4/6i) combined with endocrine therapy are the standard first-line treatment for HR+/HER2− advanced/metastatic breast cancer (a/mBC), yet no head-to-head randomized trials have compared palbociclib, ribociclib, and abemaciclib. This systematic literature review (SLR) aimed to synthesize available real-world evidence (RWE) on the comparative effectiveness of these three CDK4/6i in the first-line setting. **Methods:** An SLR was conducted following PRISMA guidelines. Searches of MEDLINE, Embase, Cochrane, and gray literature (January 2015–September 2025) identified RWE studies reporting real-world progression-free survival (rwPFS) and/or overall survival (OS) for first-line CDK4/6i regimens. Eligible studies included adults with HR+/HER2− a/mBC receiving palbociclib, ribociclib, or abemaciclib, combined with endocrine-based therapy. Comparative outcomes were summarized qualitatively; study quality was assessed using the Newcastle–Ottawa Scale, ISPOR-AMCP-NPC questionnaire, and ESMO-GROW checklist. **Results:** From 13,345 records, 39 publications (32 unique studies) were identified, of which 21 were full-text studies meeting inclusion criteria. Most included studies used retrospective cohort designs, and approximately 50% evaluated all three CDK4/6is. Given the comparative nature of the included studies, quality issues were found to be related to study design and analytical approaches. Several analyses were unadjusted, and reporting of follow-up duration varied across studies, with some analyses providing incomplete follow-up information. Study sample sizes differed substantially across agents, with smaller populations generally reported for ribociclib and abemaciclib. Among the studies reporting rwPFS hazard ratios (HRs), 8/11 studies showed comparable rwPFS and 5/6 studies reported comparable OS outcomes between CDK4/6i regimens. Three full-text studies reported HRs favoring ribociclib or abemaciclib relative to palbociclib; these analyses primarily included populations receiving palbociclib, with smaller comparator cohorts for ribociclib and abemaciclib, as well as varied follow-up times. **Conclusions:** Most real-world studies included in the SLR suggested similar effectiveness of the three CDK4/6is in first-line HR+/HER2− a/mBC. Heterogeneity in patient characteristics, definitions of outcomes, follow-up time, sample size, and statistical approach may have important implications on study interpretability.

## Simple Summary

Drugs known as cyclin-dependent kinase 4/6 inhibitors are commonly used together with hormone therapy as the first treatment for people with hormone receptor-positive, HER2-negative advanced breast cancer. However, these drugs have not been directly compared in large clinical trials. This research reviewed published studies that used real-world patient data to compare how well the three available drugs, palbociclib, ribociclib, and abemaciclib, work in routine clinical practice. Most studies showed that patients had similar outcomes regardless of which drug was used, including similar time before the disease worsened and similar overall survival. Differences between studies, such as how long patients were followed and how results were analyzed, made some findings difficult to compare. Overall, this review suggests that all three treatments may have broadly similar effectiveness, which can help researchers and clinicians better understand treatment choices in everyday care.

## 1. Introduction

Breast cancer is the most commonly diagnosed cancer worldwide and the leading cause of cancer-related death among women. According to the World Health Organization’s International Agency for Research on Cancer, approximately 1 in 20 women globally will be diagnosed with breast cancer in their lifetime. If current trends continue, the global burden is projected to rise to 3.2 million new cases and 1.1 million deaths annually by 2050 [1]. Among breast cancer subtypes, hormone receptor-positive/human epidermal growth factor receptor 2-negative (HR+/HER2−) advanced or metastatic breast cancer (a/mBC) is the most prevalent, representing 70% of cases according to Surveillance, Epidemiology, and End Results data from 2018 to 2022 [2].

In the advanced or metastatic setting, HR+/HER2− disease remains a major contributor to cancer-related morbidity and mortality due to its chronic nature and potential for resistance to endocrine therapy (ET). The introduction of cyclin-dependent kinase 4/6 inhibitors (CDK4/6i), palbociclib, ribociclib, and abemaciclib, has significantly improved clinical outcomes when used in combination with ET, establishing these agents as the preferred standard of care in the first-line advanced setting [3,4,5,6,7].

Despite their foundational role in establishing efficacy, randomized controlled trials (RCTs) are inherently limited in their ability to reflect the complexities of real-world clinical practice and treated populations. Strict eligibility criteria, protocol-defined treatment regimens and procedures, and short follow-up durations often result in study populations that are not representative of the broader patient community. For example, recent data suggest that up to 40% of patients in cancer registries may be ineligible for participation in RCTs [8]. Moreover, RCTs typically exclude patients with poor performance status, comorbidities, or those from underrepresented demographic groups, such as elderly individuals or racial minorities, thereby limiting the generalizability of their findings [9].

In contrast, real-world evidence (RWE) derived from observational studies, electronic health records, registries, and claims data offers a complementary and often more inclusive perspective on treatment effectiveness and safety. RWE captures outcomes in diverse patient populations over extended periods, providing insights into treatment patterns, adherence, and long-term effects that are not feasible to assess in RCTs.

Given the growing body of comparative effectiveness between CDK4/6i’s in a/mBC, a systematic literature review (SLR) of these data is warranted to synthesize the evolving evidence landscape [10,11]. This approach is particularly valuable in oncology, where treatment decisions must often be made in the absence of head-to-head RCTs [8].

Here, we present a systematic review of real-world studies evaluating the effectiveness of palbociclib, ribociclib, or abemaciclib + ET regimens in the first-line treatment of HR+/HER2− a/mBC.

## 2. Materials and Methods

### 2.1. Literature Search

This SLR was conducted and reported in accordance with the Preferred Reporting Items for Systematic Reviews and Meta-Analyses 2020 guidelines (Table S1) [12]. A formal review protocol was developed a priori, but was not registered. With respect to protocol deviations, we confirm that no modifications were made that reduced the methodological rigor of the review relative to the original internal protocol. The sole substantive evolution of the protocol since the initial 2019 search iteration involved the introduction of a minimum sample size threshold as an additional eligibility criterion and the incorporation of the European Society for Medical Oncology Guidance for Reporting Oncology Real-World Evidence (ESMO-GROW) checklist as a supplementary quality assessment instrument—both of which represent enhancements to rigor rather than relaxations of standards. No eligibility criteria were removed, no outcomes were added post hoc, and no analytical decisions were made in a manner that could introduce outcome-reporting or post hoc selection bias. The database search was initially performed in July 2019 [10] with the most recent update conducted on 11 September 2025. Databases included MEDLINE, Embase, and Cochrane Library. The most recent search strategy is detailed in Supplementary Information S1.

In addition to database searches, gray literature searches were conducted across prespecified key clinical conferences to capture relevant publications from January 2015 to September 2025. Conferences searched included the San Antonio Breast Cancer Symposium, the American Society of Clinical Oncology, ESMO, ESMO Breast Cancer, ESMO Asia, the Professional Society for Health Economics and Outcomes Research (ISPOR), and ISPOR Europe. To validate the findings from the database and gray literature searches, supplementary searches were conducted using ClinicalTrials.gov and the reference lists of relevant published reviews.

### 2.2. Study Selection and Data Extraction

Two independent reviewers screened studies for eligibility using the predefined Population, Intervention, Comparator, Outcome, and Study design (PICOS) criteria outlined in Table 1. Any discrepancies arising during the screening process were resolved through consensus between the two reviewers, with a third reviewer consulted to adjudicate unresolved disputes. Since the original search, conducted in 2019, some additional restrictions were placed on the eligibility criteria, including a minimum sample size.

Data from included publications were extracted into a standardized spreadsheet by one reviewer and independently verified for accuracy and completeness by a second reviewer. Any issues were addressed through reviewer discussion. For records with a high volume of subgroup data, subgroups outlined in Table S3 were prioritized for extraction.

Eligible studies reported RWE for adult patients (aged ≥ 18 years) diagnosed with HR+/HER2− a/mBC who received CDK4/6i in combination with ET—specifically palbociclib, ribociclib, or abemaciclib—as first-line treatment. Inclusion required comparative data for at least two CDK4/6i arms. Three-way comparative analyses, while captured where available, were not mandated. Outcomes of interest included median real-world progression-free survival (rwPFS) and/or median overall survival (OS), and studies were eligible if either comparative data for the regimens (e.g., hazard ratios [HRs]) or regimen-specific data (e.g., median, rates) were reported. To strengthen the reliability of the review findings and ensure the relevance of the summarized literature, studies were excluded if they enrolled fewer than 100 patients in total, did not specify the line of therapy, or did not identify the specific CDK4/6i agent administered. Additionally, only studies in which at least one treatment arm included >50 patients were included. Studies were also excluded if published in any language other than English, or before 2015 (year when the first CDK4/6i was approved).

### 2.3. Data Analysis

This SLR was conducted as a qualitative evidence synthesis; a formal quantitative meta-analysis was beyond the scope of the current review, though the evidence base compiled herein may serve as a foundation for future meta-analytic investigation.

Efficacy outcomes of interest (i.e., rwPFS and/or OS) were summarized by CDK4/6i comparator pairs (i.e., palbociclib versus ribociclib, palbociclib versus abemaciclib, or ribociclib versus abemaciclib) and by patient population (i.e., overall population or specific subgroups). Subgroup-level findings are reported in Tables S5, S7, S9, S11, S13, S15, and Supplementary Information S2. It should be noted that the number of studies contributing to any given subgroup analysis may not equal the total number of included studies, as individual studies may report on more than one subgroup. The ascertainment of rwPFS is inherently complex in observational settings, as it relies on routine clinical documentation rather than protocol-defined assessment schedules, and definitions vary considerably across data sources, health systems, and study teams. As such, we have cataloged all available rwPFS definitions reported across the included studies, presented in Table S16. The qualitative synthesis prioritized studies reporting HR values for comparisons between distinct treatment regimens, with median values also captured where available. Findings were stratified by publication type, with results from full-text studies presented separately and prioritized over those from abstract-only studies.

### 2.4. Quality Assessment

Quality assessment was restricted to full-text publications, as conference abstracts did not provide sufficient methodological detail to permit a meaningful evaluation. One independent reviewer performed the study quality assessments, which were validated by a second independent reviewer. Discrepancies were resolved through consensus or a third reviewer. Risk of bias assessment was performed for included studies using the Newcastle–Ottawa scale (NOS) for non-randomized studies (scores 7–9, 4–6, and <4 are considered low, intermediate, and high risk, respectively) [13]. For additional validation, the ISPOR–Academy of Managed Care Pharmacy (AMCP)–National Pharmaceutical Council (NPC) questionnaire [14] and ESMO-GROW checklist [15] were used to determine the risk of bias for the included comparative studies and to assess the quality of reporting and transparency.

## 3. Results

### 3.1. Literature Search and Study Selection

A total of 13,345 records were identified across all the searches conducted from July 2019 until the most recent search conducted on 11 September 2025 (10,378 from database searches and 2976 from gray literature searches). A summary of the literature search results and study selection process for each update is provided in Figure S1.

**Figure 1:**
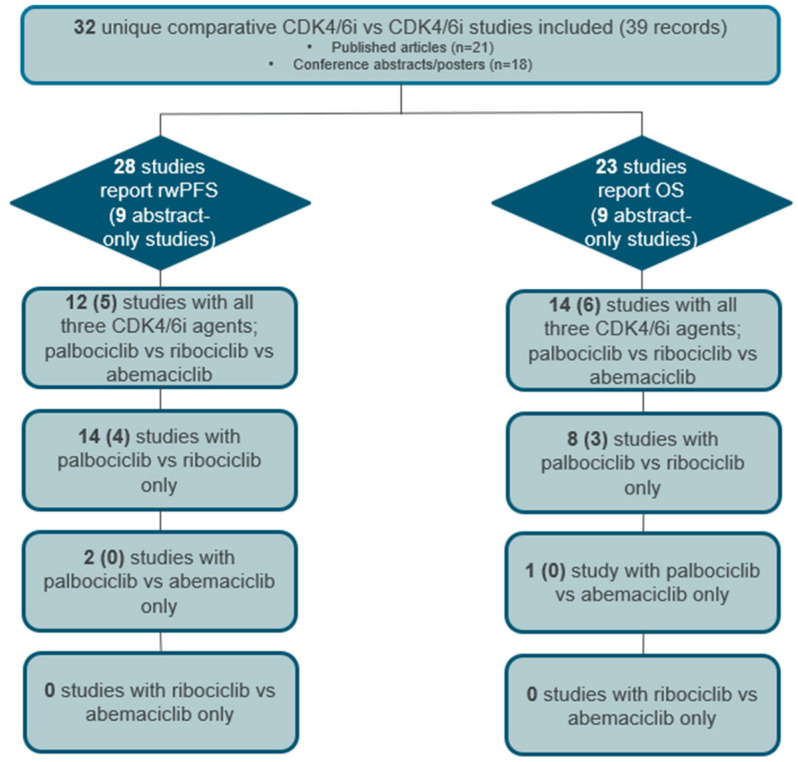
Study attrition diagram. Note: Parentheses are indicative of number of abstract-only studies. Abbreviations: CDK4/6i = cyclin-dependent kinase 4/6 inhibitors; OS = overall survival; rwPFS = real-world progression-free survival.

**Figure 2:**
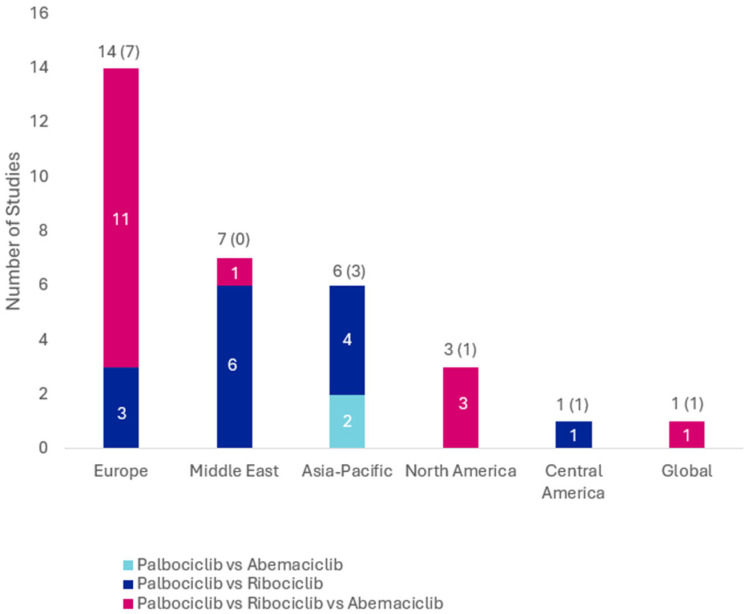
Regional distribution of included studies. Note: Parentheses are indicative of the number of abstract-only studies.

Notably, 155 records were excluded from this assessment for reasons such as small sample sizes (<100 patients), unspecified line of therapy or type of CDK4/6i assessed, data on CDK4/6i treatment beyond first-line therapy, and/or lack of reported outcomes of interest (i.e., rwPFS and/or OS). Consequently, 39 publications (21 full-text articles and 18 conference abstracts/posters) representing 32 unique studies reporting effectiveness data in the first-line setting were included in the qualitative synthesis [16,17,18,19,20,21,22,23,24,25,26,27,28,29,30,31,32,33,34,35,36,37,38,39,40,41,42,43,44,45,46,47,48,49,50,51,52,53,54]. Palbociclib was included universally in all studies. Among these, 16 studies (50.0%) compared palbociclib, ribociclib, and abemaciclib, 14 studies (43.8%) compared palbociclib with ribociclib, and 2 studies (6.3%) compared palbociclib with abemaciclib. For studies reporting rwPFS, 12 included all three agents, 14 included palbociclib and ribociclib only, and 2 included palbociclib and abemaciclib only. For studies reporting OS, 14 included all three agents, 8 included palbociclib and ribociclib only, and 1 included palbociclib and abemaciclib only (Figure 1). No studies exclusively compared ribociclib and abemaciclib. A list of included studies is shown in Table S2. Most RWE studies were retrospective cohort studies, representing both single-center and multicenter settings. Across all RWE study types, the majority (43.8%) were conducted in Europe, with the highest representation from Spain. Other regions included Asia Pacific, the Middle East, and North America. In addition, some studies were global in scope, enrolling patients across multiple regions (Figure 2).

Among the 21 included full-text articles, the NOS quality scores ranged from 4 to 9 points out of a possible 9 points, with 76.2% having a score of 7–9 (low risk of bias) and 23.8% having a score of 4–6 (intermediate risk of bias). The NOS evaluates key methodological domains, with the comparability domain being critical as it reflects whether studies controlled for confounding. Notably, NOS criteria for comparability are satisfied if any adjustment is reported, even if limited (e.g., multivariable analyses), rather than more vigorous methods such as stabilized inverse probability of treatment weighting (IPTW) or propensity score matching (PSM), underscoring the methodological challenges in generating credible comparative evidence from RWE.

To increase the rigor of quality assessment, the ISPOR-AMCP-NPC questionnaire and ESMO-GROW checklist were also utilized. From the perspective of reporting transparency, these tools evaluated the overall credibility of reporting quality as sufficient (100.0%; 21/21), meaning key elements required by these instruments, such as clarity of objectives, data sources, outcome definitions, and limitations, were present. However, these tools primarily evaluate reporting completeness and transparency and do not imply credibility of comparative evidence, as they do not imply methodological rigor of observational studies. Thus, transparent reporting being deemed sufficient for a clinical study based on multiple checklist questions should not be interpreted as high quality (Table 2).

Despite adequate reporting, risk of bias remained a major concern in the interpretation of observational studies as identified by the NOS and ESMO-GROW tools. Common issues from these tools included limited adjustment for baseline imbalances, immature OS follow-up, and reporting gaps, particularly regarding sample size justification, statistical power, missing data, and lost to follow-up. Full results for the conducted quality assessments are presented in File S1.

Beyond the structured quality assessment instruments, several study-level characteristics are essential for contextualizing the robustness of real-world comparative studies. Sample size and the distribution of patients across treatment groups directly influences the precision of effect estimates and the likelihood of imbalances that introduce bias. Follow-up duration, both overall and by treatment arm, further influences the ability to capture time-to-event outcomes, and shorter or unequal follow-up may bias rwPFS or OS comparisons. Similarly, the analytical strategy used to account for baseline heterogeneity plays a central role in mitigating confounding inherent to non-randomized data. While some (*N* = 7) studies employed methods such as IPTW or PSM to adjust for baseline demographic and clinical differences between treatment groups, many relied solely on multivariable analyses (*N* = 8) or reported unadjusted analyses (*N* = 6). Examples of adjustment factors included the Eastern Cooperative Oncology Group (ECOG) score, ET use (specifically fulvestrant), and distant or liver metastases. In some instances, small sample sizes may have precluded adjustment. Notably, some findings were only descriptive in nature. The non-randomized nature of these studies and lack of population adjustment (e.g., for prescribing biases) introduce substantial risk of confounding and raise important questions about whether these approaches are truly sufficient for comparative purposes. Table 3 provides additional context to complement formal quality assessment scores and aid interpretation of the results summarized in Table 2.

### 3.2. Effectiveness of CDK4/6i in Comparative Observational Studies

Of the 32 comparative studies (including direct and/or descriptive comparisons), rwPFS-only data was reported in 10, OS-only data was reported in 4, and data for both efficacy outcomes was reported in 18 studies. A total of 16 studies compared all three CDK4/6i treatments, with the remaining 16 comparing two of the three treatments; 14 compared palbociclib and ribociclib and 2 compared palbociclib and abemaciclib. The included studies were conducted across a wide geographic range, with the highest number from Turkey (*N* = 6), followed by Spain (*N* = 4), the United States (*N* = 3), and the United Kingdom (*N* = 3). Several studies also originated from Japan, Russia, Croatia, and multinational collaborations (*N* = 2), while others were single-country studies from Germany, China, Portugal, Poland, Denmark, Thailand, Costa Rica, and Italy.

#### 3.2.1. Baseline Characteristics in Studies Evaluating All Three CDK4/6i of Interest

Across the 16 studies (22 records; 8 full-text and 8 abstract-only) comparing all three CDK4/6i treatments, baseline characteristics by treatment group were available in 9 studies (6 full-text and 3 abstract-only studies). Imbalances in baseline characteristics were observed across studies, with only four full-text studies applying statistical methods to enable balanced comparisons between treatment arms and mitigate bias.

Palbociclib comprised the largest number of patients (*n* = 11,273) across all included studies, compared to ribociclib (*n* = 4828) and abemaciclib (*n* = 2602). Where reported, the length of follow-up in the palbociclib arms was 33.0–75.0 months, abemaciclib was 21.5–40.28 months, and ribociclib was 15.7–64.4 months, reflecting earlier approval and use.

Across studies which report treatment arm-specific baseline data, palbociclib recipients ranged from 39.8% to 80.6%. These patients were generally older (62 to 72 years), predominantly post-menopausal (53.8% to 91.7%), and more likely to have poorer ECOG performance status. In contrast, ribociclib was more frequently prescribed to younger patients (55 to 69.0 years). Abemaciclib cohorts were more likely to include patients with visceral disease (40.7% to 60.7%).

#### 3.2.2. Real-World Progression-Free Survival; Overall Population

##### Palbociclib vs. Ribociclib

**Figure 3:**
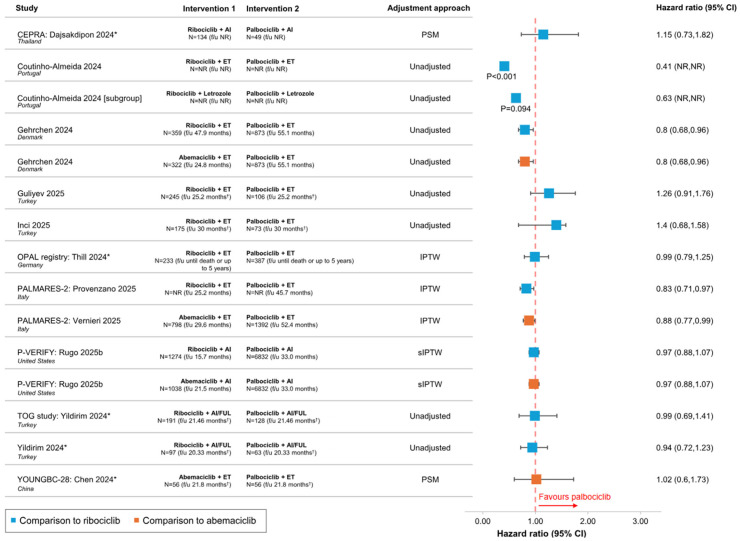
Forest plot of rwPFS hazard ratios for overall first-line population comparing ribociclib or abemaciclib versus palbociclib. * Reported hazard ratios and associated confidence intervals were inverted to align with reporting style from other studies. † Reported follow-up time for overall study cohort. Abbreviations: AIs = aromatase inhibitors; CI = confidence interval; ET = endocrine therapy; FUL = fulvestrant; IPTW = inverse probability of treatment weighting; NR = not reported; rwPFS = real-world progression-free survival; sIPTW = stabilized inverse probability of treatment weighting. References: [16,17,20,21,24,29,34,35,37,39,53,54].

Twenty-eight studies (31 records; 17 full-text and 14 abstract-only) reported rwPFS data for distinct groups of patients receiving palbociclib-based or ribociclib-based regimens in the first-line setting. Among these studies, rwPFS was comparable across CDK4/6i regimens (Figure 3). Thirteen studies directly compared palbociclib and ribociclib, with HRs reported to assess differences in rwPFS. The majority (*N* = 9) did not report a statistically significant difference in rwPFS, with HRs ranging from 0.63 [16] to 1.4 [17] in full-text studies and 0.88 [18] to 1.1 [19] in abstract-only studies. Common characteristics of these studies included imbalances in patient numbers between treatment groups, unadjusted analyses, and potential inaccuracies in electronic health record data capture. There were three full-text studies (HR: 0.41, 95% CI: NR; *p* < 0.001 [16]; HR: 0.83, 95% CI: 0.71–0.97; *p* = 0.02; [20] HR: 0.80, 95% CI: 0.68–0.96; *p* = 0.01 [21]) and one abstract-only study (HR: 0.57, 95% CI: 0.41–0.79; *p* < 0.001 [22]), which reported that treatment with ribociclib demonstrated statistically significant benefits in rwPFS compared to palbociclib. These findings should be interpreted in the context of the broader methodological heterogeneity observed across the entire evidence base, including variations in adjustment approaches, subgroup reporting, patient distributions, and follow-up duration.

Median rwPFS values were reported in several studies and provide important insight into the variability of results within studies and between studies, which may be related to variability in sample size and follow-up. For full-text articles, median rwPFS ranged from 20.7 months (*n* = 86) [23] to 32.0 months (*n* = 873) [21] for patients receiving palbociclib, and 22.9 months (*n* = 1274) [24] to 42.4 months (*n* = 359) [21] for patients receiving ribociclib. Among abstract-only studies, median rwPFS ranged from 16.0 months (*n* = 282) [25] to 47.0 months (*n* = 76) [26] for patients receiving palbociclib, and 14.0 months (*n* = 216) [25] to 48.0 months (*n* = 72) [26] for patients receiving ribociclib.

Where reported in studies with available full texts, median follow-up ranged widely; 33.0 [27] to 75.0 [28] months across palbociclib arms compared to 15.7 [27] to 64.4 [28] months for ribociclib-treated patients. Additional study details are available in Tables S4 and S10. Across subgroup analyses, rwPFS was broadly comparable across CDK4/6is, with limited statistically significant differences and substantial heterogeneity due to small sample sizes. A summary of subgroup results can be found in Supplementary Information S2.

##### Palbociclib vs. Abemaciclib

Fourteen studies (18 records; 9 full-text and 9 abstract-only) reported rwPFS data for distinct groups of patients receiving palbociclib-based or abemaciclib-based regimens in the first-line setting. Among these studies, rwPFS was comparable across CDK4/6i regimens (Figure 3). Five studies directly compared palbociclib and abemaciclib, with HRs reported to assess differences in rwPFS. Three studies showed no statistically significant difference in rwPFS, with HRs ranging from 0.96 [24] to 0.98 [29] and 0.80 [22] to 0.93 [22] in full-text studies and abstract-only studies, respectively. Unequal and small sample sizes were commonly observed across these studies. Two full-text studies showed that treatment with abemaciclib demonstrated statistically significant benefits in rwPFS compared to palbociclib (HR: 0.78, 95% CI: 0.64–0.95; *p* = 0.015; [20] HR: 0.74, 95% CI: 0.60–0.90; *p* = 0.005 [21]).

Notably, smaller sample sizes and shorter follow-up periods were observed in the abemaciclib treatment groups compared with palbociclib, likely reflecting the structural realities of temporal approval sequencing. These differences should be considered when interpreting the maturity and precision of estimates, but do not imply an inherent efficacy advantage or disadvantage for any agent. None of the abstract-only studies reported statistically significant benefits in rwPFS.

Reported median rwPFS values across studies offer important context for understanding variability in outcomes, potentially driven by differences in sample size and follow-up periods. For full-text articles, median rwPFS ranged from 20.7 months (*n* = 86) [23] to 32.2 months (*n* = 281) [30] for patients receiving palbociclib, and 12.4 months (*n* = 17) [23] to 48.7 months (*n* = 252) [30] for patients receiving abemaciclib. Among abstract-only studies, median rwPFS ranged from 16.0 months (*n* = 282) [25] to 47.0 months (*n* = 76) [26] for patients receiving palbociclib, and 16.0 months (*n* = 33) [31] to 23.2 months (*n* = 25) [32] for patients receiving abemaciclib.

Where reported in studies with available full texts, median follow-up ranged widely; 33.0 [27] to 75.0 [28] months across palbociclib arms, compared to 21.5 [27] to 40.28 [28] months in abemaciclib-treated patients. Additional study details are available in Tables S6 and S12. A summary of subgroup results can be found in Supplementary Information S2.

##### Ribociclib vs. Abemaciclib

Twelve studies (16 records; 7 full-text and 9 abstract-only) reported rwPFS data for distinct groups of patients receiving ribociclib-based or abemaciclib-based regimens in the first-line setting. Among these studies, rwPFS was comparable across CDK4/6i regimens (Supplementary Materials; Figure S2). Three studies compared ribociclib and abemaciclib. Two of these studies were full-text and showed comparable rwPFS, with HRs ranging from 0.91 [33] to 0.98 [24]. These studies had relatively short follow-up periods and unequal proportions of patients across treatment groups, which should be considered when interpreting rwPFS estimates. No full-text studies reported statistically significant benefits in rwPFS. One unadjusted abstract-only study reported that treatment with ribociclib demonstrated statistically significant benefits in rwPFS compared to abemaciclib (HR 0.61, 95% CI: 0.44–0.90; *p* = 0.011) [22].

For full-text articles, median rwPFS ranged from 22.9 months (*n* = 1274) [24] to 42.4 months (*n* = 359) [21] for patients receiving ribociclib, and 12.4 months (*n* = 17) [23] to 39.5 months (*n* = 56) [28] for patients receiving abemaciclib. Among abstract-only studies, median rwPFS ranged from 14.0 months (*n* = 216) [25] to 44.0 (*n* = 38) [31] for patients receiving ribociclib, and 16.0 months (*n* = 33) [31] to 48.0 months (*n* = 72) [26] for patients receiving abemaciclib.

Where reported in studies with available full texts, median follow-up ranged widely; 15.7 [27] to 64.4 [28] months across ribociclib arms compared to 21.5 [27] to 40.28 [28] months in abemaciclib-treated patients. Additional study details are available in Tables S8 and S14. A summary of subgroup results can be found in Supplementary Information S2.

#### 3.2.3. Overall Survival; Overall Population

##### Palbociclib vs. Ribociclib

**Figure 4:**
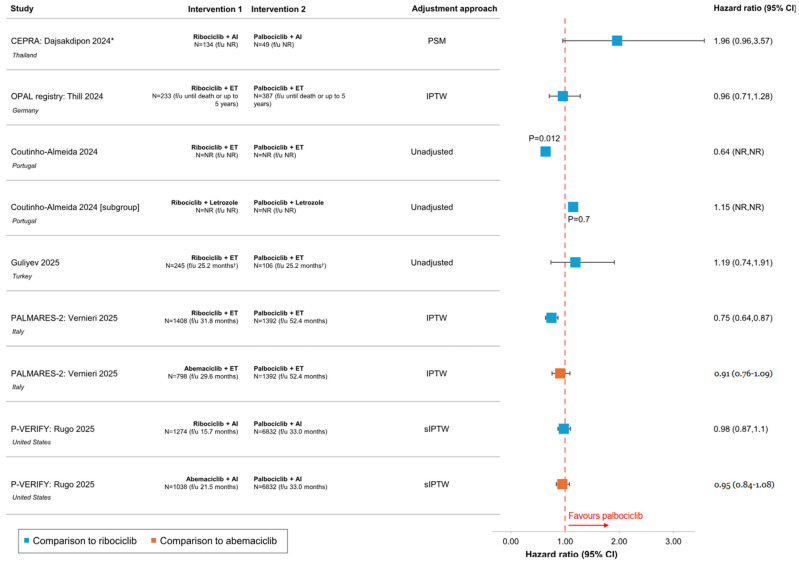
Forest plot of OS hazard ratios for overall first-line population comparing ribociclib or abemaciclib versus palbociclib. * Reported hazard ratios and associated confidence intervals were inverted to align with reporting style from other studies. ^†^ Reported follow-up time for overall study cohort. Abbreviations: AIs = aromatase inhibitors; CI = confidence interval; ET = endocrine therapy; IPTW = inverse probability of treatment weighting; NR = not reported; rwPFS = real-world progression-free survival; sIPTW = stabilized inverse probability of treatment weighting. References: [1,16,27,34,35,37,39].

Twenty-two studies (24 records; 12 full-text and 12 abstract-only) reported OS data for distinct groups of patients receiving palbociclib-based or ribociclib-based regimens in the first-line setting. Among these studies, OS was comparable across CDK4/6i regimens (Figure 4). Twelve studies compared palbociclib and ribociclib, with HRs reported to assess differences in OS. The majority of studies (*n* = 9) showed comparable OS, with HRs ranging from 0.51 [34] to 1.19 [35] in full-text studies and 0.72 [36] to 1.28 [19] in abstract-only studies. These studies commonly featured unequal patient distributions between treatments and tended to include unadjusted analyses. One full-text study (HR 0.75, 95% CI: 0.64–0.87, *p* < 0.01) [37] and two abstract-only studies (HR 0.65, 95% CI: 0.44–0.95, *p* = 0.026; [22] HR 1.44, 95% CI: 1.20–1.73 [38]) showed that treatment with ribociclib led to statistically significant benefits in OS outcomes.

Among full-text articles, median OS ranged from 42.4 months (*n* = 387) [39] to 85.8 months (*n* = 86) [23] for patients receiving palbociclib, and 49.3 months (*n* = 233) [39] to 59.0 months (*n* = 1274) [27] for patients receiving ribociclib. Among abstract-only studies, median OS ranged from 40.2 months (*n* = NR) [40] to 58.0 months (*n* = 608) [41] for patients receiving palbociclib, and 39.0 months (*n* = 159) [19] to 62.0 months (*n* = 72) [26] for patients receiving ribociclib.

Where reported in studies with available full texts, median follow-up ranged widely; 33.0 [27] to 75.0 [28] months across palbociclib arms compared to 15.7 [27] to 64.4 [28] months for ribociclib-treated patients. Additional details are available in Tables S4 and S10.

Overall survival outcomes were largely comparable across subgroup analyses, with few statistically significant differences and no consistent evidence of superiority; any trends were limited to select subgroups and constrained by heterogeneous and often small sample sizes. A summary of subgroup results can be found in Supplementary Information S2.

##### Palbociclib vs. Abemaciclib

Fifteen studies (16 records; 8 full-text and 8 abstract-only) reported OS data for distinct groups of patients receiving palbociclib-based or abemaciclib-based regimens in the first-line setting. Among these studies, OS was comparable across CDK4/6i regimens (Figure 4). Six studies compared palbociclib and abemaciclib, with HRs reported to assess differences in OS. The majority (*N* = 5) reported comparable OS, with HRs ranging from 0.91 [37] to 0.95 [27] and 0.90 [22] to 1.29 [41] in full-text studies and abstract-only studies, respectively. Unadjusted analyses and imbalanced sample sizes between treatment groups were commonly observed. No full-text studies reported statistically significant differences in OS. One abstract-only study demonstrated that abemaciclib treatment was associated with a statistically significant improvement in OS (HR 1.56, 95% CI: 1.165–2.091 [38]); as with other abstract-only evidence, interpretation is limited by the reduced level of methodological detail available, including incomplete reporting of follow-up duration.

Among full-text articles, median OS ranged from 44.3 months (*n* = 115) [42] to 85.8 months (*n* = 86) [23] for patients receiving palbociclib, and 34.3 months (*n* = 44) [42] to 64.5 months (*n* = 1038) [27] for patients receiving abemaciclib. Among abstract-only studies, median OS ranged from 43.8 months (*n* = 3504) [36] to 58.0 months (*n* = 608) [41] for patients receiving palbociclib, and 43.2 months (*n* = 575) [36] to 48.3 months (*n* = 341) [36] for patients receiving abemaciclib.

Where reported in studies with available full texts, median follow-up ranged widely; 33.0 [27] to 75.0 [28] months across palbociclib arms compared to 21.5 [27] to 40.28 [28] months in abemaciclib-treated patients. Additional details are available in Tables S6 and S12. A summary of subgroup results can be found in Supplementary Information S2.

##### Ribociclib vs. Abemaciclib

Fourteen studies (15 records; 7 full-text and 8 abstract-only) reported OS data for distinct groups of patients receiving ribociclib-based or abemaciclib-based regimens in the first-line setting. Among these studies, OS was comparable across CDK4/6i regimens (Figure S2). Four studies compared ribociclib and abemaciclib, with HRs ranging from 0.97 [27] to 1.21 [37] in full-text studies and 0.67 [22] to 1.10 [22] in abstract-only studies. As observed in other comparisons, group sizes varied across studies, which should be considered when interpreting the precision.

Among full-text articles, median OS ranged from 50.2 months (*n* = 46) [43] to 59.0 months (*n* = 1274) [27] for patients receiving ribociclib, and 34.3 months (*n* = 44) [42] to 64.5 months (*n* = 1038) [27] for patients receiving abemaciclib. Among abstract-only studies, median OS ranged from 44.2 months (*n* = 488) [36] to 62.0 months (*n* = 72) [26] for patients receiving ribociclib, and 43.2 months (*n* = 575) [36] to 48.3 months (*n* = 341) [36] for patients receiving abemaciclib.

Where reported in studies with available full texts, median follow-up ranged widely; 15.7 [27] to 64.4 [28] months across ribociclib arms, compared with 21.5 [27] to 40.28 [28] months in abemaciclib-treated patients.

Additional details are available in Tables S8 and S14. A summary of subgroup results can be found in Supplementary Information S2.

## 4. Discussion

Comparative insights observed in real-world settings complement RCTs by reflecting routine clinical practice and broader patient populations in the first-line treatment of HR+/HER2− a/mBC with CDK4/6i. This review synthesized RWE from 32 unique comparative studies examining the effectiveness of the three CDK4/6i, palbociclib, ribociclib, and abemaciclib, in combination with ET, and contrasted findings against multiple validated tools for assessing observational study quality.

Palbociclib comprised the largest number of patients (*n* = 11,273), and where reported, had approximately double the follow-up duration (33.0–75.0 months) compared to abemaciclib (21.5–40.28 months) or ribociclib (15.7–64.4 months). Palbociclib’s comparatively larger patient cohorts and more extended follow-up durations across the included real-world studies are exclusively attributable to its status as the first-in-class CDK4/6i to receive regulatory approval (February 2015), antedating ribociclib (March 2017) and abemaciclib (September 2017) by approximately two to two-and-a-half years. This temporal precedence generated a structural data advantage that is wholly an artifact of market chronology. It is essential to underscore that this de facto data advantage confers no intrinsic clinical superiority—it does not reflect, imply, or should not be construed as evidence of differential therapeutic efficacy relative to the subsequently approved agents. Greater real-world data volume is a consequence of earlier and more prolonged utilization in clinical practice, not of superior pharmacological activity or clinical performance.

Several methodological challenges arise when comparing CDK4/6i in real-world settings due to substantial heterogeneity in patient populations and potential channeling bias. Differences in age, menopausal status, ECOG performance, disease burden, and unknown confounders across treatment arms are rarely addressed adequately, as only a minority of studies applied robust adjustment methods such as multivariable adjustment, PSM, or IPTW. Incomplete and inconsistent reporting of baseline characteristics, combined with uneven representation of interventions, were noted. Finally, it is unclear whether rwPFS was measured consistently across studies.

The availability of comparative evidence in the form of HRs, to describe relative efficacy between CDK4/6i, was also inconsistently reported. Hazard ratios are the primary metric to compare treatments in time-to-event analyses because they take into account the entirety of the survival curve and censoring, as opposed to a single-point estimate like a median. However, of the 32 unique studies included, only 20 reported HRs and the majority were reported for palbociclib and ribociclib comparisons. This may be related to less frequent abemaciclib use in clinical practice and therefore smaller sample sizes, or limited follow-up.

Overall, the agents were broadly comparable in terms of rwPFS and OS benefits, with most HRs indicating no statistically significant differences. A minority of studies reported significant benefits with substantial heterogeneity, which may limit interpretation of findings. Additionally, there were various methodological limitations that would make comparisons between treatments challenging. Among the three studies reporting statistically significant HRs for rwPFS, common limitations included lack of adjustment for baseline characteristics, outcomes being derived from subgroup analyses, shorter follow-up, or small sample sizes for abemaciclib and ribociclib relative to palbociclib. These characteristics are best attributed to varying availability of treatments in the real-world setting, rather than to any clinical or pharmacological advantage of palbociclib. Similar issues were observed in the select studies reporting statistically significant benefits in OS. It should be noted that of five studies that did not show a significant difference, two had not adjusted for baseline characteristics. Additionally, OS was not the primary endpoint in most studies (*N* = 26), with implications for interpretation, particularly in prospective observational designs. Such methodological constraints underscore the need for cautious interpretation of apparent differences in effectiveness across RWE studies. Early signals of superiority observed in some studies (e.g., rwPFS in PALMARES-2) [33] can attenuate as follow-up matures, illustrating how initial findings can shift as data mature and reinforcing the importance of robust study design and adequate follow-up.

Given the expectation of methodological issues within these observational studies as noted above, all studies were assessed using the NOS, ISPOR-AMCP-NPC questionnaire, and ESMO-GROW checklist [13,14,15]. ISPOR-AMCP-NPC and ESMO-GROW assessments found that key elements such as study objectives, data sources, outcome definitions, and limitations were clearly reported. Notably, there were mixed results in the ISPOR-AMCP-NPC assessments regarding credibility of the results, considering factors such as sample size and statistical power. However, this does not indicate high methodological quality or credible comparative evidence. NOS [13] scores ranged from 4 to 9, with most studies being categorized as having low risk of bias. Notably, the NOS comparability domain is satisfied by any adjustment (e.g., basic multivariable models) and does not require implementation of advanced balancing techniques. Although these checklists provide a high-level understanding of broad reporting and methodological quality, they do not comprehensively assess risks of bias pertaining to specific survival estimates from real-world studies.

Design and analytic limitations were pervasive, including limited or absent adjustment for baseline imbalances, immature OS follow-up, and gaps in sample size justification, statistical power, and handling of missing data. Among 21 full-text studies, seven used IPTW or PSM, eight relied on multivariable analyses only, and six reported unadjusted comparisons. Given the non-randomized nature of RWE and prescribing biases, a lack of population-level adjustment introduces substantial risk of bias and limits the credibility of comparative findings. For these reasons, pooling HRs under such conditions, particularly when a substantial proportion of included studies employed unadjusted or minimally adjusted analyses, risks generating a spuriously precise aggregate estimate that obscures the very methodological limitations this review is designed to illuminate. The scientific value of this work resides in its capacity to transparently characterize the heterogeneity of the evidence base, rather than impose a false sense of homogeneity through meta-analytic aggregation. A formal quantitative meta-analysis restricted to studies employing robust adjustment methods such as IPTW or PSM would represent a meaningful extension of this work; however, such an analysis would only be feasible given a sufficient number of methodologically rigorous studies with adequately comparable designs and patient characteristics to permit valid pooling.

While most studies included in this review exhibited methodological limitations, a subset demonstrated characteristics that enhance credibility and interpretability. These studies typically featured larger sample sizes, longer and more balanced follow-up across treatment arms, and applied advanced confounding control methods. For example, the OPAL registry employed IPTW to mitigate baseline imbalances and reported prespecified analyses, strengthening confidence in its comparative estimates. Similarly, P-VERIFY is the largest real-world study to date (*n* = 9146) evaluating the comparative effectiveness of the three approved CDK4/6is, with both rwPFS and OS as primary endpoints [24]. This study also utilized standardized IPTW to account for differences in baseline characteristics, which further strengthens its credibility. PALMARES-2 also utilized IPTW to capture key outcomes of interest and include multiple key covariates [33]. In contrast, studies relying solely on unadjusted analyses or descriptive comparisons, particularly those with small cohorts or limited follow-up, offer less reliable insights. These distinctions underscore that not all RWE studies in the evidence base should contribute equally to the conclusions; methodological rigor is a critical determinant of credibility and requires a balance of multiple quality criteria.

When examining the comparative literature, it is also essential to consider varying aspects of the studies that influence the robustness and generalizability of results [55]. These may include sample size, follow-up duration, treatment combinations (e.g., aromatase inhibitors vs. fulvestrant), study design (prospective vs. retrospective), population differences and confounding adjustment, analytical approaches, censoring definitions, statistical balancing methods, patient selection (i.e., all eligible patients in an electronic health record vs. a subset of sites/physicians in a study), and transparency in reporting. These concerns are further compounded by the variability in how rwPFS is defined and measured across studies. Unlike progression endpoints in RCTs, rwPFS in observational settings is inherently complex, relying on routine clinical documentation rather than protocol-defined assessment schedules. Definitions vary considerably across data sources, health systems, and study teams, with differences in how progression events are identified, recorded, and adjudicated introducing a meaningful source of heterogeneity that limits direct comparability between studies. Many of these considerations are detailed in Table 4. Each of these factors can significantly impact the reliability and interpretation of results and their suitability for clinical decision-making. Most notable in this regard is the systematically unequal follow-up across treatment groups. The substantially longer observation periods in palbociclib arms (33.0–75.0 months) relative to ribociclib (15.7–64.4 months) and abemaciclib (21.5–40.28 months) introduce a form of time-dependent confounding that may differentially influence time-to-event estimates such as rwPFS and OS. Longer follow-up is inherently associated with greater event accrual, more mature survival curves, and potentially increased exposure to subsequent therapies, all of which can meaningfully affect the magnitude and direction of comparative effect estimates. Where follow-up disparities are particularly pronounced, apparent differences in survival outcomes between agents may therefore reflect data maturity in treatment arms with less follow-up rather than true differential efficacy. While these constraints are inherent to the RWE paradigm and are not unique to the present review, they appropriately limit the strength of comparative inference that can be drawn.

The SLR also identified several data gaps. Abemaciclib remains less frequently investigated than palbociclib or ribociclib, limiting precision in head-to-head comparisons. Subgroup reporting is sparse and inconsistent, and censoring definitions and rwPFS ascertainment vary across data sources. Most notably, several included studies did not report HRs to describe the comparative effectiveness of CDK4/6i agents. Furthermore, the included studies exhibit a marked geographic concentration, with the large majority derived from North American and Western European healthcare systems. Real-world treatment patterns, prescribing practices, patient population characteristics, access to CDK4/6i therapy, and healthcare infrastructure vary substantially across regions, and findings from this evidence base may not be directly transferable to patient populations in Asia, Latin America, the Middle East, or other underrepresented geographies. Addressing these gaps, along with standardizing outcome definitions and reporting analytic choices, will improve interpretability and transportability of comparative RWE.

We also acknowledge that prospective protocol registration—most notably via PROSPERO—represents an important and widely endorsed mechanism for enhancing the transparency, reproducibility, and credibility of systematic literature reviews, and we acknowledge this candidly. However, the key methodological components of this review, including the search strategy, eligibility criteria (i.e., PICOS framework), quality assessment instruments, and analytical approach, were defined a priori and applied consistently throughout the review process. But we acknowledge that the protocol was not prospectively registered in a public repository, and we recognize that this constitutes a limitation of the current work that warrants explicit disclosure.

## 5. Conclusions

Real-world comparative evidence indicates broadly similar effectiveness of CDK4/6i agents in first-line HR+/HER2− a/mBC. The majority of studies found no meaningful differences in OS or rwPFS outcomes. Select studies showing a difference in rwPFS and/or OS were primarily from unadjusted analyses and should be interpreted cautiously given heterogeneity and methodological limitations; the absence of head-to-head RCT data can lead one to examine data in the real world, which can be useful, but needs to have the appropriate context applied. Considerations include the heterogeneity of confounding across studies, and the inherent limitations of time-to-event ascertainment in routine clinical data collectively preclude any equivalence inference from the available evidence base. This review highlights the importance of evaluating study quality when interpreting comparative RWE. Continued generation of high-quality and methodologically robust RWE studies can help support the understanding of comparative effectiveness, especially in a setting where head-to-head RCT data does not exist, provided that appropriate methodological considerations are taken into account.

## Figures and Tables

**Table 1:** Review eligibility criteria.

Criteria	Inclusion Criteria	Exclusion Criteria
Population	Patients aged ≥18 years old with HR+/HER2− a/mBC. Pre- and post-menopausal women. Studies with ≥ 50% patients with ER+ or HR+ disease to be included. Alternatively, studies reporting outcomes separately for patients in these subgroups to be included.	Only patients aged < 18 years old. Women with early breast cancer (BC). Women with ER− or HR− BC if >50% of study population. Women with HER2+ BC if >50% of study population.
Intervention/comparators	Palbociclib, ribociclib, and abemaciclib in the first-line setting (in combination with endocrine therapy in the first-line setting).	Studies that do not include first-line CDK 4/6i therapy. Studies that do not specify line of therapy and/or CDK4/6i assessed were excluded. Studies that do not specify type of CDK 4/6i therapy.
Outcomes	Overall survival. Real-world progression-free survival.	Studies that do not report any relevant outcomes.
Study design	RWE studies (e.g., prospective and retrospective observational studies) reporting comparative data for at least two CDK4/6i arms. Published articles (1 January 2015 to 11 September 2025). Conference abstracts (1 January 2015 to 11 September 2025).	Any non-RWE studies. Studies enrolling <100 patients or <50 patients within all studied treatment groups.
Language	Articles in English ^†^	All non-English articles

^†^ Citation retrieval was not limited by language. Records were categorized based on language during study selection, and non-English records were excluded. Abbreviations: a/mBC = advanced/metastatic breast cancer; CDK4/6i = cyclin-dependent kinase inhibitor; ER = estrogen receptor; HER2 = human epidermal growth factor receptor 2; HR = hormone receptor; RWE = real-world evidence.

**Table 2:** Summary of quality assessments.

Assessment Tool	Domain/Item *	Fully Sufficient (*N* = 21) **	Partially Sufficient (*N* = 21) ^†^	Common Issues Identified
Newcastle–Ottawa Scale	Selection	86% (18/21)	14% (3/21)	Most studies had representative cohorts and secure exposure ascertainment.
	Comparability	81% (17/21)	0% (0/21)	Limited adjustment for key confounders; studies claimed control for confounders but lacked robust statistical methods (e.g., multivariate analysis or propensity matching).
	Outcome assessment	19% (4/21)	57% (12/21)	Some studies lacked clear follow-up adequacy or description of lost to follow-up; blinding rarely reported.
ESMO-GROW checklist	Title	81% (17/21)	14% (3/21)	Some studies did not report title concisely or lacked key terms like “real-world” or “observational.”
	Introduction	95% (20/21)	5% (1/21)	One study lacked complete scientific rationale or background detail.
	Methods; General	5% (1/21)	90% (19/21)	Missing details on study objectives and data sources.
	Methods; Variables and Statistical Methods	0% (0/21)	48% (10/21)	Missing details on interoperability, sample size, bias mitigation, and validity checks.
	Results	29% (6/21)	71% (15/21)	Missing transparency in sample selection (i.e., incomplete flowcharts and exclusion details)
	Discussion and Conclusions	52% (11/21)	48% (10/21)	Missing details on generalizability and balanced summary.
	Final Considerations	71% (15/21)	19% (4/21)	Missing funding details or incomplete acknowledgements.
ISPOR-AMCP-NPC questionnaire	Relevance	100% (21/21)	0% (0/21)	None identified.
	Credibility	5% (1/21)	86% (17/21)	Sample size, statistical power, and sufficient follow-up to detect differences were not addressed.
	Data	67% (14/21)	19% (4/21)	Source, exposure, outcome, and follow-up details not sufficient in some records.
	Analyses	48% (10/21)	24% (5/21)	Missing sensitivity analyses and assessment of confounders.
	Reporting	19% (4/21)	81% (17/21)	Confounder-adjusted estimates of treatment effects and extent of missing data were not often reported.
	Interpretation	71% (15/21)	29% (6/21)	Some treatment effects were not clinically meaningful, and the impact of unmeasured confounding was not discussed.
	Conflicts of Interest	38% (8/21)	52% (11/21)	Most records addressed conflicts of interest but may still cause a bias.

* Domains listed reflect specific criteria listed within each assessment tool. Orange highlighted cells contain domains/items for which the proportion of assessed studies was <50% for Fully Sufficient. ** Full compliance required 100% of questions in the domain to meet acceptable criteria. ^†^ Partial compliance required ≥50% of questions in the domain to meet acceptable criteria. Abbreviations: ESMO-GROW = European Society for Medical Oncology Guidance for Reporting Oncology real-World Evidence; ISPOR-AMCP-NPC = Professional Society for Health Economics and Outcomes Research–Academy of Managed Care Pharmacy–National Pharmaceutical Council.

**Table 3:** Additional characteristics relevant to assessing risk of bias.

Domain	Metric	Definition/Threshold	Description Across Studies (*N* = 21)
Sample size	Total (overall cohort)	≥100 patients	100% (21/21); sample sizes varied widely, ranging from *n* = 121 to 9146
	Per-arm	At least one treatment arm includes ≥50 patients	100% (21/21); sample sizes ranged widely within and between arms: Palbociclib: *n* = 49 to 6832 Ribociclib: *n* = 28 to 1408 Abemaciclib: *n* = 17 to 1038
Follow-up time	Reported	--	86% (18/21)
	Insufficient (overall cohort)	<12 months follow-up	14% (3/21)
	Length of follow-up per-arm	--	The follow-up length for palbociclib groups was generally longer compared to the other arms: Palbociclib: 33.0–75.0 months Ribociclib: 15.7–64.4 months Abemaciclib: 21.5–40.28 months
Adjustment methods	Unadjusted analyses	No multivariable analyses or matching	29% (6/21)
	Multivariable regression only	Covariate adjustment without propensity methods	38% (8/21)
	Propensity score matching	Pairwise matching	14% (3/21)
	IPTW/sIPTW	Inverse probability of treatment weighting	19% (4/21)

Abbreviations: IPTW = inverse probability of treatment weighting; sIPTW = stabilized inverse probability of treatment weighting.

**Table 4:** Key determinants of study validity and implications in the current evidence base.

Type of Variation	Impact on Validity	Key Considerations	Impact on the Current Synthesis of 1 L CDK4/6i Comparisons
Transparency of information [56]	Where key methodological and data details are incomplete, it limits the ability to appropriately ascertain quality, and results could be misattributed without appropriate context.	If a key detail is unavailable (e.g., whether OS is a validated endpoint within the dataset), consider whether the study should be used to inform on this endpoint.	Notable reduction in the evidence base size upon removing studies that did not report on follow-up time or other key details.
Sample sizes [57]	Small sample sizes may increase variance and yield less precise estimates.	Consider implementing a minimum cut-off for sample size or powering for the endpoint.	SLR protocol defined a cut-off of ≥100 patients, which limited heterogeneity. Generally, the range of sample sizes from included studies was deemed intermediate; a smaller number of patients in ribociclib and abemaciclib groups increases uncertainty.
Time period [58]	Variation in enrollment periods can introduce clinical heterogeneity due to shifts in standard of care, diagnostic approaches, or treatment availability.	Assess using clinical guidance whether temporal differences may introduce bias when comparing across studies.	Enrollment periods ranged from 2004 * to 2024. Notably, there were multiple studies identified that compared all three approved CDK4/6i agents.
Confounding variables [59]	Lack of adjustment introduces confounding; potentially confounding by indication whereby physicians’ treatment choice is influenced by patient traits. Mixing adjusted and unadjusted analyses can bias pooled effect size.	Prioritize adjusted estimates when available, with unadjusted analyses being used supportively.	Notable reduction in the evidence base size upon removing studies that did not utilize adjustments. Channeling bias; less frail patients preferentially receiving ribociclib and abemaciclib. Without adequate adjustments, comparisons cannot be made.
Follow-up time [60]	Very different follow-up times impact treatment effect estimates and may lead to time-dependent bias when comparing multiple studies. Longer follow-up may also be associated with use of additional therapies.	Harmonize follow-up periods into groups or implement a minimum cut-off to ensure adequate event capture. Verify if proportional hazards assumption is met, which indicates minimal risk.	One outlier study was identified, reporting results for up to five years follow-up. The range of follow-up from the remaining included studies was deemed intermediate; differences observed between longer follow-up length of palbociclib treatment compared to other CDK4/6is. Generally, proportional hazards assumption was considered to be met based on study reporting.

* CDK4/6i were not available until 2015; however, some studies included enrollment periods prior to this date (e.g., electronic medical records covering 2004 to 2024). Abbreviations: CDK6/4i = cyclin-dependent kinase 4/6 inhibitor; OS = overall survival; SLR = systematic literature review.

## Data Availability

No new data were created in this study. Additional information regarding the data synthesis may be available upon written request to the authors.

## Supplementary Materials

The following supporting information can be downloaded at: https://www.mdpi.com/article/10.3390/cancers18142362/s1, Table S1: PRISMA 2020 checklist; Supplementary Information S1: Most recent search strategy; Figure S1: PRISMA flow diagrams; Table S2: List of included studies; File S1: Quality assessments; Table S3: List of subgroups of interest; Table S4: Progression-free survival and overall survival for first-line palbociclib in comparative RWE studies versus ribociclib (overall population; studies with manuscript data available); Table S5: Progression-free survival and overall survival for first-line palbociclib in comparative RWE studies versus ribociclib (subgroups; studies with manuscript data available); Table S6: Progression-free survival and overall survival for first-line palbociclib in comparative RWE studies versus abemaciclib (overall population; studies with manuscript data available); Table S7: Progression-free survival and overall survival for first-line palbociclib in comparative RWE studies versus abemaciclib (subgroups; studies with manuscript data available); Table S8: Progression-free survival and overall survival for first-line ribociclib in comparative RWE studies versus abemaciclib (overall population; studies with manuscript data available); Table S9; Progression-free survival and overall survival for first-line ribociclib in comparative RWE studies versus abemaciclib (subgroups; studies with manuscript data available); Table S10: Progression-free survival and overall survival for first-line palbociclib in comparative RWE studies versus ribociclib (overall population; studies with only abstract data available); Table S11: Progression-free survival and overall survival for first-line palbociclib in comparative RWE studies versus ribociclib (subgroups; studies with only abstract data available); Table S12: Progression-free survival and overall survival for first-line palbociclib in comparative RWE studies versus abemaciclib (overall population; studies with only abstract data available); Table S13: Progression-free survival and overall survival for first-line palbociclib in comparative RWE studies versus abemaciclib (subgroups; studies with only abstract data available); Table S14: Progression-free survival and overall survival for first-line ribociclib in comparative RWE studies versus abemaciclib (overall population; studies with only abstract data available); Table S15: Progression-free survival and overall survival for first-line ribociclib in comparative RWE studies versus abemaciclib (subgroups; studies with only abstract data available); Supplementary Information S2: Summary of subgroup OS and PFS results; Table S16: Full-text study definitions of rwPFS; Figure S2: Forest plot of OS and PFS hazard ratios for overall first-line abemaciclib versus ribociclib.
