# Supplementary material for: A Systematic Literature Review of the Effectiveness of CDK 4/6 Inhibitors for First-Line Treatment of HR+/HER2− Advanced/Metastatic Breast Cancer: A Comparison of Real-World Evidence
Source: Cancers (Basel). 2026 Jul 22;18(14):2362. doi: 10.3390/cancers18142362 (PMC13406782; doi:10.3390/cancers18142362)
Supplement: Supplementary file 1 [file cancers-18-02362-s001.zip › cancers-4382851-supplementary.pdf]

Table of Contents

Table S1. PRISMA 2020 checklist 3

Supplementary Information S1: Most recent search strategy 6

Figure S1. PRISMA flow diagrams 17

Table S2. List of included studies 22

File S1. Quality assessments 29

Table S3. List of subgroups of interest 32

Table S4. Progression-free survival and overall survival for first-line palbociclib in comparative RWE studies versus ribociclib (overall population; studies with manuscript data available) 33

Table S5. Progression-free survival and overall survival for first-line palbociclib in comparative RWE studies versus ribociclib (subgroups; studies with manuscript data available) 39

Table S6. Progression-free survival and overall survival for first-line palbociclib in comparative RWE studies versus abemaciclib (overall population; studies with manuscript data available) 60

Table S7. Progression-free survival and overall survival for first-line palbociclib in comparative RWE studies versus abemaciclib (subgroups; studies with manuscript data available) 64

Table S8. Progression-free survival and overall survival for first-line ribociclib in comparative RWE studies versus abemaciclib (overall population; studies with manuscript data available) 84

Table S9. Progression-free survival and overall survival for first-line ribociclib in comparative RWE studies versus abemaciclib (subgroups; studies with manuscript data available) 87

Table S10. Progression-free survival and overall survival for first-line palbociclib in comparative RWE studies versus ribociclib (overall population; studies with only abstract data available) 106

Table S11. Progression-free survival and overall survival for first-line palbociclib in comparative RWE studies versus ribociclib (subgroups; studies with only abstract data available) 110

Table S12. Progression-free survival and overall survival for first-line palbociclib in comparative RWE studies versus abemaciclib (overall population; studies with only abstract data available) 112

Table S13. Progression-free survival and overall survival for first-line palbociclib in comparative RWE studies versus abemaciclib (subgroups; studies with only abstract data available) 115

Table S14. Progression-free survival and overall survival for first-line ribociclib in comparative RWE studies versus abemaciclib (overall population; studies with only abstract data available) 116

Table S15. Progression-free survival and overall survival for first-line ribociclib in comparative RWE studies versus abemaciclib (subgroups; studies with only abstract data available) .. 119

Supplementary Information S2. Summary of subgroup OS and PFS results 120

Table S16. Full-text study definitions of rwPFS 122

Figure S2. Forest plot of OS and PFS hazard ratios for overall first-line abemaciclib versus ribociclib 124

Table S1. PRISMA 2020 checklist

Section and topic	Item #	Checklist item	Location where item is reported
**Title**			
Title	1	Identify the report as a systematic review.	Title page
**Abstract**			
Abstract	2	See the PRISMA 2020 for Abstracts checklist.	Abstract
**Introduction**			
Rationale	3	Describe the rationale for the review in the context of existing knowledge.	Introduction
Objectives	4	Provide an explicit statement of the objective(s) or question(s) the review addresses.	Introduction
**Methods**			
Eligibility criteria	5	Specify the inclusion and exclusion criteria for the review and how studies were grouped for the syntheses.	Methods 2.2 & Table 1
Information sources	6	Specify all databases, registers, websites, organizations, reference lists, and other sources searched or consulted to identify studies. Specify the date when each source was last searched or consulted.	Methods 2.1
Search strategy	7	Present the full search strategies for all databases, registers, and websites, including any filters and limits used.	Appendix B
Selection process	8	Specify the methods used to decide whether a study met the inclusion criteria of the review, including how many reviewers screened each record and each report retrieved, whether they worked independently, and, if applicable, details of automation tools used in the process.	Methods 2.2
Data collection process	9	Specify the methods used to collect data from reports, including how many reviewers collected data from each report, whether they worked independently, any processes for obtaining or confirming data from study investigators, and, if applicable, details of automation tools used in the process.	Methods 2.2
Data items	10a	List and define all outcomes for which data were sought. Specify whether all results that were compatible with each outcome domain in each study were sought (e.g., for all measures, time points, analyses), and if not, the methods used to decide which results to collect.	Methods 2.2
	10b	List and define all other variables for which data were sought (e.g., participant and intervention characteristics, funding sources). Describe any assumptions made about any missing or unclear information.	Methods 2.2
Study risk of bias assessment	11	Specify the methods used to assess risk of bias in the included studies, including details of the tool(s) used, how many reviewers assessed each study and whether they worked independently, and if applicable, details of automation tools used in the process.	Methods 2.2
Effect measures	12	Specify for each outcome the effect measure(s) (e.g., risk ratio, mean difference) used in the synthesis or presentation of results.	Methods 2.3

Section and topic	Item #	Checklist item	Location where item is reported
Synthesis methods	13a	Describe the processes used to decide which studies were eligible for each synthesis (e.g., tabulating the study intervention characteristics and comparing against the planned groups for each synthesis (item #5)).	Methods 2.3
	13b	Describe any methods required to prepare the data for presentation or synthesis, such as handling of missing summary statistics or data conversions.	Methods 2.3
	13c	Describe any methods used to tabulate or visually display results of individual studies and syntheses.	Methods 2.3
	13d	Describe any methods used to synthesize results and provide a rationale for the choice(s). If meta-analysis was performed, describe the model(s), method(s) to identify the presence and extent of statistical heterogeneity, and software package(s) used.	Methods 2.3
	13e	Describe any methods used to explore possible causes of heterogeneity among study results (e.g., subgroup analysis, meta-regression).	Methods 2.2 and 2.3
	13f	Describe any sensitivity analyses conducted to assess robustness of the synthesized results.	N/A
Reporting bias assessment	14	Describe any methods used to assess risk of bias due to missing results in a synthesis (arising from reporting biases).	N/A
Certainty assessment	15	Describe any methods used to assess certainty (or confidence) in the body of evidence for an outcome.	N/A
**Results**			
Study selection	16a	Describe the results of the search and selection process, from the number of records identified in the search to the number of studies included in the review, ideally using a flow diagram.	Results 3.1 & Figure 1
	16b	Cite studies that might appear to meet the inclusion criteria but which were excluded, and explain why they were excluded.	Results 3.1
Study characteristics	17	Cite each included study and present its characteristics.	Results 3.1 and 3.3.1
Risk of bias in studies	18	Present assessments of risk of bias for each included study.	Results 3.2; Tables 2–3
Results of individual studies	19	For all outcomes, present, for each study: (a) summary statistics for each group (where appropriate) and (b) an effect estimate and its precision (e.g., confidence/credible interval), ideally using structured tables or plots.	Results 3.3; Appendices G-I
Results of syntheses	20a	For each synthesis, briefly summarise the characteristics and risk of bias among contributing studies.	Results 3.3
	20b	Present results of all statistical syntheses conducted. If meta-analysis was done, present for each the summary estimate and its precision (e.g., confidence/credible interval) and measures of statistical heterogeneity. If comparing	N/A

Section and topic	Item #	Checklist item	Location where item is reported
		groups, describe the direction of the effect.	
	20c	Present results of all investigations of possible causes of heterogeneity among study results.	Results 3.2
	20d	Present results of all sensitivity analyses conducted to assess the robustness of the synthesized results.	N/A
Reporting biases	21	Present assessments of risk of bias due to missing results (arising from reporting biases) for each synthesis assessed.	N/A
Certainty of evidence	22	Present assessments of certainty (or confidence) in the body of evidence for each outcome assessed.	N/A
**Discussion**			
Discussion	23a	Provide a general interpretation of the results in the context of other evidence.	Discussion 4
	23b	Discuss any limitations of the evidence included in the review.	Discussion 4
	23c	Discuss any limitations of the review processes used.	Discussion 4
	23d	Discuss implications of the results for practice, policy, and future research.	Discussion 4
**Other information**			
Registration and protocol	24a	Provide registration information for the review, including register name and registration number, or state that the review was not registered.	Methods 2.1
	24b	Indicate where the review protocol can be accessed or state that a protocol was not prepared.	Methods 2.1
	24c	Describe and explain any amendments to information provided at registration or in the protocol.	N/A
Support	25	Describe sources of financial or nonfinancial support for the review and the role of the funders or sponsors in the review.	Funding
Competing interests	26	Declare any competing interests of review authors.	Conflicts of Interest
Availability of data, code. and other materials	27	Report which of the following are publicly available and where they can be found: template data collection forms; data extracted from included studies; data used for all analyses; analytic code; any other materials used in the review.	Appendices

For more information, visit: http://www.prisma-statement.org/

Supplementary Information S1: Most recent search strategy

Date of the search: 11 Sept 2025

Update date span: (based on the update schedules of the included databases)

MEDLINE, Embase & CDSR: June 2025 – Current

CENTRAL & CDSR: May 2025 – Current

Database searched (using OvidSP platform):

MEDLINE® and Epub Ahead of Print, In-Process, In-Data-Review & Other Non-Indexed Citations, Daily and Versions (1946 to present)

Embase (1974 – present)

EBM Reviews - Cochrane Central Register of Controlled Trials (1991 – present)

EBM Reviews - Cochrane Database of Systematic Reviews (2005 – present)

Filters: none

Limits:

Humans only

Adults only

Summary of Results:

**CDK4/6i SLR Search**	**Results deduped in Ovid**	**Results deduped in EndNote**
Update #11 (May/June 2025 – Current)	698	665

Search history saved in Corner3 as:

Breast Cancer - Palbociclib - Multifile - UPDATE 11 - 120 003873 001 - FINAL-proposal

11 Sept 2025 - Liz H, Krista, Sofiya, Imy - UPDATE #11 (May/June 2025 – Current)

MULTIFILE SEARCH

Database(s): Embase 1974 to 2025 September 10, Ovid MEDLINE® and Epub Ahead of Print, In-Process, In-Data-Review & Other Non-Indexed Citations, Daily and Versions 1946 to September 10, 2025, EBM Reviews - Cochrane Central Register of Controlled Trials August 2025, EBM Reviews - Cochrane Database of Systematic Reviews 2005 to September 3, 2025

Search Strategy:

#	Searches	Results

1	exp Breast Neoplasms/	1156320
2	exp Breast Carcinoma In Situ/	36932
3	((breast$1 or mamma or mammary) adj3 (adenocarcinoma* or cancer* or carcinoma* or neoplasm* or tumour* or tumor*)).tw,kf.	1210757
4	((ductal or intraductal or intra-ductal) adj (carcinoma? or hyperplasia?)).tw,kf.	54033
5	DCIS.tw,kf.	19955
6	(lobul* carcinoma? adj2 “in situ”).tw,kf.	3175
7	LCIS.tw,kf.	2103
8	(paget* and (areola? or breast* or mammary or nipple*)).tw,kf.	3676
9	or/1-8 [BREAST CANCER]	1454387
10	Receptor, ErbB-2/	122584
11	ERBB2 protein, human.nm.	15069
12	(ErbB2 or “ErbB 2” or HER2* or “HER 2*” or “c-ErbB2” or “C-ErbB 2”).tw,kf.	194142
13	((oncoprotein* or onco-protein* or protein* or receptor*) adj1 (neu or neuregulin)).tw,kf.	3334
14	CD340.tw,kf.	52
15	(“p185(c-neu)” or p185erbB).tw,kf.	90
16	(neu protooncogene or neu proto-oncogene).tw,kf.	459
17	NGL.tw,kf.	2106
18	metastatic lymph node gene 19.tw,kf.	0
19	(MLN19 or MLN 19).tw,kf.	2
20	(human adj1 “epidermal growth factor receptor 2”).tw,kf.	34618
21	ErbB Receptors/	176577
22	or/10-21	384369
23	9 and 22 [HER2 BREAST CANCER]	175156
24	exp Receptors, Estrogen/	180102
25	estrogen receptor?.tw,kf.	157246
26	oestrogen receptor?.tw,kf.	16821

27	ERalpha*.tw,kf.	31211
28	ERbeta.tw,kf.	15922
29	ER positive.tw,kf.	22889
30	“33 a”.tw,kf.	11826
31	“luminal b”.tw,kf.	9968
32	(luminal adj2 subtype*).tw,kf.	7520
33	hormone receptor?.tw,kf.	103846
34	HR positive.tw,kf.	5954
35	progesterone receptor?.tw,kf.	63602
36	progestin receptor?.tw,kf.	1951
37	PR positive.tw,kf.	4788
38	or/24-37	361348
39	9 and 38 [HR BREAST CANCER]	188513
40	exp Breast Neoplasms/sc [secondary/metastatic]	5326
41	(advanced adj3 ((breast$1 or mamma or mammary) adj3 (adenocarcinoma* or cancer* or carcinoma* or neoplasm* or tumour* or tumor*))).tw,kf.	46282
42	(metastatic* adj3 ((breast$1 or mamma or mammary) adj3 (adenocarcinoma* or cancer* or carcinoma* or neoplasm* or tumour* or tumor*))).tw,kf.	85069
43	(metastas* adj3 ((breast$1 or mamma or mammary) adj3 (adenocarcinoma* or cancer* or carcinoma* or neoplasm* or tumour* or tumor*))).tw,kf.	44010
44	(progressive adj3 ((breast$1 or mamma or mammary) adj3 (adenocarcinoma* or cancer* or carcinoma* or neoplasm* or tumour* or tumor*))).tw,kf.	712
45	(secondary adj3 ((breast$1 or mamma or mammary) adj3 (adenocarcinoma* or cancer* or carcinoma* or neoplasm* or tumour* or tumor*))).tw,kf.	3452
46	(terminal adj3 ((breast$1 or mamma or mammary) adj3 (adenocarcinoma* or cancer* or carcinoma* or neoplasm* or tumour* or tumor*))).tw,kf.	177
47	or/40-46 [METASTATIC BREAST CANCER]	159601
48	23 or 39 or 47 [BREAST CANCERS OF INTEREST]	385916
49	exp Infant/ not exp Adult/	1958469
50	exp Child/ not exp Adult/	3994275

51	Adolescent/ not exp Adult/	1522844
52	48 not (49 or 50 or 51) [INFANT-, CHILD-, ADOLESCENT-ONLY REMOVED]	385031
53	Palbociclib.nm.	1247
54	(palbociclib or ibrance$2 or palbociclib or “pd 0332991” or “pd 0332991” or pd332991or pd332991 or “pf 00080665” or pf00080665).tw,kf.	9507
55	ribociclib.nm.	471
56	(ribociclib or kisqali$2 or “lee 011” or lee 011a or lee 011bba or lee 11 or lee 11a or lee 11bba or lee011 or lee011a or lee011bba or lee11 or lee11a or lee11bba).tw,kf.	4362
57	(abemaciclib or bemaciclib$2 or ly 2835219 or ly2835219 or verzenio$2).tw,kf.	4614
58	Cyclin-Dependent Kinase Inhibitor Proteins/	9984
59	cyclin-dependent kinase inhibitor?.tw,kf.	17023
60	(CDKI or CDKIs).tw,kf.	1686
61	CKI Protein?.tw,kf.	47
62	(CIP-KIP adj2 protein?).tw,kf.	91
63	Cyclin-Dependent Kinases/	24358
64	Cyclin-Dependent Kinase 4/	22740
65	Cyclin-Dependent Kinase 6/	13868
66	(Cyclin-Dependent Kinase 4 or Cyclin-Dependent Kinase 6).tw,kf.	7441
67	(CDK4* or CDK 4* or CDK6* or CDK 6*).tw,kf.	38491
68	(Cell Division Protein Kinase 4 or PSK-J3 Kinase or PSKJ3 Kinase or p34PSK-J3 Kinase or p34PSKJ3 Kinase).tw,kf.	6
69	(Cell Division Protein Kinase 6 or PLSTIRE Protein).tw,kf.	12
70	or/53-69 [FIRST LINE DRUGS]	89859
71	52 and 70 [BREAST CANCERS OF INTEREST - FIRST LINE DRUGS]	15125
72	exp Animals/ not Humans/	11357920
73	71 not 72 [ANIMAL-ONLY REMOVED]	14917
74	limit 73 to yr=”2023-current”	5768
75	74 use ppezv [MEDLINE RECORDS]	1473

76	exp breast cancer/	1066455
77	((breast$1 or mamma or mammary) adj3 (adenocarcinoma* or cancer* or carcinoma* or neoplasm* or tumour* or tumor*)).tw,kw.	1191803
78	((ductal or intraductal or intra-ductal) adj (carcinoma? or hyperplasia?)).tw,kw.	53137
79	DCIS.tw,kw.	19856
80	(lobul* carcinoma? adj2 “in situ”).tw,kw.	3090
81	LCIS.tw,kw.	2093
82	(paget* and (areola? or breast* or mammary or nipple*)).tw,kw.	3680
83	or/76-82 [BREAST CANCER]	1420114
84	epidermal growth factor receptor 2/	88332
85	(ErbB2 or “ErbB 2” or HER2* or “HER 2*” or “c-ErbB2” or “C-ErbB 2”).tw,kw.	193877
86	((oncoprotein* or onco-protein* or protein* or receptor*) adj1 (neu or neuregulin)).tw,kw.	3318
87	CD340.tw,kw.	52
88	(“p185(c-neu)” or p185erbB).tw,kw.	90
89	(neu protooncogene or neu proto-oncogene).tw,kw.	458
90	NGL.tw,kw.	2100
91	metastatic lymph node gene 19 protein*.tw,kw.	0
92	(MLN19 or MLN 19).tw,kw.	2
93	(human adj1 “epidermal growth factor receptor 2”).tw,kw.	34790
94	or/84-93	226064
95	83 and 94 [HER2 BREAST CANCER]	160185
96	exp estrogen receptor/	180102
97	estrogen receptor?.tw,kw.	155970
98	oestrogen receptor?.tw,kw.	16698
99	ERalpha*.tw,kw.	31182
100	ERbeta.tw,kw.	15891
101	exp estrogen receptor positive breast cancer/	15069

102	ER positive.tw,kw.	22791
103	“luminal a”.tw,kw.	11826
104	“luminal b”.tw,kw.	9966
105	(luminal adj2 subtype*).tw,kw.	7451
106	hormone receptor?.tw,kw.	101115
107	progesterone receptor positive breast cancer/	2616
108	HR positive.tw,kw.	5877
109	progesterone receptor?.tw,kw.	63187
110	PR positive.tw,kw.	4762
111	progestin receptor?.tw,kw.	1944
112	or/96-111	361981
113	83 and 112 [HR BREAST CANCER]	190897
114	metastatic breast cancer/	36311
115	(advanced adj3 ((breast$1 or mamma or mammary) adj3 (adenocarcinoma* or cancer* or carcinoma* or neoplasm* or tumour* or tumor*))).tw,kw.	45798
116	(metastatic* adj3 ((breast$1 or mamma or mammary) adj3 (adenocarcinoma* or cancer* or carcinoma* or neoplasm* or tumour* or tumor*))).tw,kw.	84079
117	(metastas* adj3 ((breast$1 or mamma or mammary) adj3 (adenocarcinoma* or cancer* or carcinoma* or neoplasm* or tumour* or tumor*))).tw,kw.	44130
118	(progressive adj3 ((breast$1 or mamma or mammary) adj3 (adenocarcinoma* or cancer* or carcinoma* or neoplasm* or tumour* or tumor*))).tw,kw.	711
119	(secondary adj3 ((breast$1 or mamma or mammary) adj3 (adenocarcinoma* or cancer* or carcinoma* or neoplasm* or tumour* or tumor*))).tw,kw.	3414
120	(terminal adj3 ((breast$1 or mamma or mammary) adj3 (adenocarcinoma* or cancer* or carcinoma* or neoplasm* or tumour* or tumor*))).tw,kw.	180
121	or/114-120 [METASTATIC BREAST CANCER]	163947
122	95 or 113 or 121 [BREAST CANCERS OF INTEREST]	380697
123	adolescent/ not exp adult/	1522844
124	child/ not exp adult/	2912784
125	infant/ not exp adult/	1248377

126	fetus/ not exp adult/	253341
127	122 not (123 or 124 or 125 or 126) [FETUS-, INFANT-, CHILD-, ADOLESCENT-ONLY REMOVED]	379836
128	palbociclib/	9634
129	(palbociclib or ibrance$2 or palbociclib or “pd 0332991” or “pd 0332991” or pd332991or pd332991 or “pf 00080665” or pf00080665).tw,kw.	9496
130	ribociclib/	4225
131	(ribociclib or kisqali$2 or “lee 011” or lee 011a or lee 011bba or lee 11 or lee 11a or lee 11bba or lee011 or lee011a or lee011bba or lee11 or lee11a or lee11bba).tw,kw.	4354
132	abemaciclib/	4654
133	(abemaciclib or bemaciclib$2 or ly 2835219 or ly2835219 or verzenio$2).tw,kw.	4602
134	cyclin dependent kinase inhibitor/	9198
135	cyclin-dependent kinase inhibitor?.tw,kw.	16746
136	(CDKI or CDKIs).tw,kw.	1661
137	CKI protein?.tw,kw.	47
138	(CIP-KIP adj2 protein?).tw,kw.	90
139	cyclin dependent kinase/	24358
140	cyclin dependent kinase 4/	22740
141	cyclin dependent kinase 6/	13868
142	(Cyclin-Dependent Kinase 4 or Cyclin-Dependent Kinase 6).tw,kw.	7263
143	(CDK4* or CDK 4* or CDK6* or CDK 6*).tw,kw.	38445
144	(Cell Division Protein Kinase 4 or PSK-J3 Kinase or PSKJ3 Kinase or p34PSK-J3 Kinase or p34PSKJ3 Kinase).tw,kw.	6
145	(Cell Division Protein Kinase 6 or PLSTIRE Protein).tw,kw.	12
146	or/128-145 [FIRST LINE DRUGS]	92304
147	127 and 146 [BREAST CANCERS OF INTEREST - FIRST LINE DRUGS]	16042
148	exp animal/ or exp animal experimentation/ or exp animal model/ or exp animal experiment/ or nonhuman/ or exp vertebrate/	66107310
149	exp human/ or exp human experimentation/ or exp human experiment/	52862742

150	148 not 149	13247261
151	147 not 150 [ANIMAL-ONLY REMOVED]	15750
152	limit 151 to yr=”2023-current”	6122
153	152 use oemezd [EMBASE RECORDS]	4081
154	exp Breast Neoplasms/	1156320
155	exp Breast Carcinoma In Situ/	36932
156	((breast$1 or mamma or mammary) adj3 (adenocarcinoma* or cancer* or carcinoma* or neoplasm* or tumour* or tumor*)).ti,ab,kw.	1191403
157	((ductal or intraductal or intra-ductal) adj (carcinoma? or hyperplasia?)).ti,ab,kw.	53111
158	DCIS.ti,ab,kw.	19836
159	(lobul* carcinoma? adj2 “in situ”).ti,ab,kw.	3079
160	LCIS.ti,ab,kw.	2085
161	(paget* and (areola? or breast* or mammary or nipple*)).ti,ab,kw.	3671
162	or/154-161 [BREAST CANCER]	1448269
163	Receptor, ErbB-2/	122584
164	(ERBB2 protein adj1 human).mp.	16841
165	(ErbB2 or “ErbB 2” or HER2* or “HER 2*” or “c-ErbB2” or “C-ErbB 2”).ti,ab,kw.	193810
166	((oncoprotein* or onco-protein* or protein* or receptor*) adj1 (neu or neuregulin)).ti,ab,kw.	3315
167	CD340.ti,ab,kw.	52
168	(“p185(c-neu)” or p185erbB).ti,ab,kw.	90
169	(neu protooncogene or neu proto-oncogene).ti,ab,kw.	458
170	NGL.ti,ab,kw.	2098
171	metastatic lymph node gene 19.ti,ab,kw.	0
172	(MLN19 or MLN 19).ti,ab,kw.	2
173	(human adj1 “epidermal growth factor receptor 2”).ti,ab,kw.	34762
174	ErbB Receptors/	176577
175	or/163-174	384135

176	162 and 175 [HER2 BREAST CANCER]	174774
177	exp Receptors, Estrogen/	180102
178	estrogen receptor?.ti,ab,kw.	155935
179	oestrogen receptor?.ti,ab,kw.	16617
180	ERalpha*.ti,ab,kw.	31182
181	ERbeta.ti,ab,kw.	15889
182	ER positive.ti,ab,kw.	22771
183	“luminal a”.ti,ab,kw.	11821
184	“luminal b”.ti,ab,kw.	9962
185	(luminal adj2 subtype*).ti,ab,kw.	7448
186	hormone receptor?.ti,ab,kw.	101057
187	HR positive.ti,ab,kw.	5875
188	progesterone receptor?.ti,ab,kw.	63122
189	progestin receptor?.ti,ab,kw.	1942
190	PR positive.ti,ab,kw.	4757
191	or/177-190	358109
192	162 and 191 [HR BREAST CANCER]	187656
193	exp Breast Neoplasms/sc [secondary/metastatic]	5326
194	(advanced adj3 ((breast$1 or mamma or mammary) adj3 (adenocarcinoma* or cancer* or carcinoma* or neoplasm* or tumour* or tumor*))).ti,ab,kw.	45753
195	(metastatic* adj3 ((breast$1 or mamma or mammary) adj3 (adenocarcinoma* or cancer* or carcinoma* or neoplasm* or tumour* or tumor*))).ti,ab,kw.	84033
196	(metastas* adj3 ((breast$1 or mamma or mammary) adj3 (adenocarcinoma* or cancer* or carcinoma* or neoplasm* or tumour* or tumor*))).ti,ab,kw.	44117
197	(progressive adj3 ((breast$1 or mamma or mammary) adj3 (adenocarcinoma* or cancer* or carcinoma* or neoplasm* or tumour* or tumor*))).ti,ab,kw.	710
198	(secondary adj3 ((breast$1 or mamma or mammary) adj3 (adenocarcinoma* or cancer* or carcinoma* or neoplasm* or tumour* or tumor*))).ti,ab,kw.	3408
199	(terminal adj3 ((breast$1 or mamma or mammary) adj3 (adenocarcinoma* or cancer* or carcinoma* or neoplasm* or tumour* or tumor*))).ti,ab,kw.	180

200	or/193-199 [METASTATIC BREAST CANCER]	158833
201	176 or 192 or 200 [BREAST CANCERS OF INTEREST]	384696
202	exp Infant/ not exp Adult/	1958469
203	exp Child/ not exp Adult/	3994275
204	Adolescent/ not exp Adult/	1522844
205	201 not (202 or 203 or 204) [INFANT-, CHILD-, ADOLESCENT-ONLY REMOVED]	383813
206	Palbociclib.mp.	13276
207	(palbociclib or ibrance$2 or palbociclib or “pd 0332991” or “pd 0332991” or pd332991or pd332991 or “pf 00080665” or pf00080665).ti,ab,kw.	8960
208	ribociclib.mp.	5984
209	(ribociclib or kisqali$2 or “lee 011” or lee 011a or lee 011bba or lee 11 or lee 11a or lee 11bba or lee011 or lee011a or lee011bba or lee11 or lee11a or lee11bba).ti,ab,kw.	4124
210	abemaciclib.mp.	6507
211	(abemaciclib or bemaciclib$2 or ly 2835219 or ly2835219 or verzenio$2).ti,ab,kw.	4441
212	Cyclin-Dependent Kinase Inhibitor Proteins/	9984
213	cyclin-dependent kinase inhibitor?.ti,ab,kw.	16741
214	(CDKI or CDKIs).ti,ab,kw.	1653
215	CKI protein?.ti,ab,kw.	47
216	(CIP-KIP adj2 protein?).ti,ab,kw.	90
217	Cyclin-Dependent Kinases/	24358
218	Cyclin-Dependent Kinase 4/	22740
219	Cyclin-Dependent Kinase 6/	13868
220	(Cyclin-Dependent Kinase 4 or Cyclin-Dependent Kinase 6).ti,ab,kw.	7262
221	(CDK4* or CDK 4* or CDK6* or CDK 6*).ti,ab,kw.	38438
222	(Cell Division Protein Kinase 4 or PSK-J3 Kinase or PSKJ3 Kinase or p34PSK-J3 Kinase or p34PSKJ3 Kinase).ti,ab,kw.	6
223	(Cell Division Protein Kinase 6 or PLSTIRE Protein).ti,ab,kw.	12
224	or/206-223 [FIRST LINE DRUGS]	92744

225	205 and 224 [BREAST CANCERS OF INTEREST - FIRST LINE DRUGS]	15958
226	225 use coch,cctr [COCHRANE RECORDS]	1658
227	75 or 153 or 226 [ALL DATABASES]	7212
228	limit 75 to dt=”20250601-20251231” [MEDLINE update results - June 2025 - Current]	202
229	limit 153 to dc=”20250601-20251231” [Embase update results - June 2025 - Current]	644
230	226 and (2025$ not (202501$ or 202502$ or 202503$ or 202504$)).up. [CENTRAL & CDSR update results - May 2025 - Current]	175
231	228 or 229 or 230	1021
232	limit 231 to yr=”2025 -Current” [Results published in 2025 and later]	903
233	remove duplicates from 232	698
234	“10 1016 j esmoop 2025 105570”.do.	2
235	233 and 234	1

EndNote deduplication process: 33 duplicates removed

Figure S1. PRISMA flow diagrams

August 2024 Update

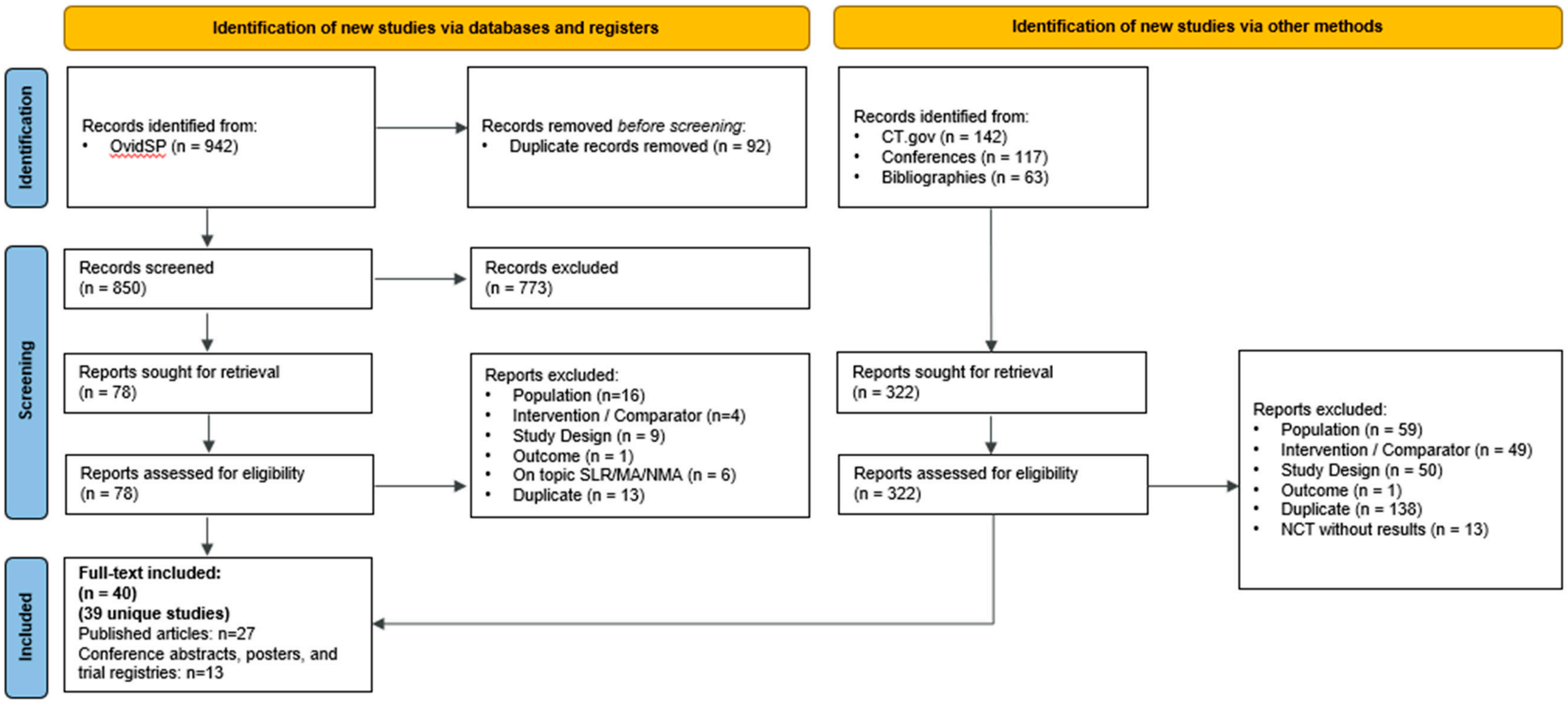

a Studies were excluded from the analysis if they had sample sizes less than 100 patients and did not specify the line of therapy or type of CDK4/6i assessed.

MA = meta-analysis; NCT = National Clinical Trial; PRISMA = Preferred Reporting Items for Systematic Reviews and Meta-Analyses; SLR = systematic literature review

From: Page MJ, McKenzie JE, Bossuyt PM, Boutron I, Hoffmann TC, Mulrow CD, et al. The PRISMA 2020 statement: an updated guideline for reporting systematic reviews. BMJ 2021;372:n71. doi: 10.1136/bmj.n71.

September 2024 Update

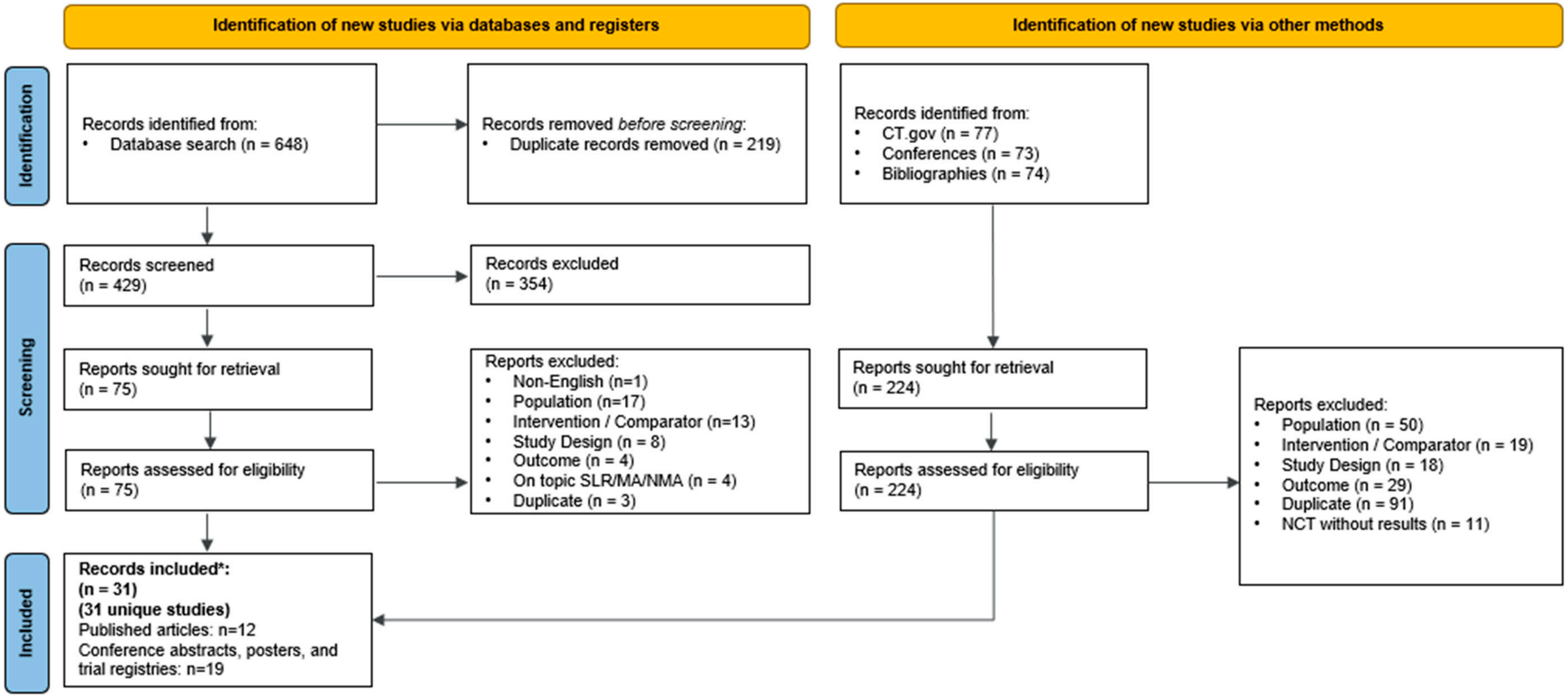

a Studies were excluded from the analysis if they had sample sizes less than 100 patients and did not specify the line of therapy or type of CDK4/6i assessed.

MA = meta-analysis; NCT = National Clinical Trial; PRISMA = Preferred Reporting Items for Systematic Reviews and Meta-Analyses; SLR = systematic literature review

From: Page MJ, McKenzie JE, Bossuyt PM, Boutron I, Hoffmann TC, Mulrow CD, et al. The PRISMA 2020 statement: an updated guideline for reporting systematic reviews. BMJ 2021;372:n71. doi: 10.1136/bmj.n71.

December 2024 Update

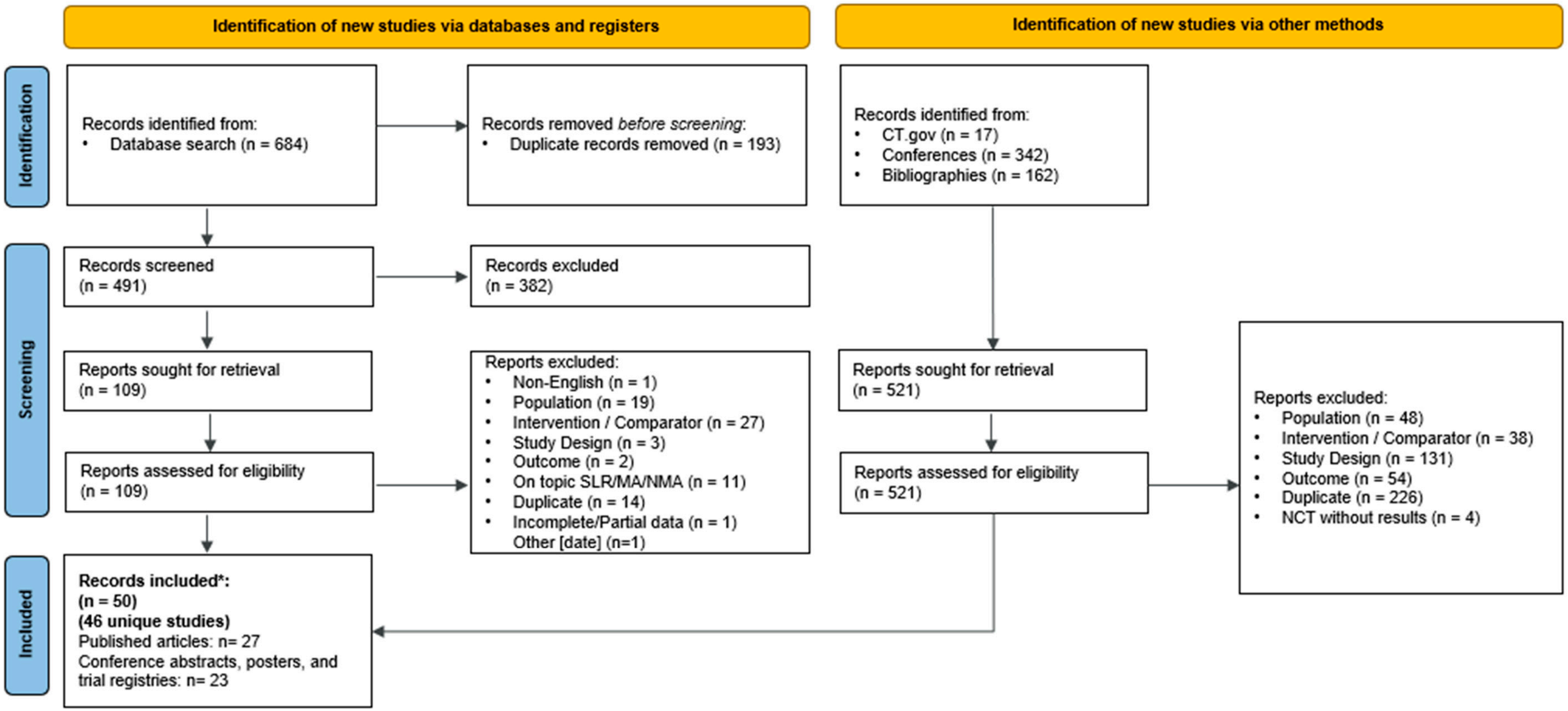

a Studies were excluded from the analysis if they had sample sizes less than 100 patients and did not specify the line of therapy or type of CDK4/6i assessed.

MA = meta-analysis; NCT = National Clinical Trial; PRISMA = Preferred Reporting Items for Systematic Reviews and Meta-Analyses; SLR = systematic literature review

From: Page MJ, McKenzie JE, Bossuyt PM, Boutron I, Hoffmann TC, Mulrow CD, et al. The PRISMA 2020 statement: an updated guideline for reporting systematic reviews. BMJ 2021;372:n71. doi: 10.1136/bmj.n71.

June 2025 Update

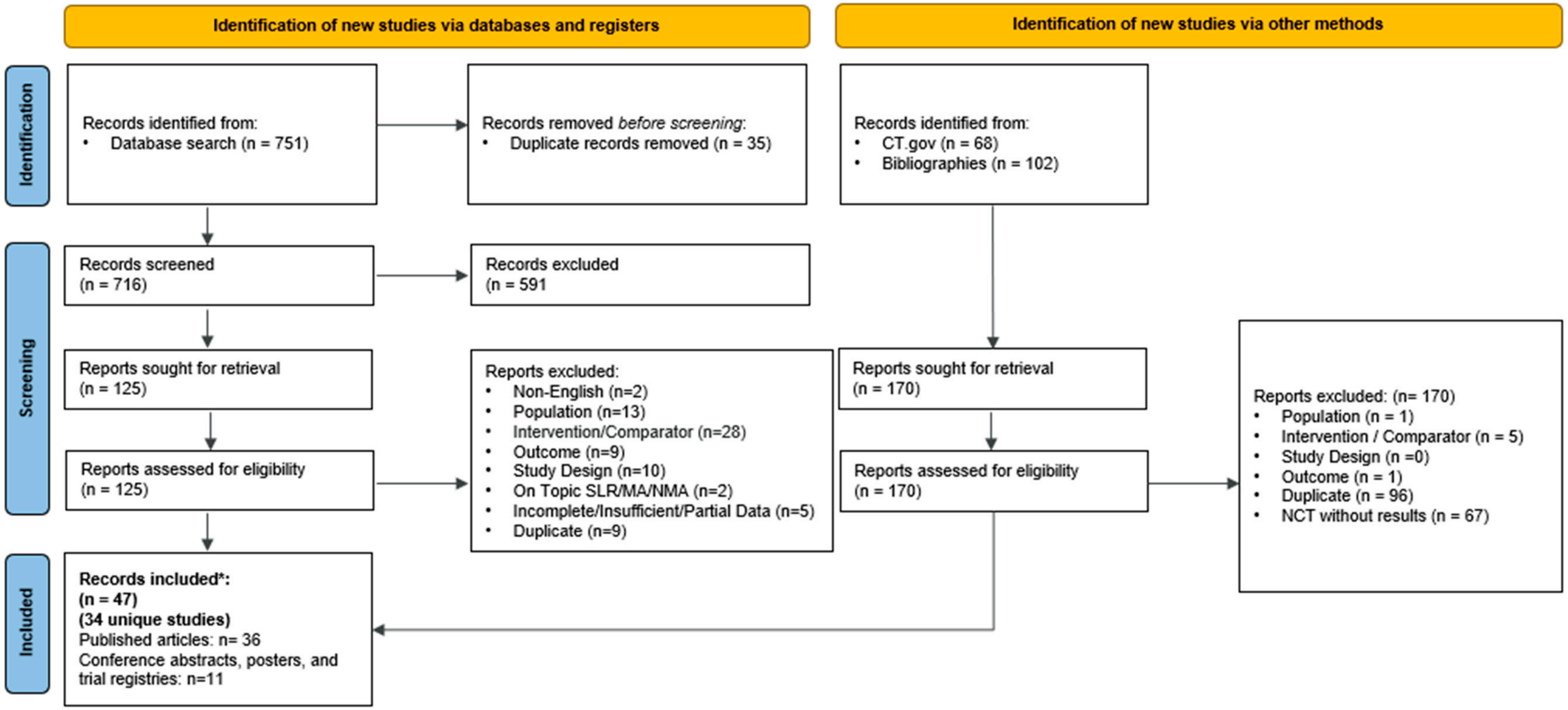

[a] Studies were excluded from the analysis if they had sample sizes less than 100 patients and did not specify the line of therapy or type of CDK4/6i assessed.

MA = meta-analysis; NCT = National Clinical Trial; PRISMA = Preferred Reporting Items for Systematic Reviews and Meta-Analyses; SLR = systematic literature review

From: Page MJ, McKenzie JE, Bossuyt PM, Boutron I, Hoffmann TC, Mulrow CD, et al. The PRISMA 2020 statement: an updated guideline for reporting systematic reviews. BMJ 2021;372:n71. doi: 10.1136/bmj.n71.

September 2025 Update

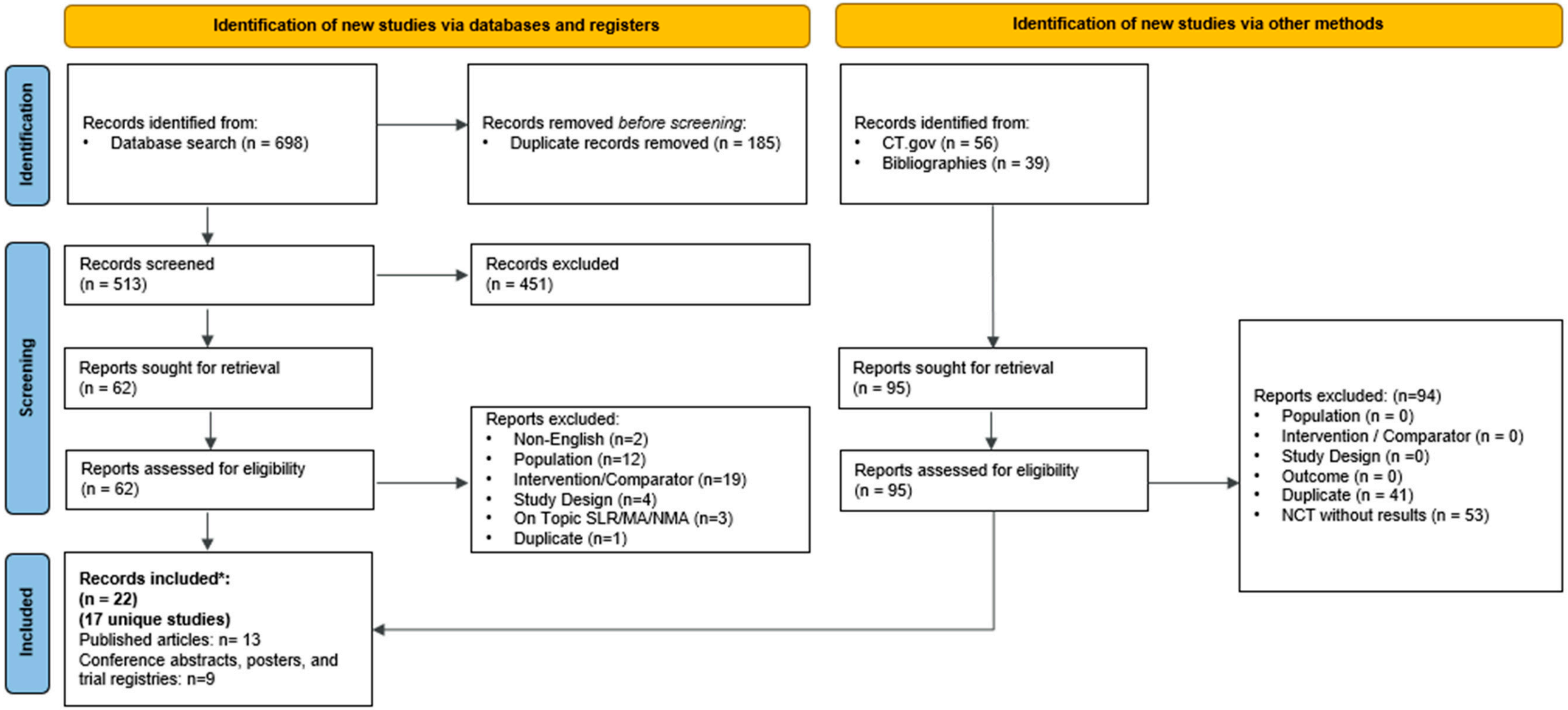

[a] Studies were excluded from the analysis if they had sample sizes less than 100 patients and did not specify the line of therapy or type of CDK4/6i assessed.

MA = meta-analysis; NCT = National Clinical Trial; PRISMA = Preferred Reporting Items for Systematic Reviews and Meta-Analyses; SLR = systematic literature review

From: Page MJ, McKenzie JE, Bossuyt PM, Boutron I, Hoffmann TC, Mulrow CD, et al. The PRISMA 2020 statement: an updated guideline for reporting systematic reviews. BMJ 2021;372:n71. doi: 10.1136/bmj.n71.

Table S2. List of included studies

Study name<br>Author	Year	Country/<br>Region	Full text or<br>abstract	Citation
**NR**<br>**Buller**	2023	United States	Full text	Buller, W., Pallan, L., Chu, T., & Khoja, L. (2023). CDK4/6 inhibitors in metastatic breast cancer, a comparison of toxicity and efficacy across agents in a real-world dataset. *J Oncol Pharm Pract*, *29*(8), 1825-1835. https://doi.org/10.1177/10781552231163121
**NR**<br>**Cejuela**	2023	Spain	Full text	Cejuela, M., Gil-Torralvo, A., Castilla, M. Á., Domínguez-Cejudo, M. Á., Falcón, A., Benavent, M., Molina-Pinelo, S., Ruiz-Borrego, M., & Salvador Bofill, J. (2023). Abemaciclib, Palbociclib, and Ribociclib in Real-World Data: A Direct Comparison of First-Line Treatment for Endocrine-Receptor-Positive Metastatic Breast Cancer. *International Journal of Molecular Sciences*, *24*(10), 8488. https://doi.org/10.3390/ijms24108488
**NR**<br>**Gullick**	2023	United Kingdom	Abstract	Gullick, G., Owen, C., Cook, S., Helbrow, J., Squires, R., Reed, H. M., et al. (2023). 422P UK real-world data (RWD) of cyclin-dependent kinase 4/6 inhibitor (CDK4/6i) use in metastatic breast cancer (MBC). *Annals of Oncology*, *34*, S360. https://doi.org/10.1016/j.annonc.2023.09.598
NR<br>Kahraman	2023	Japan	Full text	Kahraman, S., Erul, E., Seyyar, M., Gumusay, O., Bayram, E., Demirel, B. C., et al. (2023). Treatment efficacy of ribociclib or palbociclib plus letrozole in hormone receptor-positive/HER2-negative metastatic breast cancer. *Future Oncol*, *19*(10), 727-736. https://doi.org/10.2217/fon-2022-1287
**NR**<br>**Lenza**	2023	Spain	Abstract	Lenza, I. C., Valenti, E. L., Alonso, M. G., Ambite, R. Á., Sosa, M. H., Quevedo, R. E. A., et al. (2023). 467P Initial results from the Canarian registry of luminal breast cancer patients treated with first-line CDK 4/6 inhibitors. *Annals of Oncology*, *34*, S377-S378. https://doi.org/10.1016/j.annonc.2023.09.643
**GOIRC-04-2019**<br>**Moscetti**	2023	Europe (multiple countries)	Abstract	Moscetti, L., Canino, F., Natalizio, S., Sperduti, I., Barbieri, E., Piacentini, F., et al. (2024). Abstract PO1-05-02: CDK4/6 inhibitors in advanced breast cancer (aBC). Preliminary results of the CDK4/6i choice and sequence of treatments in a series of 174 patients. GOIRC-04-2019 retro/prospective observational study. *Cancer Research*,

Study name<br>Author	Year	Country/<br>Region	Full text or abstract	Citation
				84(9_Supplement), PO1-05-02-PO01-05-02. https://doi.org/10.1158/1538-7445.SABCS23-PO1-05-02
NR<br>Tang	2023	United Kingdom	Abstract	Tang, H. K. C., De Souza, K., Ahmad, O., Shafiq, T., Khan, S., Anand, A., et al. (2023). Palbociclib or Ribociclib with Aromatase Inhibitor in Post-menopausal Women with ER+/HER2- Advanced Breast Cancer? Real-world Overall Survival Evidence. *Clinical Oncology*, *35*(6), e420. https://doi.org/10.1016/j.clon.2023.02.040
**NR**<br>**Tang**	2023	United Kingdom	Full text	Tang, H., Yeo, D., De Souza, K., Ahmad, O., Shafiq, T., Ofor, O., et al. (2023). Clinical Impact of CDK4/6 Inhibitors in De Novo or PR− or Very Elderly Post-Menopausal ER+/HER2− Advanced Breast Cancers. *Cancers*, *15*(21), 5164. https://doi.org/10.3390/cancers15215164
OPAL;<br>NCT03417115<br>Thill	2023	Germany	Abstract	Thill, M., Zahn, M.-O., Welt, A., Nusch, A., Zaiss, M., Engelken, K et al. (2024). Abstract PO1-04-12: Palbociclib versus ribociclib in first-line treatment of patients with hormone-receptor positive HER2 negative advanced breast cancer – real world outcome data from the German registry platform OPAL. *Cancer Research*, *84*(9_Supplement), PO1-04-12-PO01-04-12. https://doi.org/10.1158/1538-7445.SABCS23-PO1-04-12
**NR**<br>**Weipert**	2023	Spain	Abstract	Weipert, C., Wander, S., Davis, A., Bucheit, L., Saha, J., Liao, J., et al. (2024). Abstract PO4-18-02: Real-world (RW) utilization and patient outcomes across three CDK4/6 inhibitors in metastatic breast cancer (mBC). *Cancer Research*, *84*(9_Supplement), PO4-18-02-PO14-18-02. https://doi.org/10.1158/1538-7445.SABCS23-PO4-18-02
**NR**<br>**Betiol**	2024	Global (multiple regions)	Abstract	Betiol, J. C., Bueno, B., & Exman, P. (2025). Abstract P3-08-05: Cyclin inhibitors for breast cancer: A comparative real world data analysis. *Clinical Cancer Research*, *31*(12_Supplement), P3-08-05-P03-08-05. https://doi.org/10.1158/1557-3265.SABCS24-P3-08-05
**NR**<br>**Brufsky**	2024	United States	Abstract	Brufsky, A., Finn, R. S., Metzger, O., Goncalves, R., Huang-Bartlett, C., Sreenivasan, et al. (2025). Abstract P2-09-30: Real-world comparative efficacy of CDK4/6 inhibitors in first-line treatment of HR+/HER2- metastatic breast cancer. *Clinical Cancer Research*, *31*(12_Supplement),

Study name<br>Author	Year	Country/<br>Region	Full text or abstract	Citation
				P2-09-30-P02-09-30. https://doi.org/10.1158/1557-3265.SABCS24-P2-09-30
YOUNGBC-28<br>Chen	2024	China	Full text	Chen, Y., Xie, Y., Sang, D., Xie, N., Han, X., Zhao, Y et al. (2024). Real-world comparison of palbociclib, abemaciclib, and dalpiciclib as first-line treatments for Chinese HR+/HER2−metastatic breast cancer patients: a multicenter study (YOUNGBC-28). *Therapeutic Advances in Medical Oncology*, *16*, 17588359241302018. https://doi.org/10.1177/17588359241302018
NR<br>Coutinho-Almeida	2024	Portugal	Full text	Coutinho-Almeida, J., Silva, A. S., Redondo, P., Rodrigues, P. P., & Ferreira, A. (2024). CDK4/6 inhibitors and endocrine therapy in the treatment of metastatic breast cancer: A real-world and propensity score-adjusted comparison. *Cancer Treat Res Commun*, *40*, 100818. https://doi.org/10.1016/j.ctarc.2024.100818
CEPRA<br>Dajsakdipon	2024	Thailand	Full text	Dajsakdipon, T., Susiriwatananont, T., Wongkraisri, C., Ithimakin, S., Parinyanitikul, N., Supavavej, A., et al. (2024). Comparative effectiveness analysis of survival with first-line palbociclib or ribociclib plus AI in HR + /HER2-advanced breast cancer (CEPRA study): preliminary analysis of real-world data from Thailand. *BMC Cancer*, *24*(1), 1018. https://doi.org/10.1186/s12885-024-12765-x
**NR**<br>**Duchnowska**	2024	Poland	Abstract	Duchnowska, R., Soter, K., Smok-Kalwat, J., Grela-Wojewoda, A., Winsko-Szczęsnowicz, K., Pogoda, K., et al., (2024). Real-world treatment outcomes in patients with HR+ HER2- advanced breast cancer treated with CDK4/6 inhibitors and endocrine therapy. *Journal of Clinical Oncology*, *42*(16_suppl), 1067-1067. https://doi.org/10.1200/JCO.2024.42.16_suppl.1067
**NR**<br>**Gehrchen**	2024	Denmark	Full text	Gehrchen, M. L., Berg, T., Garly, R., Jensen, M.-B., Eßer-Naumann, S., Rønlev, J. D., et al. (2024). Real-world effectiveness of CDK 4/6 inhibitors in estrogen-positive metastatic breast cancer. *BJC Reports*, *2*(1), 44. https://doi.org/10.1038/s44276-024-00070-w
NR<br>Karhan	2024	Turkey	Full text	Karhan, O., İleri, S., Urakçı, Z., Arvas, H., Kılıç, D. K., Sezgin, Y., et al. (2024). Concomitant Use of Proton Pump Inhibitors and CDK4/6 Inhibitors in Metastatic Hormone-Positive

Study name<br>Author	Year	Country/<br>Region	Full text or abstract	Citation
				Breast Cancer: A Real-World Cohort Study. *Oncology*, *103*(6), 498-507. https://doi.org/10.1159/000542693
NR<br>Oner	2024	Turkey	Full text	Öner, İ., Türkel, A., Anık, H., Arslan, Ü. Y., & Karaçin, C. (2024). Evaluation of CDK4/6 inhibitors in first-line in symptomatic and asymptomatic patients with metastatic breast cancer. *Future Oncology*, *20*(40), 3443-3450. https://doi.org/10.1080/14796694.2024.2432850
NR<br>Orlova	2024	Russia	Abstract	Orlova, R., Avramenko, I., Androsova, A., Topuzov, E., Belyak, N. P., Kutukova, S., et al. (2024). The analysis of prescribing CDK4/6 inhibitors for patients with breast cancer in real clinical practice. *Journal of Clinical Oncology*, *42*(16_suppl), e13082-e13082. https://doi.org/10.1200/JCO.2024.42.16_suppl.e13082
**NR**<br>**Plavetic**	2024	Croatia	Abstract	Plavetic, D. N., Čular, K., Gudelj, D., Kaceljak, K., Križić, M., Popovic, M., et al. (2024). Real-world comparison of the efficacy of three CDK4/6 inhibitors (CDK4/6i) in the first-line treatment of endocrine-sensitive advanced breast cancer (aBC): Single institution experience. *Journal of Clinical Oncology*, *42*(16_suppl), e13080-e13080. https://doi.org/10.1200/JCO.2024.42.16_suppl.e13080
**P-VERIFY; NCT06495164**<br>**Rugo**	2024	United States	Abstract	Rugo, H. S., Layman, R. M., Lynce, F., Liu, X., Li, B., McRoy, L., et al. (2024). Abstract PS2-03: Comparative overall survival of CDK4/6is plus an aromatase inhibitor (AI) in HR+/HER2- MBC in the US real-world setting. *Clinical Cancer Research*, *31*(12_Supplement), PS2-03-PS02-03. https://doi.org/10.1158/1557-3265.SABCS24-PS2-03
**NR**<br>**Skocilic**	2024	Croatia	Full text	Skocilic, I., Golcic, M., Bukovica Petrc, A., Kolak, M., Kolovrat, D., Ropac, et al. (2024). Real-World Data with CDK4/6 Inhibitors - A Single Center Experience from Croatia. *Journal of Personalized Medicine*, *14*(9), 895. https://doi.org/10.3390/jpm14090895
OPAL<br>Thill	2024	Germany	Full text	Thill, M., Zahn, M.-O., Welt, A., Nusch, A., Zaiss, M., Engelken, K., et al. (2025). Head-to-head comparison of palbociclib and ribociclib in first-line treatment of HR-positive/HER2-negative metastatic breast cancer with real-world data from the OPAL registry. *International Journal of Cancer*, *156*(9), 1770-1782. https://doi.org/https://doi.org/10.1002/ijc.35296

Study name<br>Author	Year	Country/<br>Region	Full text or<br>abstract	Citation
NR<br>Tsareva	2024	Russia	Abstract	Tsareva, E., Stativko, O., Shangina, I., Khachaturian, E., Antonova, T., Pokataev, I., & Galkin, V. N. (2024). 56P Palbociclib versus ribociclib in first-line therapy of patients with metastatic breast cancer: Direct comparison in real-world practice. *Annals of Oncology*, *35*, S1425. https://doi.org/10.1016/j.annonc.2024.10.076
**PALMARES-2;<br>NCT06805812<br>Vernieri**	2024	Italy	Abstract	Vernieri, C., Provenzano, L., Giuliano, M., Rizzo, G., Toss, A., Piras, M., et al. (2024). Comparison of antitumor efficacy of first-line palbociclib, ribociclib, or abemaciclib in patients with HR+/HER2- aBC: Results of the multicenter, real-world, Italian study PALMARES-2. *Journal of Clinical Oncology*, *42*(16_suppl), 1014-1014. https://doi.org/10.1200/JCO.2024.42.16_suppl.1014
PALMARES-2;<br>NCT06805812<br>Vernieri	2024	Italy	Abstract	Vernieri, C., Ligorio, F., Provenzano, L., Fotia, G., Giuliano, M., Rizzo, G., et al. (2025). Abstract P2-09-26: Real world progression free survival in elderly vs. younger HR+/HER2- advanced breast cancer patients treated with first-line endocrine therapy plus CDK4/6i: a sub-analysis of the multicenter, PALMARES-2 study. *Clinical Cancer Research*, *31*(12_Supplement), P2-09-26-P02-09-26. https://doi.org/10.1158/1557-3265.SABCS24-P2-09-26
TOG study<br>Yildirim	2024	Turkey	Full text	Yildirim, H. C., Kapar, C., Koksal, B., Seyyar, M., Sanci, P. C., Guliyev, M., et al., (2025). Efficacy of first-line CDK 4-6 inhibitors in premenopausal patients with metastatic breast cancer and the effect of dose reduction due to treatment-related neutropenia on efficacy: a Turkish Oncology Group (TOG) study. *Journal of Chemotherapy*, *37*(1), 69-75. https://doi.org/10.1080/1120009X.2024.2330835
NR<br>Yildirim	2024	Turkey	Full text	Yildirim, H. C., Buyukkor, M., Kavgaci, G., Celik, B., Yucel, K. B., Dursun, B., et al. (2024). The impact of human epidermal growth factor receptor-2 (low) status on the efficacy of first line cyclin-dependent kinase 4/6 inhibitors in advanced breast cancer. *Medicine (Baltimore)*, *103*(30), e38828. https://doi.org/10.1097/md.0000000000038828
NR<br>Guliyev	2025	Turkey	Full text	Guliyev, M., Güren, A. K., Özge, E., Çolak, R., Majidova, N., Alkan Şen, G., et al. (2025). The Impact of Progesterone

Study name Author	Year	Country/ Region	Full text or abstract	Citation
				Receptor Status on Survival Outcomes in Metastatic Breast Cancer Patients Treated with First-Line CDK4/6 Inhibitors. *Cancers*, *17*(4), 693. https://doi.org/10.3390/cancers17040693
NR<br>Inci	2025	Turkey	Full text	İnci, B. K., Tolunay, P. K., Karabuğa, B., Topkaç, A., Öner, İ., Irkkan, Ç., et al. (2025). Histology-based survival outcomes in breast cancer patients treated with CDK4/6 inhibitor. *Annals of Clinical and Analytical Medicine*, *15*(2), 90-93. https://doi.org/10.4328/ACAM.22317
**PALMARES-2; NCT06805812 Provenzano**	2025	Italy	Full text	Provenzano, L., Dieci, M. V., Curigliano, G., Giuliano, M., Botticelli, A., Lambertini, M., et al. (2025). Real-world effectiveness comparison of first-line palbociclib, ribociclib or abemaciclib plus endocrine therapy in advanced HR-positive/HER2-negative BC patients: results from the multicenter PALMARES-2 study. *Annals of Oncology*, *36*(7), 762-774. https://doi.org/10.1016/j.annonc.2025.03.023
**P-VERIFY; NCT06495164 Rugo**	2025	United States	Full text	Rugo, H. S., Layman, R. M., Lynce, F., Liu, X., Li, B., McRoy, L., et al. (2025a). Comparative overall survival of CDK4/6 inhibitors plus an aromatase inhibitor in HR+/HER2- metastatic breast cancer in the US real-world setting. *ESMO Open*, *10*(1). https://doi.org/10.1016/j.esmoop.2024.104103
**CDK-PREDICT study Tolosa**	2025	Spain	Full text	Tolosa, P., Pascual, T., Martínez-Saez, O., Hernando, C., Servitja, S., Fernández Abad, M., et al. (2025). Efficacy outcomes of CDK4/6 inhibitors in combination with endocrine therapy treatment in hormone receptor-positive/HER2-negative advanced breast cancer according to PAM50 intrinsic subtype: Primary results of SOLTI-1801 CDK-PREDICT study. *European Journal of Cancer*, *217*. https://doi.org/10.1016/j.ejca.2024.115219
**PALMARES-2; NCT06805812 Vernieri**	2025	Italy	Abstract	Vernieri, C., Dieci, M. V., Curigliano, G., Giuliano, M., Botticelli, A., Lambertini, M., et al. (2025). 309P Prognostic and predictive value of lobular tumor histology in advanced HR+/HER2- breast cancer treated with ET plus CDK4/6i: A sub-analysis of the PALMARES-2 study. *ESMO Open*, *10*. https://doi.org/10.1016/j.esmoop.2025.104881

Study name<br>Author	Year	Country/<br>Region	Full text or abstract	Citation
NR<br>Yoshinami	2025	Japan	Full text	Yoshinami, T., Takano, Y., Ozaki, Y., Kajiwara, Y., Yamamoto, M., Watanabe, K.-i., et al. (2025). Real-world evidence from Japan regarding survival outcomes and treatment sequence in patients receiving CDK4/6 inhibitor plus endocrine therapy as first- or second-line treatment for hormone receptor–positive, HER2-negative advanced or metastatic breast cancer. *Breast Cancer*, *32*(4), 841-856. https://doi.org/10.1007/s12282-025-01713-7
**PALMARES-2; NCT06805812<br>Vernieri**	2025	Italy	Abstract	Vernieri, C., Dieci, M. V., Caputo, R., Vigneri, P., Giuliano, M., Curigliano, G., et al. (2025). Effectiveness comparison of palbociclib, ribociclib and abemaciclib in patients with HR+/HER2- aBC: Updated results from the real-world, Italian study PALMARES-2. *Journal of Clinical Oncology*, *43*(16_suppl), 1074-1074. https://doi.org/10.1200/JCO.2025.43.16_suppl.1074
NR<br>Trigueros	2025	Costa Rica	Abstract	Marin Trigueros, D. M., & Landaverde, D. U. (2025). Real-world data on the effectiveness and toxicity of CDK 4/6 inhibitors combined with hormonal therapy in patients with metastatic hormone receptor-positive and HER2-negative breast cancer in first- and second-line treatments within the Costa Rican health care system. *Journal of Clinical Oncology*, *43*(16_suppl), e13053-e13053. https://doi.org/10.1200/JCO.2025.43.16_suppl.e13053
**P-VERIFY; NCT06495164<br>Rugo**	2025	United States	Full text	Rugo, H. S., Layman, R. M., Lynce, F., Liu, X., Li, B., McRoy, L., Cohen, et al. (2025b). Real-world progression-free survival of CDK4/6 inhibitors plus an aromatase inhibitor in HR-positive/HER2-negative metastatic breast cancer in United States routine clinical practice. *ESMO Open*, *10*(9). https://doi.org/10.1016/j.esmoop.2025.105570

Note: bolding indicates studies that compared all three CDK4/6i

File S1. Quality assessments

Newcastle-Ottawa Scale

Newcastle-Ottawa
Scale.xlsx

Summary of Newcastle-Ottawa Scale Scores

Study Name; Reference	Score
3810-Kahraman-2023; Japan	6
4112-Cejuela-2023; Spain	7
4132-Buller-2023; United States	5
4506-Tang-2023; United Kingdom	7
TOG study; 4986-Yildirim-2024; Turkey	6
4987-Yildirim-2024; Turkey	4
5630-Coutinho-Almeida-2024; Portugal	8
5893-Skocilic-2024; Croatia	7
CEPRA; 6138-Dajsakdipon-2024; Thailand	6
OPAL registry; 6334-Thill-2024; Germany	9
6431-Oner-2024; Turkey	8
6522-Karhan-2024; Turkey	8
6590-Gehrchen-2024; Denmark	8
YOUNGBC-28; 6637-Chen-2024; China	9
309-Guliyev-2025; Turkey	9
342-Inci-2025; Turkey	8
PALMARES-2; 540-Provenzano-2025; Italy	9
P-VERIFY; 563-Rugo-2025; United States	8
CDK-PREDICT; 633-Tolosa-2025; Spain	8
694-Yoshinami-2025; Japan	8
P-VERIFY; 1132-Rugo-2025; United States	8

ISPOR Questionnaire

ISPOR Questionnaire.xlsx

Summary of ISPOR Questionnaire Credibility

Study Name; Reference	Overall Credibility
3810-Kahraman-2023; Japan	Sufficient
4112-Cejuela-2023; Spain	Sufficient
4132-Buller-2023; United States	Sufficient
4506-Tang-2023; United Kingdom	Sufficient
TOG study;4986-Yildirim-2024	Sufficient
4987-Yildirim-2024; Turkey	Sufficient
5630-Coutinho-Almeida-2024; Portugal	Sufficient
5893-Skocilic-2024; Croatia	Sufficient
CEPRA; 6138-Dajsakdipon-2024; Thailand	Sufficient
OPAL registry; 6334-Thill-2024; Germany	Sufficient
6431-Oner-2024; Turkey	Sufficient
6522-Karhan-2024; Turkey	Sufficient
6590-Gehrchen-2024; Denmark	Sufficient
YOUNGBC-28; 6637-Chen-2024; China	Sufficient
309-Guliyev-2025; Turkey	Sufficient
342-Inci-2025; Turkey	Sufficient
PALMARES-2; 540-Provenzano-2025; Italy	Sufficient
P-VERIFY; 563-Rugo-2025; United States	Sufficient
CDK-PREDICT; 633-Tolosa-2025; Spain	Sufficient
694-Yoshinami-2025; Japan	Sufficient
P-VERIFY; 1132-Rugo-2025; United States	Sufficient

ESMO-GROW Checklist

ESMO GROW.xlsx

Summary of ESMO-GROW Questionnaire

Study Name; Reference	Overall Credibility
3810-Kahraman-2023; Japan	Sufficient
4112-Cejuela-2023; Spain	Sufficient
4132-Buller-2023; United States	Sufficient
4506-Tang-2023; United Kingdom	Sufficient
TOG study;4986-Yildirim-2024; Turkey	Sufficient
4987-Yildirim-2024; Turkey	Sufficient
5630-Coutinho-Almeida-2024; Portugal	Sufficient
5893-Skocilic-2024; Croatia	Sufficient
CEPRA; 6138-Dajsakdipon-2024; Thailand	Sufficient
OPAL registry; 6334-Thill-2024; Germany	Sufficient
6431-Oner-2024; Turkey	Sufficient
6522-Karhan-2024; Turkey	Sufficient
6590-Gehrchen-2024; Denmark	Sufficient
YOUNGBC-28; 6637-Chen-2024; China	Sufficient
309-Guliyev-2025; Turkey	Sufficient
342-Inci-2025; Turkey	Sufficient
PALMARES-2; 540-Provenzano-2025; Italy	Sufficient
P-VERIFY; 563-Rugo-2025; United States	Sufficient
CDK-PREDICT; 633-Tolosa-2025; Spain	Sufficient
694-Yoshinami-2025; Japan	Sufficient
P-VERIFY; 1132-Rugo-2025; United States	Sufficient

Table S3. List of subgroups of interest

Metastases (e.g., visceral, bone, liver, etc.)
ET response (e.g., de novo, endocrine-resistant, endocrine-sensitive)
Hormonal status (e.g., HER2 0, HER2 +1, ER/PR strong, ER/PR weak, etc.)
Age (e.g., older adults)
Race/ethnicity (e.g., BIPOC)
ECOG score
Comorbidity (e.g., specific disorders, CCI)/risk factors
Menopausal status

Abbreviations: BIPOC = Black, Indigenous, and People of Color; CCI = Charlson Comorbidity Index; CDK4/6i = cyclin-dependent kinase 4/6 inhibitors; ECOG = Eastern Cooperative Oncology Group; ET = endocrine therapy; ER = endocrine receptor; HER = Human Epidermal Growth Factor Receptor; PR = progesterone receptor.

Progression-free survival and overall survival for first-line in comparative RWE studies (studies with manuscript data available)

Table S4. Progression-free survival and overall survival for first-line palbociclib in comparative RWE studies versus ribociclib (overall population; studies with manuscript data available)

Study Name; Reference	Treatment	Subgroup	Sample size	Follow-up Time	Starting dose for CDK4/6i , n (%)	PFS			OS		
						Median (95% CI), months	HR (95% CI); P value	At latest timepoint , n (%)	Median (95% CI), months	HR (95% CI); P value	At latest timepoin t, n (%)
309-Guliyev-2025; Turkey	Ribociclib + ET	All patients	245	Median 25.2 (range: 1.5–82.6) months	NR	NR	Palbociclib better: 1.26 (0.91-1.76); P=0.170	NR	NR	Palbociclib better 1.19 (0.74-1.91); P=0.479	NR
	Palbociclib + ET	All patients	106		NR	NR		NR	NR		NR
342-Inci-2025; Turkey	Ribociclib + ET	All patients	175	Median 30 (95% CI: 25-32) months	NR	NR	Palbociclib better: 1.4 (0.68-1.58); P=0.867	NR	NR	NR	NR
	Palbociclib + ET	All patients	73		NR	NR		NR	NR	NR	NR
CDK-PREDICT study 633-Tolosa-2025; Spain	Palbociclib + ET	All patients	86	Median 38.5 (IQR: 26.5-53.8) months	NR	20.7 (16.6-33.3)	NR	NR	85.8 (47.6-not reached)	NR	NR
	Ribociclib + ET	All patients	82		NR	22.9 (17.2-29.2)	NR	NR	55.4 (42.8-75.1)	NR	NR
3810-Kahraman-2023; Japan	Palbociclib + Letrozole	All patients	272	Median 10.8 (95% CI: 9.9–11.5) months	NR	32.2 (12.4-51.97)	NR; P=0.953	NR	NR	NR; P=0.252	60 months: NR (52)
	Ribociclib + Letrozole	All patients	328		NR	29.4 (13.45-45.42)		NR	NR		60 months: NR (58)
4112-Cejuela-2023; Spain	Palbociclib + ET	All patients	96	Median 75 months	NR	30.03 (NR)	NR; P=0.984	NR	Not reached	NR; P=0.904	NR

Study Name; Reference	Treatment	Subgroup	Sample size	Follow-up Time	Starting dose for CDK4/6i, n (%)	PFS			OS		
						Median (95% CI), months	HR (95% CI); P value	At latest timepoint, n (%)	Median (95% CI), months	HR (95% CI); P value	At latest timepoint, n (%)
	Ribociclib + ET	All patients	54	Median 64.4 months	NR	31.14 (NR)		NR	Not reached		NR
4132-Buller-2023; United States	Palbociclib + ET	All patients	115	NR	NR	23.00 (17.63-28.37)	NR	NR	44.30 (31.16-57.44)	NR	NR
	Ribociclib + ET	All patients	28	NR	NR	31.10 (NR)	NR	NR	NR	NR	NR
4506-Tang-2023; United Kingdom	Palbociclib + ET	All patients	162	Median 49.5 months	NR	27.5 (NR)	NR	5 years: NR (20.88)	49.5 (NR)	NR	5 years: NR (48.54)
	Ribociclib + ET	All patients	46		NR	25.7 (NR)	NR	5 years: NR (32.58)	50.2 (NR)	NR	5 years: NR (42.33)
TOG study; 4986-Yildirim-2024; Turkey	Palbociclib + AI/FUL	All patients	128	Median 21.46 months	NR	26.83 (22.74–30.92)	Ribociclib better: 1.01 (0.71-1.44); P=0.924	NR	NR	NR	NR
	Ribociclib + AI/FUL	All patients	191		NR	29.86 (22.21–37.52)		NR	NR	NR	NR
4987-Yildirim-2024; Turkey	Palbociclib + AI/FUL	All patients	63	Median 20.33 months	NR	NR	Ribociclib better: 1.06 (0.81-1.39); P=0.652	24 months: 38 (60.3)	NR	NR	NR
	Ribociclib + AI/FUL	All patients	97		NR	NR		24 months: 62 (63.9)	NR	NR	NR
5630-Coutinho-Almeida-2024; Portugal	Palbociclib + ET	All patients	NR	NR	NR	NR	Ribociclib better: 0.41 (NR); P≤0.001	NR	NR	Ribociclib better: 0.64 (NR); P=0.12	NR
	Ribociclib + ET	All patients	NR	NR	NR	NR		NR	NR		NR

Study Name; Reference	Treatment	Subgroup	Sample size	Follow-up Time	Starting dose for CDK4/6i, n (%)	PFS			OS		
						Median (95% CI), months	HR (95% CI); P value	At latest timepoint, n (%)	Median (95% CI), months	HR (95% CI); P value	At latest timepoint, n (%)
	Palbociclib + Letrozole	All patients	NR	NR	NR	NR	Ribociclib + letrozole better: 0.63 (NR); P=0.094	NR	NR	Palbociclib + letrozole better: 1.15 (NR); P=0.7	NR
	Ribociclib + Letrozole	All patients	NR	NR	NR	NR		NR	NR		NR
CEPRA; 6138-Dajsakdipon-2024; Thailand[a]	Ribociclib + AI	PSM analysis	OS: 134 PFS: 134	NR	NR	27.9 (21.8–38.3)	Palbociclib better: 0.87 (0.55–1.37); P=0.54	NR	48.7 (36.6- not estimated)	Palbociclib better: 0.51 (0.28–1.04); P=0.06	NR
	Palbociclib + AI	PSM analysis	OS: 49 PFS: 49	NR	NR	31.8 (19.7–57.4)		NR	59.1 (47.2-not estimated)		NR
6431-Oner-2024; Turkey	Ribociclib + ET	All patients	113	Median 25.3 months	600mg; NR	30.9 (22.4–39.3)	NR; P=0.366	NR	NR	NR	NR
	Palbociclib + ET	All patients	80		125mg, NR	24.6 (18.7–30.5)		NR	NR	NR	NR
6590-Gehrchen-2024; Denmark	Palbociclib + ET	All patients	873	Median 55.1 (95%CI: 52.1-58.0) months	NR	32.0 (28.9-35.3)	Ribociclib better: 0.80 (0.68-0.96); P=0.01	24 months: NR (57)	49.7 (44.7-54.1)	NR	NR
	Ribociclib+ ET	All patients	359	Median 47.9 (95%CI: 45.8-49.7) months	NR	42.4 (35.1-52.9)		24 months: NR (66)	54.4 (47.9-NA)	NR	NR

Study Name; Reference	Treatment	Subgroup	Sample size	Follow-up Time	Starting dose for CDK4/6i , n (%)	PFS			OS		
						Median (95% CI), months	HR (95% CI); P value	At latest timepoint , n (%)	Median (95% CI), months	HR (95% CI); P value	At latest timepoint, n (%)
PALMERES-2 540-Provenzano-2025[a]; Italy	Palbociclib + ET	IPTW-adjusted analyses	NR	45.7 (IQR: 28.0-59.6) months	NR	29.4 (25.9-33.3)	Ribociclib better: 0.83 (0.71-0.97); P=0.02	NR	NR	NR	NR
	Ribociclib + ET	IPTW-adjusted analyses	NR	Median 25.2 (IQR: 12.7-44.4) months	NR	37.4 (32-47.2)		NR	NR	NR	NR
PALMARES-2; 5854-Vernieri-2024; Italy	Palbociclib + ET	All patients	750	NR	NR	NR	Ribociclib better: 0.81 (0.65-0.99); P=0.048	NR	NR	NR	NR
	Ribociclib + ET	All patients	676	NR	NR	NR		NR	NR	NR	NR
PALMARES-2 883-Vernieri-2025; Italy	Palbociclib + ET	IPTW	1392	Median 52.4 months	NR	NR	Ribociclib better: 0.88 (0.80-0.97); P=0.02	NR	NR	Ribociclib better: 0.75 (0.64-0.87); P<0.01	NR
	Ribociclib + ET	IPTW	1408	Median 31.8 months	NR	NR		NR	NR		NR
PALMARES-2; SABCS24-038-Vernieri-2024; Italy	Palbociclib + ET	All patients	789	NR	NR	NR	Ribociclib better: 0.82 (0.70-0.96); P=0.012	NR	NR	NR	NR
	Ribociclib + ET	All patients	138	NR	NR	NR		NR	NR	NR	NR
OPAL registry; 6334-Thill-2024; Germany	Palbociclib + ET	after IPTW	387	Until death or up to 5 years	NR	26.7 (23.6-30.7)	Ribociclib similar to palbociclib:	NR	42.4 (38.8-50.3)	Ribociclib better:	NR

Study Name; Reference	Treatment	Subgroup	Sample size	Follow-up Time	Starting dose for CDK4/6i, n (%)	PFS			OS		
						Median (95% CI), months	HR (95% CI); P value	At latest timepoint, n (%)	Median (95% CI), months	HR (95% CI); P value	At latest timepoint, n (%)
	Ribociclib + ET	after IPTW	233	Until death or up to 5 years	NR	27.0 (21.1-30.4)	1.01 (0.80-1.27); NR	NR	49.3 (36.9-NA)	0.96 (0.71-1.28); NR	NR
OPAL; (NCT03417115)<br><br>SABCS23-007-M.Thill-2023; Germany	Palbociclib + ET	IPTW	387	Until death or up to 5 years	NR	26.7 (23.2-30.7)	Palbociclib is similar to ribociclib: 1.01 (0.80-1.26); NR	NR	41.4 (38.8-50.3)	NR	NR
	Ribociclib + ET	IPTW	233	Until death or up to 5 years	NR	27.0 (21.1-30.7)		NR	49.3 (36.9-not reached)	NR	NR
P-VERIFY 563-Rugo-2025[a]; United States	Palbociclib + AI	After sIPTW adjustment	6832	Median 33.0 (IQR: 34.7) months	NR	NR	NR	NR	54.6 (52.6-56.4)	Ribociclib better:<br><br>0.98 (0.87-1.10); P=0.7531	30 months: NR (71.4)
	Ribociclib + AI	After sIPTW adjustment	1274	Median 15.7 (IQR: 20.8) months	NR	NR	NR	NR	59.0 (50.9-66.1)		30 months: NR (72.2)
P-VERIFY 1132-Rugo-2025[a]; United States	Palbociclib + AI	After sIPTW adjustment	6832	Median 33.0 (IQR: 34.7) months	NR	22.7 (21.6-23.8)	Ribociclib better: 0.97 (0.88-1.07); P=0.5755	NR	NR	NR	NR
	Ribociclib + AI	After sIPTW adjustment	1274	Median 15.7 (IQR: 20.8) months	NR	22.9 (21.0-25.6)		NR	NR	NR	NR

Study Name; Reference	Treatment	Subgroup	Sample size	Follow-up Time	Starting dose for CDK4/6i, n (%)	PFS			OS		
						Median (95% CI), months	HR (95% CI); P value	At latest timepoint, n (%)	Median (95% CI), months	HR (95% CI); P value	At latest timepoint, n (%)
P-VERIFY SABS24-102-Rugo-2024[a,b]; United States	Palbociclib + AI	sIPTW	6832	Median 33.0 (IQR: 34.7) months	NR	NR	NR	NR	54.6 (52.6-56.4)	Ribociclib better: 0.98 (0.87-1.10); P = 0.7531	30 months: NR (71.4)
	Ribociclib + AI	sIPTW	1274	Median 15.7 (IQR: 20.8) months	NR	NR	NR	NR	59.0 (50.9-66.1)		30 months: NR (72.2)

Note: The "Study Name; Reference" cells shaded in grey highlight full text records, while non-shaded are abstracts/manuscripts of the parent study.

[a] Record also reports unadjusted data.

[b] Different data reported in the poster and the abstract.

Abbreviations: AI = aromatase inhibitor; CDK4/6i = cyclin-dependent kinase 4/6 inhibitors; CI = confidence interval; ET = endocrine therapy; FUL = fulvestrant; HR = hazard ratio; IQR = interquartile range; IPTW = inverse probability of treatment weighting; mg = milligrams; NR = not reported; OS = overall survival; PFS = progression-free survival; PSM = propensity score matching; RWE = real-world evidence; sIPTW = stabilized inverse probability treatment weighting.

Table S5. Progression-free survival and overall survival for first-line palbociclib in comparative RWE studies versus ribociclib (subgroups; studies with manuscript data available)

Study Name; Reference	Treatment	Subgroup	Sample size	Follow-up Time	Starting dose for CDK4/6i, n (%)	PFS			OS		
						Median (95% CI), months	HR (95% CI); P value	At latest timepoint, n (%)	Median (95% CI), months	HR (95% CI); P value	At latest timepoint, n (%)
CDK-PREDICT study 633-Tolosa-2025; Spain	Palbociclib + ET	Luminal	72	Median 38.5 (IQR: 26.5-53.8) months	NR	27.3 (NR)	Palbociclib better: 1.38 (0.87-2.21); P=0.166	NR	85.8 (NR)	Palbociclib better: 1.43 (0.83-2.47); P=0.194	NR
	Ribociclib + ET	Luminal	70		NR	25.1 (NR)		NR	55.4 (NR)		NR
	Palbociclib + ET	Non-luminal	14		NR	9.2 (NR)	Palbociclib better: 0.77 (0.33-1.82); P=0.55	NR	30.2 (NR)	Palbociclib better: 1.24 (0.43-3.62); P=0.692	NR
	Ribociclib + ET	Non-luminal	12		NR	13.6 (NR)		NR	30.9 (NR)		NR
3810-Kahraman-2023; Japan	Palbociclib + Letrozole	De novo	157	NR	NR	32.2 (12.4-51.97)	NR; P=0.047	NR	NR	NR	NR
	Ribociclib + Letrozole	De novo	193	NR	NR	29.4 (13.45-45.42)		NR	NR	NR	NR
	Palbociclib + Letrozole	Prior chemotherapy/ endocrine resistant	NR	NR	NR	14.0 (7.7-20.4)	NR; P=0.021	NR	NR	NR	NR
	Ribociclib + Letrozole	Prior chemotherapy/ endocrine resistant	NR	NR	NR	21.9 (13.4-30.5)		NR	NR	NR	NR

Study Name; Reference	Treatment	Subgroup	Sample size	Follow-up Time	Starting dose for CDK4/6i , n (%)	PFS			OS		
						Median (95% CI), months	HR (95% CI); P value	At latest timepoint , n (%)	Median (95% CI), months	HR (95% CI); P value	At latest timepoin t, n (%)
4112-Cejuela-2023; Spain	Palbociclib + ET	Endocrine sensitive	59	NR	NR	NR	NR; P=0.629	NR	NR	NR	NR
	Ribociclib + ET	Endocrine sensitive	34	NR	NR	NR		NR	NR	NR	NR
	Palbociclib + ET	Endocrine resistant	37	NR	NR	17.02 (NR)	NR, P=0.972	NR	NR	NR	NR
	Ribociclib + ET	Endocrine resistant	20	NR	NR	10.38 (NR)		NR	NR	NR	NR
	Palbociclib + ET	Visceral disease	50	NR	NR	Not reached	NR; P=0.255	NR	NR	NR	NR
	Ribociclib + ET	Visceral disease	32	NR	NR	23.16 (NR)		NR	NR	NR	NR
	Palbociclib + ET	Non-visceral disease	46	NR	NR	24.1 (NR)	NR; P=0.163	NR	NR	NR	NR
	Ribociclib + ET	Non-visceral disease	22	NR	NR	36.01 (NR)		NR	NR	NR	NR
4506-Tang-2023; United Kingdom	Palbociclib + ET	De novo	74	NR	NR	43.6 (NR)	NR	5 years: NR (31.43)	77.4 (NR)	NR	5 years: NR (62.38)
	Ribociclib + ET	De novo	12	NR	NR	Not reached	NR	5 years: NR (74.07)	Not reached	NR	5 years: NR (82.5)
	Palbociclib + ET	Recurrent	88	NR	NR	20.9 (NR)	NR	5 years: NR (14.69)	36.1 (NR)	NR	5 years: NR (37.98)

Study Name; Reference	Treatment	Subgroup	Sample size	Follow-up Time	Starting dose for CDK4/6i, n (%)	PFS			OS		
						Median (95% CI), months	HR (95% CI); P value	At latest timepoint, n (%)	Median (95% CI), months	HR (95% CI); P value	At latest timepoint, n (%)
	Ribociclib + ET	Recurrent	34	NR	NR	18.85 (NR)	NR	5 years: NR (19.1)	44.6 (NR)	NR	5 years: NR (27.98)
	Palbociclib + ET	ER+/PR+	114	NR	NR	30.2 (NR)	NR	5 years: NR (22.66)	62.6 (NR)	NR	5 years: NR (50.41)
	Ribociclib + ET	ER+/PR+	31	NR	NR	44 (NR)	NR	5 years: NR (41.82)	54.8 (NR)	NR	5 years: NR (45.37)
	Palbociclib + ET	ER+/PR-	48	NR	NR	21.3 (NR)	NR	5 years: NR (21.07)	49.5 (NR)	NR	5 years: NR (39.48)
	Ribociclib + ET	ER+/PR-	15	NR	NR	10.1 (NR)	NR	5 years: NR (13.33)	34.3 (NR)	NR	5 years: NR (40)
	Palbociclib + ET	≤65 years	62	NR	NR	30.2 (NR)	NR	5 years: NR (25.56)	77.4 (NR)	NR	5 years: NR (55.27)
	Ribociclib + ET	≤65 years	25	NR	NR	20.5 (NR)	NR	5 years: NR (27.93)	44.6 (NR)	NR	5 years: NR (36.86)
	Palbociclib + ET	66-79 years	75	NR	NR	28.2 (NR)	NR	5 years: NR (25.61)	61.7 (NR)	NR	5 years: NR (50.17)
	Ribociclib + ET	66-79 years	16	NR	NR	24.7 (NR)	NR	5 years: NR (34.29)	54.8 (NR)	NR	5 years: NR (49.36)
	Palbociclib + ET	≥80 years	25	NR	NR	14.5 (NR)	NR	5 years: NR (0)	29.6 (NR)	NR	5 years: NR (23.34)
	Ribociclib + ET	≥80 years	5	NR	NR	68.2 (NR)	NR	5 years: NR (60)	Not reached	NR	5 years: NR (60)

Study Name; Reference	Treatment	Subgroup	Sample size	Follow-up Time	Starting dose for CDK4/6i, n (%)	PFS			OS		
						Median (95% CI), months	HR (95% CI); P value	At latest timepoint, n (%)	Median (95% CI), months	HR (95% CI); P value	At latest timepoint, n (%)
5893-Skocilic-2024; Croatia	Ribociclib + ET	Progesterone <10%	NR	NR	NR	NR	NR	NR	3.7 (1.5-3.8)	NR	NR
	Palbociclib + ET		NR	NR	NR	NR	NR	NR	1.5 (0.5-1.5)	NR	NR
	Ribociclib + ET	Liver metastasis	NR	NR	NR	NR	NR	NR	1.6 (0.7-1.6)	NR	NR
	Palbociclib + ET		NR	NR	NR	NR	NR	NR	1.4 (0.5-1.7)	NR	NR
CEPRA; 6138-Dajsakdipon-2024; Thailand	Ribociclib + AI	Age (per 1 year)	NR	NR	NR	NR	NR	NR	NR	Ribociclib similar to palbociclib: 1.00 (0.99–1.02); P=0.61	NR
	Palbociclib + AI	Age (per 1 year)	NR	NR	NR	NR	NR	NR	NR		NR
	Ribociclib + AI	ECOG PS (per 1 ECOG)	NR	NR	NR	NR	NR	NR	NR	Ribociclib better: 3.01 (1.08-8.37); P=0.03	NR
	Palbociclib + AI	ECOG PS (per 1 ECOG)	NR	NR	NR	NR	NR	NR	NR		NR
6522-Karhan-2024; Turkey	Palbociclib + ET	Without PPI	60	Median 15.6 (1.8–45) months	125mg; NR	22.2 (18.6–25.9)	NR	NR	NR	NR	NR
	Ribociclib + ET	Without PPI	61		600mg; NR	20.7 (14.5–26.8)	NR	NR	NR	NR	NR

Study Name; Reference	Treatment	Subgroup	Sample size	Follow-up Time	Starting dose for CDK4/6i , n (%)	PFS			OS		
						Median (95% CI), months	HR (95% CI); P value	At latest timepoint , n (%)	Median (95% CI), months	HR (95% CI); P value	At latest timepoin t, n (%)
PALMARES-2 540-Provenzano-2025; Italy	Palbociclib + ET	Endocrine sensitive	479	45.7 (IQR: 28.0-59.6) months	NR	NR	Ribociclib better: 0.88 (0.63-1.22); P=0.443	NR	NR	NR	NR
	Ribociclib + ET	Endocrine sensitive	567	Median 25.2 (IQR: 12.7-44.4) months	NR	NR		NR	NR	NR	NR
	Palbociclib + ET	Endocrine resistant	310	45.7 (IQR: 28.0-59.6) months	NR	NR	Ribociclib better: 0.75 (0.58-0.98); P=0.034	NR	NR	NR	NR
	Ribociclib + ET	Endocrine resistant	169	Median 25.2 (IQR: 12.7-44.4) months	NR	NR		NR	NR	NR	NR
	Palbociclib + ET	Luminal B-like	NR	45.7 (IQR: 28.0-59.6) months	NR	NR	Ribociclib better: 0.81 (0.75-0.88); P<0.001	NR	NR	NR	NR
	Ribociclib + ET	Luminal B-like	NR	Median 25.2 (IQR: 12.7-44.4) months	NR	NR		NR	NR	NR	NR

Study Name; Reference	Treatment	Subgroup	Sample size	Follow-up Time	Starting dose for CDK4/6i, n (%)	PFS			OS		
						Median (95% CI), months	HR (95% CI); P value	At latest timepoint, n (%)	Median (95% CI), months	HR (95% CI); P value	At latest timepoint, n (%)
	Palbociclib + ET	Liver metastasis	170	45.7 (IQR: 28.0-59.6) months	NR	NR	Ribociclib better: 0.89 (0.79-0.99); P=0.036	NR	NR	NR	NR
	Ribociclib + ET	Liver metastasis	120	Median 25.2 (IQR: 12.7-44.4) months	NR	NR		NR	NR	NR	NR
	Palbociclib + ET	Premenopausal	100	45.7 (IQR: 28.0-59.6) months	NR	NR	Ribociclib better: 0.57 (0.46-0.70); P<0.001	NR	NR	NR	NR
	Ribociclib + ET	Premenopausal	198	Median 25.2 (IQR: 12.7-44.4) months	NR	NR		NR	NR	NR	NR
	Palbociclib + ET	Poor ECOG PS	NR	45.7 (IQR: 28.0-59.6) months	NR	NR	Ribociclib better: 0.82 (0.66-1.01); P=0.062	NR	NR	NR	NR
	Ribociclib + ET	Poor ECOG PS	NR	Median 25.2 (IQR: 12.7-44.4) months	NR	NR		NR	NR	NR	NR

Study Name; Reference	Treatment	Subgroup	Sample size	Follow-up Time	Starting dose for CDK4/6i, n (%)	PFS			OS		
						Median (95% CI), months	HR (95% CI); P value	At latest timepoint, n (%)	Median (95% CI), months	HR (95% CI); P value	At latest timepoint, n (%)
	Palbociclib + ET	Older	NR	45.7 (IQR: 28.0-59.6) months	NR	NR	Palbociclib better: 1.09 (1.02-1.17); P=0.008	NR	NR	NR	NR
	Ribociclib + ET	Older	NR	Median 25.2 (IQR: 12.7-44.4) months	NR	NR		NR	NR	NR	NR
	Palbociclib + ET	Bone only	NR	45.7 (IQR: 28.0-59.6) months	NR	NR	Ribociclib better: 0.81 (0.60-1.09); P=0.170	NR	NR	NR	NR
	Ribociclib + ET	Bone only	NR	Median 25.2 (IQR: 12.7-44.4) months	NR	NR		NR	NR	NR	NR
	Palbociclib + ET	De novo metastatic	208	45.7 (IQR: 28.0-59.6) months	NR	NR	Ribociclib better: 0.76 (0.61-0.94); P=0.010	NR	NR	NR	NR
	Ribociclib + ET	De novo metastatic	264	Median 25.2 (IQR: 12.7-44.4) months	NR	NR		NR	NR	NR	NR

Study Name; Reference	Treatment	Subgroup	Sample size	Follow-up Time	Starting dose for CDK4/6i, n (%)	PFS			OS		
						Median (95% CI), months	HR (95% CI); P value	At latest timepoint, n (%)	Median (95% CI), months	HR (95% CI); P value	At latest timepoint, n (%)
PALMARES-2 655-Vernieri-2025; Italy	Ribociclib + ET	ILC, IPTW	NR	Median 29.8 months	NR	NR	Ribociclib better: 0.98 (0.68-1.45); P=0.949	NR	NR	NR	NR
	Palbociclib + ET	ILC, IPTW	NR		NR	NR		NR	NR	NR	NR
	Ribociclib + ET	ILC, Cox regression	NR		NR	NR	Ribociclib better: 0.96 (0.66-1.41); P=0.852	NR	NR	NR	NR
	Palbociclib + ET	ILC, Cox regression	NR		NR	NR		NR	NR	NR	NR
	Ribociclib + ET	Non-ILC, IPTW	NR	Median 31.5 months	NR	NR	Ribociclib better: 0.78 (0.65-0.93); P=0.005	NR	NR	NR	NR
	Palbociclib + ET	Non-ILC, IPTW	NR		NR	NR		NR	NR	NR	NR
	Ribociclib + ET	Non-ILC, Cox regression	NR		NR	NR	Ribociclib better: 0.79 (0.66-0.95); P=0.013	NR	NR	NR	NR
	Palbociclib + ET	Non-ILC, Cox regression	NR		NR	NR		NR	NR	NR	NR
OPAL registry; 6334-Thill-2024; Germany	Palbociclib + ET	M1 at diagnosis (after IPTW)	145	NR	NR	29.3 (24.1-33.2)	Ribociclib better: 0.89 (0.59-1.34); NR	NR	47.0 (34.1-NA)	Ribociclib better: 0.74 (0.42-1.30); NR	NR
	Ribociclib + ET	M1 at diagnosis (after IPTW)	81	NR	NR	31.6 (28.2-43.7)		NR	NA (42.1-NA)		NR

Study Name; Reference	Treatment	Subgroup	Sample size	Follow-up Time	Starting dose for CDK4/6i , n (%)	PFS			OS		
						Median (95% CI), months	HR (95% CI); P value	At latest timepoint , n (%)	Median (95% CI), months	HR (95% CI); P value	At latest timepoin t, n (%)
P-VERIFY 563-Rugo-2025; United States	Palbociclib + AI	sIPTW - Age 18-49 years	775	NR	NR	NR	NR	NR	NR	Ribociclib better: 0.79 (0.58-1.06); NR	NR
	Ribociclib + AI	sIPTW - Age 18-49 years	148	NR	NR	NR	NR	NR	NR		NR
	Palbociclib + AI	sIPTW - Age 50-64 years	2333	NR	NR	NR	NR	NR	NR	Ribociclib better: 0.96 (0.78-1.19); NR	NR
	Ribociclib + AI	sIPTW - Age 50-64 years	437	NR	NR	NR	NR	NR	NR		NR
	Palbociclib + AI	sIPTW - Age 65-74 years	2108	NR	NR	NR	NR	NR	NR	Ribociclib better: 0.99 (0.80-1.22); NR	NR
	Ribociclib + AI	sIPTW - Age 65-74 years	390	NR	NR	NR	NR	NR	NR		NR
	Palbociclib + AI	sIPTW - Age ≥ 75 years	1616	NR	NR	NR	NR	NR	NR	Palbociclib better: 1.07 (0.85-1.34); NR	NR
	Ribociclib + AI	sIPTW - Age ≥ 75 years	299	NR	NR	NR	NR	NR	NR		NR
	Palbociclib + AI	sIPTW - Race, White	4272	NR	NR	NR	NR	NR	NR	Palbociclib better: 1.06 (0.92-1.23); NR	NR
	Ribociclib + AI	sIPTW - Race, White	797	NR	NR	NR	NR	NR	NR		NR

Study Name; Reference	Treatment	Subgroup	Sample size	Follow-up Time	Starting dose for CDK4/6i, n (%)	PFS			OS		
						Median (95% CI), months	HR (95% CI); P value	At latest timepoint, n (%)	Median (95% CI), months	HR (95% CI); P value	At latest timepoint, n (%)
	Palbociclib + AI	sIPTW - Race, Black	638	NR	NR	NR	NR	NR	NR	Ribociclib better: 0.91 (0.60-1.36); NR	NR
	Ribociclib + AI	sIPTW - Race, Black	117	NR	NR	NR	NR	NR	NR		NR
	Palbociclib + AI	sIPTW - Race, other	1922	NR	NR	NR	NR	NR	NR	Ribociclib better: 0.84 (0.68-1.05); NR	NR
	Ribociclib + AI	sIPTW - Race, other	360	NR	NR	NR	NR	NR	NR		NR
	Palbociclib + AI	sIPTW - ECOG PS at baseline, 0	2444	NR	NR	NR	NR	NR	NR	Ribociclib better: 0.97 (0.80-1.17); NR	NR
	Ribociclib + AI	sIPTW - ECOG PS at baseline, 0	457	NR	NR	NR	NR	NR	NR		NR
	Palbociclib + AI	sIPTW - ECOG PS at baseline, 1	1806	NR	NR	NR	NR	NR	NR	Palbociclib better: 1.08 (0.87-1.34); NR	NR
	Ribociclib + AI	sIPTW - ECOG PS at baseline, 1	329	NR	NR	NR	NR	NR	NR		NR
	Palbociclib + AI	sIPTW - ECOG PS at baseline, 2-4	780	NR	NR	NR	NR	NR	NR	Palbociclib better:	NR

Study Name; Reference	Treatment	Subgroup	Sample size	Follow-up Time	Starting dose for CDK4/6i, n (%)	PFS			OS		
						Median (95% CI), months	HR (95% CI); P value	At latest timepoint, n (%)	Median (95% CI), months	HR (95% CI); P value	At latest timepoint, n (%)
	Ribociclib + AI	sIPTW - ECOG PS at baseline, 2-4	147	NR	NR	NR	NR	NR	NR	1.05 (0.76-1.45); NR	NR
	Palbociclib + AI	sIPTW - ECOG PS at baseline, ND	1801	NR	NR	NR	NR	NR	NR	Ribociclib better:	NR
	Ribociclib + AI	sIPTW - ECOG PS at baseline, ND	341	NR	NR	NR	NR	NR	NR	0.82 (0.63-1.07); NR	NR
	Palbociclib + AI	sIPTW - De novo metastatic	3473	NR	NR	NR	NR	NR	NR	Ribociclib better:	NR
	Ribociclib + AI	sIPTW - De novo metastatic	645	NR	NR	NR	NR	NR	NR	0.91 (0.77-1.07); NR	NR
	Palbociclib + AI	sIPTW - No visceral disease	4458	NR	NR	NR	NR	NR	NR	Ribociclib similar:	NR
	Ribociclib + AI	sIPTW - No visceral disease	831	NR	NR	NR	NR	NR	NR	0.99 (0.85-1.14); NR	NR
	Palbociclib + AI	sIPTW - Visceral disease	2374	NR	NR	NR	NR	NR	NR	Ribociclib better:	NR
	Ribociclib + AI	sIPTW - Visceral disease	443	NR	NR	NR	NR	NR	NR	0.98 (0.80-1.19); NR	NR
	Palbociclib + AI	sIPTW - No bone-only disease	3661	NR	NR	NR	NR	NR	NR	Ribociclib better:	NR

Study Name; Reference	Treatment	Subgroup	Sample size	Follow-up Time	Starting dose for CDK4/6i , n (%)	PFS			OS		
						Median (95% CI), months	HR (95% CI); P value	At latest timepoint , n (%)	Median (95% CI), months	HR (95% CI); P value	At latest timepoint, n (%)
	Ribociclib + AI	sIPTW - No bone-only disease	683	NR	NR	NR	NR	NR	NR	0.98 (0.84-1.15); NR	NR
	Palbociclib + AI	sIPTW - Bone-only disease	3171	NR	NR	NR	NR	NR	NR	Ribociclib similar:	NR
	Ribociclib + AI	sIPTW - Bone-only disease	591	NR	NR	NR	NR	NR	NR	0.99 (0.83-1.17); NR	NR
	Palbociclib + AI	sIPTW - Metastatic sites, 1	4010	NR	NR	NR	NR	NR	NR	Ribociclib similar:	NR
	Ribociclib + AI	sIPTW - Metastatic sites, 1	745	NR	NR	NR	NR	NR	NR	1.00 (0.85-1.16); NR	NR
	Palbociclib + AI	sIPTW - Metastatic sites, 2	1585	NR	NR	NR	NR	NR	NR	Ribociclib similar:	NR
	Ribociclib + AI	sIPTW - Metastatic sites, 2	304	NR	NR	NR	NR	NR	NR	1.01 (0.81-1.27); NR	NR
	Palbociclib + AI	sIPTW - Metastatic sites, ≥ 3	615	NR	NR	NR	NR	NR	NR	Palbociclib better:	NR
	Ribociclib + AI	sIPTW - Metastatic sites, ≥ 3	111	NR	NR	NR	NR	NR	NR	1.06 (0.72-1.54); NR	NR
	Palbociclib + AI	sIPTW - Metastatic sites, ND	622	NR	NR	NR	NR	NR	NR	Ribociclib better:	NR

Study Name; Reference	Treatment	Subgroup	Sample size	Follow-up Time	Starting dose for CDK4/6i, n (%)	PFS			OS		
						Median (95% CI), months	HR (95% CI); P value	At latest timepoint, n (%)	Median (95% CI), months	HR (95% CI); P value	At latest timepoint, n (%)
	Ribociclib + AI	sIPTW - Metastatic sites, ND	114	NR	NR	NR	NR	NR	NR	0.78 (0.48-1.26); NR	NR
P-VERIFY SABS24-102-Rugo-2024[a]; United States	Palbociclib + AI	sIPTW - Age 18-49 years	775	NR	NR	NR	NR	NR	NR	Ribociclib better: 0.79 (0.58-1.06); NR	NR
	Ribociclib + AI	sIPTW - Age 18-49 years	148	NR	NR	NR	NR	NR	NR		NR
	Palbociclib + AI	sIPTW - Age 50-64 years	2333	NR	NR	NR	NR	NR	NR	Ribociclib better: 0.96 (0.78-1.19); NR	NR
	Ribociclib + AI	sIPTW - Age 50-64 years	437	NR	NR	NR	NR	NR	NR		NR
	Palbociclib + AI	sIPTW - Age 65-75 years	2108	NR	NR	NR	NR	NR	NR	Ribociclib similar: 0.99 (0.80-1.22); NR	NR
	Ribociclib + AI	sIPTW - Age 65-75 years	390	NR	NR	NR	NR	NR	NR		NR
	Palbociclib + AI	sIPTW - Age ≥ 75 years	1616	NR	NR	NR	NR	NR	NR	Palbociclib better: 1.07 (0.85-1.34); NR	NR
	Ribociclib + AI	sIPTW - Age ≥ 75 years	299	NR	NR	NR	NR	NR	NR		NR
	Palbociclib + AI	sIPTW - Race, White	4272	NR	NR	NR	NR	NR	NR	Palbociclib better:	NR

Study Name; Reference	Treatment	Subgroup	Sample size	Follow-up Time	Starting dose for CDK4/6i , n (%)	PFS			OS		
						Median (95% CI), months	HR (95% CI); P value	At latest timepoint , n (%)	Median (95% CI), months	HR (95% CI); P value	At latest timepoin t, n (%)
	Ribociclib + AI	sIPTW - Race, White	797	NR	NR	NR	NR	NR	NR	1.06 (0.92-1.23); NR	NR
	Palbociclib + AI	sIPTW - Race, Black	638	NR	NR	NR	NR	NR	NR	Ribociclib better: 0.91 (0.60-1.36); NR	NR
	Ribociclib + AI	sIPTW - Race, Black	117	NR	NR	NR	NR	NR	NR		NR
	Palbociclib + AI	sIPTW - Race, other	1922	NR	NR	NR	NR	NR	NR	Ribociclib better: 0.84 (0.68-1.05); NR	NR
	Ribociclib + AI	sIPTW - Race, other	360	NR	NR	NR	NR	NR	NR		NR
	Palbociclib + AI	sIPTW - ECOG PS at baseline, 0	2444	NR	NR	NR	NR	NR	NR	Ribociclib better: 0.97 (0.80-1.17); NR	NR
	Ribociclib + AI	sIPTW - ECOG PS at baseline, 0	457	NR	NR	NR	NR	NR	NR		NR
	Palbociclib + AI	sIPTW - ECOG PS at baseline, 1	1806	NR	NR	NR	NR	NR	NR	Palbociclib better: 1.08 (0.87-1.34); NR	NR
	Ribociclib + AI	sIPTW - ECOG PS at baseline, 1	329	NR	NR	NR	NR	NR	NR		NR

Study Name; Reference	Treatment	Subgroup	Sample size	Follow-up Time	Starting dose for CDK4/6i, n (%)	PFS			OS		
						Median (95% CI), months	HR (95% CI); P value	At latest timepoint, n (%)	Median (95% CI), months	HR (95% CI); P value	At latest timepoint, n (%)
	Palbociclib + AI	sIPTW - ECOG PS at baseline, 2 -4	780	NR	NR	NR	NR	NR	NR	Palbociclib better: 1.05 (0.76-1.45); NR	NR
	Ribociclib + AI	sIPTW - ECOG PS at baseline, 2 -4	147	NR	NR	NR	NR	NR	NR		NR
	Palbociclib + AI	sIPTW - ECOG PS at baseline, ND	1801	NR	NR	NR	NR	NR	NR	Ribociclib better: 0.82 (0.63-1.07); NR	NR
	Ribociclib + AI	sIPTW - ECOG PS at baseline, ND	341	NR	NR	NR	NR	NR	NR		NR
	Palbociclib + AI	sIPTW - De novo metastatic	3473	NR	NR	NR	NR	NR	NR	Ribociclib better: 0.91 (0.77-1.07); NR	NR
	Ribociclib + AI	sIPTW - De novo metastatic	645	NR	NR	NR	NR	NR	NR		NR
	Palbociclib + AI	sIPTW - No visceral disease	4458	NR	NR	NR	NR	NR	NR	Ribociclib similar: 0.99 (0.85-1.14); NR	NR
	Ribociclib + AI	sIPTW - No visceral disease	831	NR	NR	NR	NR	NR	NR		NR
	Palbociclib + AI	sIPTW - Visceral disease	2374	NR	NR	NR	NR	NR	NR	Ribociclib better:	NR

Study Name; Reference	Treatment	Subgroup	Sample size	Follow-up Time	Starting dose for CDK4/6i , n (%)	PFS			OS		
						Median (95% CI), months	HR (95% CI); P value	At latest timepoint , n (%)	Median (95% CI), months	HR (95% CI); P value	At latest timepoin t, n (%)
	Ribociclib + AI	sIPTW - Visceral disease	443	NR	NR	NR	NR	NR	NR	0.98 (0.80-1.19); NR	NR
	Palbociclib + AI	sIPTW - No bone-only disease	3661	NR	NR	NR	NR	NR	NR	Ribociclib better: 0.98 (0.84-1.15); NR	NR
	Ribociclib + AI	sIPTW - No bone-only disease	683	NR	NR	NR	NR	NR	NR		NR
	Palbociclib + AI	sIPTW - Bone only disease	3171	NR	NR	NR	NR	NR	NR	Ribociclib similar: 0.99 (0.83-1.17); NR	NR
	Ribociclib + AI	sIPTW - Bone only disease	591	NR	NR	NR	NR	NR	NR		NR
	Palbociclib + AI	sIPTW - Metastatic sites, 1	4010	NR	NR	NR	NR	NR	NR	Ribociclib similar: 1.00 (0.85-1.16); NR	NR
	Ribociclib + AI	sIPTW - Metastatic sites, 1	745	NR	NR	NR	NR	NR	NR		NR
	Palbociclib + AI	sIPTW - metastatic sites, 2	1585	NR	NR	NR	NR	NR	NR	Ribociclib similar: 1.01 (0.81-1.27); NR	NR
	Ribociclib + AI	sIPTW - metastatic sites, 2	304	NR	NR	NR	NR	NR	NR		NR
	Palbociclib + AI	sIPTW - metastatic sites, ≥3	615	NR	NR	NR	NR	NR	NR	Palbociclib better:	NR

Study Name; Reference	Treatment	Subgroup	Sample size	Follow-up Time	Starting dose for CDK4/6i , n (%)	PFS			OS		
						Median (95% CI), months	HR (95% CI); P value	At latest timepoint , n (%)	Median (95% CI), months	HR (95% CI); P value	At latest timepoint, n (%)
	Ribociclib + AI	sIPTW - metastatic sites, ≥3	111	NR	NR	NR	NR	NR	NR	1.06 (0.72-1.54); NR	NR
	Palbociclib + AI	sIPTW - metastatic sites, NS	622	NR	NR	NR	NR	NR	NR	Ribociclib better: 0.78 (0.48-1.26); NR	NR
	Ribociclib + AI	sIPTW - metastatic sites, NS	114	NR	NR	NR	NR	NR	NR		NR
P-VERIFY 1132-Rugo-2025; United States	Palbociclib + AI	sIPTW - Age 18-49 years	775	NR	NR	NR	Ribociclib similar: 1.01 (0.81-1.28); NR	NR	NR	NR	NR
	Ribociclib + AI	sIPTW - Age 18-49 years	148	NR	NR	NR		NR	NR	NR	NR
	Palbociclib + AI	sIPTW - Age 50-64 years	2333	NR	NR	NR	Ribocilib better: 0.96 (0.81-1.13); NR	NR	NR	NR	NR
	Ribociclib + AI	sIPTW - Age 50-64 years	437	NR	NR	NR		NR	NR	NR	NR
	Palbociclib + AI	sIPTW - Age 65-74 years	2108	NR	NR	NR	Ribocilib better: 0.97 (0.81-1.16); NR	NR	NR	NR	NR
	Ribociclib + AI	sIPTW - Age 65-74 years	390	NR	NR	NR		NR	NR	NR	NR
	Palbociclib + AI	sIPTW - Age ≥ 75 years	1616	NR	NR	NR	Ribocilib better:	NR	NR	NR	NR

Study Name; Reference	Treatment	Subgroup	Sample size	Follow-up Time	Starting dose for CDK4/6i, n (%)	PFS			OS		
						Median (95% CI), months	HR (95% CI); P value	At latest timepoint, n (%)	Median (95% CI), months	HR (95% CI); P value	At latest timepoint, n (%)
	Ribociclib + AI	sIPTW - Age ≥ 75 years	299	NR	NR	NR	0.98 (0.80-1.22); NR	NR	NR	NR	NR
	Palbociclib + AI	sIPTW - Race, White	4272	NR	NR	NR	Ribocilib better: 0.97 (0.86-1.10); NR	NR	NR	NR	NR
	Ribociclib + AI	sIPTW - Race, White	797	NR	NR	NR		NR	NR	NR	NR
	Palbociclib + AI	sIPTW - Race, Black	638	NR	NR	NR	Ribocilib better: 0.94 (0.68-1.28); NR	NR	NR	NR	NR
	Ribociclib + AI	sIPTW - Race, Black	117	NR	NR	NR		NR	NR	NR	NR
	Palbociclib + AI	sIPTW - Race, other	1922	NR	NR	NR	Ribocilib better: 0.98 (0.82-1.17); NR	NR	NR	NR	NR
	Ribociclib + AI	sIPTW - Race, other	360	NR	NR	NR		NR	NR	NR	NR
	Palbociclib + AI	sIPTW - ECOG PS at baseline, 0	2444	NR	NR	NR	Ribocilib better: 0.95 (0.81-1.12); NR	NR	NR	NR	NR
	Ribociclib + AI	sIPTW - ECOG PS at baseline, 0	457	NR	NR	NR		NR	NR	NR	NR
	Palbociclib + AI	sIPTW - ECOG PS at baseline, 1	1806	NR	NR	NR	Ribocilib better: 0.96 (0.80-1.16); NR	NR	NR	NR	NR

Study Name; Reference	Treatment	Subgroup	Sample size	Follow-up Time	Starting dose for CDK4/6i, n (%)	PFS			OS		
						Median (95% CI), months	HR (95% CI); P value	At latest timepoint, n (%)	Median (95% CI), months	HR (95% CI); P value	At latest timepoint, n (%)
	Ribociclib + AI	sIPTW - ECOG PS at baseline, 1	329	NR	NR	NR		NR	NR	NR	NR
	Palbociclib + AI	sIPTW - ECOG PS at baseline, 2-4	780	NR	NR	NR	Palbociclib better: 1.13 (0.87-1.45); NR	NR	NR	NR	NR
	Ribociclib + AI	sIPTW - ECOG PS at baseline, 2-4	147	NR	NR	NR		NR	NR	NR	NR
	Palbociclib + AI	sIPTW - ECOG PS at baseline, ND	1801	NR	NR	NR	Ribocilib better: 0.91 (0.74-1.13); NR	NR	NR	NR	NR
	Ribociclib + AI	sIPTW - ECOG PS at baseline, ND	341	NR	NR	NR		NR	NR	NR	NR
	Palbociclib + AI	sIPTW - De novo metastatic	3473	NR	NR	NR	Ribocilib better: 0.90 (0.79-1.04); NR	NR	NR	NR	NR
	Ribociclib + AI	sIPTW - De novo metastatic	645	NR	NR	NR		NR	NR	NR	NR
	Palbociclib + AI	sIPTW - No visceral disease	4458	NR	NR	NR	Ribocilib better: 0.92 (0.81-1.04); NR	NR	NR	NR	NR
	Ribociclib + AI	sIPTW - No visceral disease	831	NR	NR	NR		NR	NR	NR	NR

Study Name; Reference	Treatment	Subgroup	Sample size	Follow-up Time	Starting dose for CDK4/6i , n (%)	PFS			OS		
						Median (95% CI), months	HR (95% CI); P value	At latest timepoint , n (%)	Median (95% CI), months	HR (95% CI); P value	At latest timepoin t, n (%)
	Palbociclib + AI	sIPTW - Visceral disease	2374	NR	NR	NR	Palbociclib better: 1.07 (0.91-1.24); NR	NR	NR	NR	NR
	Ribociclib + AI	sIPTW - Visceral disease	443	NR	NR	NR		NR	NR	NR	NR
	Palbociclib + AI	sIPTW - No bone-only disease	3661	NR	NR	NR	Ribociclib similar: 0.99 (0.86-1.13); NR	NR	NR	NR	NR
	Ribociclib + AI	sIPTW - No bone-only disease	683	NR	NR	NR		NR	NR	NR	NR
	Palbociclib + AI	sIPTW - Bone-only disease	3171	NR	NR	NR	Ribocilib better: 0.95 (0.83-1.10); NR	NR	NR	NR	NR
	Ribociclib + AI	sIPTW - Bone-only disease	591	NR	NR	NR		NR	NR	NR	NR
	Palbociclib + AI	sIPTW - Metastatic sites, 1	4010	NR	NR	NR	Ribocilib better: 0.97 (0.86-1.11); NR	NR	NR	NR	NR
	Ribociclib + AI	sIPTW - Metastatic sites, 1	745	NR	NR	NR		NR	NR	NR	NR
	Palbociclib + AI	sIPTW - Metastatic sites, 2	1585	NR	NR	NR	Palbociclib better: 1.04 (0.86-1.25); NR	NR	NR	NR	NR
	Ribociclib + AI	sIPTW - Metastatic sites, 2	304	NR	NR	NR		NR	NR	NR	NR

Study Name; Reference	Treatment	Subgroup	Sample size	Follow-up Time	Starting dose for CDK4/6i, n (%)	PFS			OS		
						Median (95% CI), months	HR (95% CI); P value	At latest timepoint, n (%)	Median (95% CI), months	HR (95% CI); P value	At latest timepoint, n (%)
	Palbociclib + AI	sIPTW - Metastatic sites, ≥ 3	615	NR	NR	NR	Palbociclib better; 1.05 (0.78-1.42); NR	NR	NR	NR	NR
	Ribociclib + AI	sIPTW - Metastatic sites, ≥ 3	111	NR	NR	NR		NR	NR	NR	NR
	Palbociclib + AI	sIPTW - Metastatic sites, ND	622	NR	NR	NR	Ribocilib better: 0.66 (0.42-1.02); NR	NR	NR	NR	NR
	Ribociclib + AI	sIPTW - Metastatic sites, ND	114	NR	NR	NR		NR	NR	NR	NR

Note: For records with a high volume of subgroup data, subgroups outlined in Appendix F were prioritized.
Note: The "Study Name; Reference" cells shaded in grey highlight full text records, while non-shaded are abstracts/manuscripts of the parent study.
Note: Overall follow-up time was used where subgroup-specific follow-up times were not reported.
[a] Different data reported in the poster and the abstract.
Abbreviations: AI = aromatase inhibitor; CDK4/6i = cyclin-dependent kinase 4/6 inhibitors; CI = confidence interval; ECOG PS = eastern cooperative oncology group performance status; ER = estrogen receptor; ET = endocrine therapy; HR = hazard ratio; ILC = invasive lobular carcinoma; IQR = interquartile range; IPTW = inverse probability of treatment weighting; M1 = metastasic; mg = milligrams; ND = not documented; NR = not reported; OS = overall survival; PFS = progression-free survival; PPI = proton pump inhibitor; PR = progesterone receptor; RWE = real-world evidence; sIPTW = stabilized inverse probability treatment weighting.

Table S6. Progression-free survival and overall survival for first-line palbociclib in comparative RWE studies versus abemaciclib (overall population; studies with manuscript data available)

Study Name; Reference	Treatment	Subgroup	Sample size	Follow-up Time	Starting dose for CDK4/6i, n (%)	PFS			OS		
						Median (95% CI), months	HR (95% CI); P value	At latest timepoint, n (%)	Median (95% CI), months	HR (95% CI); P value	At latest timepoint, n (%)
4112-Cejuela-2023; Spain	Abemaciclib + ET	All patients	56	Median 40.28 months	NR	39.49 (NR)	NR	NR	Not reached	NR	NR
	Palbociclib + ET	All patients	96	Median 75 months	NR	30.03 (NR)	NR	NR	Not reached	NR	NR
4132-Buller-2023; United States	Palbociclib + ET	All patients	115	NR	NR	23.00 (17.63-28.37)	NR	NR	44.30 (31.16-57.44)	NR	NR
	Abemaciclib + ET	All patients	44	NR	NR	17.00 (10.41-23.59)	NR	NR	34.30 (NR)	NR	NR
4506-Tang-2023; United Kingdom	Palbociclib + ET	All patients	162	Median 49.5 months	NR	27.5 (NR)	NR	5 years: NR (20.88)	49.5 (NR)	NR	5 years: NR (48.54)
	Abemaciclib + ET	All patients	19		NR	Not reached	NR	5 years: NR (66.8)	Not reached	NR	5 years: NR (66.9)
6590-Gehrchen-2024; Denmark	Abemaciclib + ET	All patients	322	Median 24.8 (95%CI: 24.1-25.8) months	NR	Not reached	Abemaciclib better: 0.74 (0.60-0.90); P=0.005	24 months: NR (65)	37.8 (32.5-NA)	NR	NR
	Palbociclib + ET	All patients	873	Median 55.1 (95%CI: 52.1-58.0) months	NR	32.0 (28.9-35.3)		24 months: NR (57)	49.7 (44.7-54.1)	NR	NR

Study Name; Reference	Treatment	Subgroup	Sample size	Follow-up Time	Starting dose for CDK4/6i, n (%)	PFS			OS		
						Median (95% CI), months	HR (95% CI); P value	At latest timepoint, n (%)	Median (95% CI), months	HR (95% CI); P value	At latest timepoint, n (%)
YOUNGBC-28; 6637-Chen-2024[a]; China	Palbociclib + ET	After PSM	56	Median 21.8 months	NR	22.1 (18.1-27.3)	Palbociclib better: 0.981 (0.578-1.665); P=0.94	NR	NR	NR	NR
	Abemaciclib + ET	After PSM	56		NR	20.5 (16.0-NA)		NR	NR	NR	NR
CDK-PREDICT study 633-Tolosa-2025; Spain	Palbociclib + ET	All patients	86	Median 38.5 (IQR: 26.5-53.8) months	NR	20.7 (16.6-33.3)	NR	NR	85.8 (47.6-not reached)	NR	NR
	Abemaciclib + ET	All patients	17		NR	12.4 (8.0-not reached)	NR	NR	46.9 (22.5-not reached)	NR	NR
694-Yoshinami-2025; Japan	Palbociclib + ET	All patients	281	Median 32.5 (range 2.5-101.0) months	NR	32.2 (24.1-40.9)	NR	3 year: NR (47.6)	NR	NR	3 year: NR (85.8)
	Abemaciclib + ET	All patients	252		NR	48.7 (31.4 - not reached)	NR	3 year: NR (54.2)	NR	NR	3 year: NR (80.6)
PALMARES-2 540-Provenzano-2025[a]; Italy	Abemaciclib + ET	IPTW-adjusted analyses	NR	Median 22.4 (IQR: 12.3-33.8) months	NR	46.6 (33.6-NA)	Abemaciclib better: 0.78 (0.64-0.95); P=0.015	NR	NR	NR	NR
	Palbociclib + ET	IPTW-adjusted analyses	NR	45.7 (IQR: 28.0-59.6) months	NR	29.4 (25.9-33.3)		NR	NR	NR	NR
PALMARES-2 883-Vernieri-2025; Italy	Palbociclib + ET	IPTW	1392	Median 52.4 months	NR	NR	Abemaciclib better:	NR	NR	Abemaciclib better:	NR

Study Name; Reference	Treatment	Subgroup	Sample size	Follow-up Time	Starting dose for CDK4/6i, n (%)	PFS			OS		
						Median (95% CI), months	HR (95% CI); P value	At latest timepoint, n (%)	Median (95% CI), months	HR (95% CI); P value	At latest timepoint, n (%)
	Abemaciclib + ET	IPTW	798	Median 29.6 months	NR	NR	0.88 (0.77-0.99); P=0.04	NR	NR	0.91 (0.76-1.09); P=0.3	NR
PALMARES-2; 5854-Vernieri-2024; Italy	Palbociclib + ET	All patients	750	NR	NR	NR	Abemaciclib better: 0.71 (0.56-0.90); P=0.005	NR	NR	NR	NR
	Abemaciclib + ET	All patients	424	NR	NR	NR		NR	NR	NR	NR
PALMARES-2; SABCS24-038-Vernieri-2024; Italy	Palbociclib + ET	All patients	789	NR	NR	NR	Abemaciclib better: 0.77 (0.62-0.94); P=0.45	NR	NR	NR	NR
	Abemaciclib + ET	All patients	457	NR	NR	NR		NR	NR	NR	NR
P-VERIFY 1132-Rugo-2025[a]	Abemaciclib + AI	After sIPTW adjustment	1038	Median 21.5 (IQR: 25.0) months	NR	22.9 (20.2-26.5)	Abemaciclib better: 0.96 (0.86-1.06) P=0.3889	NR	NR	NR	NR
	Palbociclib + AI	After sIPTW adjustment	6832	Median 33.0 (IQR: 34.7) months	NR	22.7 (21.6-23.8)		NR	NR	NR	NR
P-VERIFY 563-Rugo-2025[a]; United States	Abemaciclib + AI	After sIPTW adjustment	1038	Median 21.5 (IQR: 25.0) months	NR	NR	NR	NR	64.5 (55.4-NE)	Abemaciclib better: 0.95 (0.84-1.08); P=0.4292	30 months: NR (71.5)
	Palbociclib + AI	After sIPTW adjustment	6832	Median 33.0 (IQR:	NR	NR	NR	NR	54.6 (52.6-56.4)		30 months:

Study Name; Reference	Treatment	Subgroup	Sample size	Follow-up Time	Starting dose for CDK4/6i, n (%)	PFS			OS		
						Median (95% CI), months	HR (95% CI); P value	At latest timepoint, n (%)	Median (95% CI), months	HR (95% CI); P value	At latest timepoint, n (%)
				34.7) months							NR (71.4)
P-VERIFY SABS24-102-Rugo-2024[a,b]; United States	Palbociclib + AI	sIPTW	6832	Median 33.0 (IQR: 34.7) months	NR	NR	NR	NR	54.6 (52.6-56.4)	Abemaciclib better: 0.95 (0.84-1.08); P=0.4292	30 month: NR (71.4)
	Abemaciclib + AI	sIPTW	1038	Median 21.5 (IQR: 25.0) months	NR	NR	NR	NR	64.5 (55.4-NE)		30 month: NR (71.5)

Note: The "Study Name; Reference" cells shaded in grey highlight full text records, while non-shaded are abstracts/manuscripts of the parent study.

[a] Record also reports unadjusted data.

[b] Different data reported in the poster and the abstract.

Abbreviations: AI = aromatase inhibitor; CDK4/6i = cyclin-dependent kinase 4/6 inhibitors; CI = confidence interval; ET = endocrine therapy; HR = hazard ratio; IQR = interquartile range; IPTW = inverse probability of treatment weighting; NA = not available; NE = not evaluable; NR = not reported; OS = overall survival; PFS = progression-free survival; PSM = propensity score matching; RWE = real-world evidence; sIPTW = stabilized inverse probability treatment weighting.

Table S7. Progression-free survival and overall survival for first-line palbociclib in comparative RWE studies versus abemaciclib (subgroups; studies with manuscript data available)

Study Name; Reference	Treatment	Subgroup	Sample size	Follow-up Time	Starting dose for CDK4/6i, n (%)	PFS			OS		
						Median (95% CI), months	HR (95% CI); P value	At latest timepoint, n (%)	Median (95% CI), months	HR (95% CI); P value	At latest timepoint, n (%)
4112-Cejuela-2023; Spain	Abemaciclib + ET	Endocrine sensitive	35	NR	NR	Not reached	Abemaciclib better: 2.41 (1.09-5.31); P=0.029	NR	NR	NR	NR
	Palbociclib + ET	Endocrine sensitive	59	NR	NR	17.02 (NR)		NR	NR	NR	NR
	Abemaciclib + ET	Endocrine resistant	21	NR	NR	NR	NR; P=0.027	NR	NR	NR	NR
	Palbociclib + ET	Endocrine resistant	37	NR	NR	NR		NR	NR	NR	NR
	Abemaciclib + ET	Visceral disease	34	NR	NR	39.49 (NR)	NR; P=0.937	NR	NR	NR	NR
	Palbociclib + ET	Visceral disease	50	NR	NR	Not reached		NR	NR	NR	NR
	Abemaciclib + ET	Non-visceral disease	22	NR	NR	Not reached	NR; P=0.038	NR	NR	NR	NR
	Palbociclib + ET	Non-visceral disease	46	NR	NR	24.1 (NR)		NR	NR	NR	NR
4506-Tang-2023; United Kingdom	Palbociclib + ET	De novo	74	NR	NR	43.6 (NR)	NR	5 years: NR (31.43)	77.4 (NR)	NR	5 years: NR (62.38)
	Abemaciclib + ET	De novo	11	NR	NR	Not reached	NR	NR	Not reached	NR	NR

Study Name; Reference	Treatment	Subgroup	Sample size	Follow-up Time	Starting dose for CDK4/6i, n (%)	PFS			OS		
						Median (95% CI), months	HR (95% CI); P value	At latest timepoint, n (%)	Median (95% CI), months	HR (95% CI); P value	At latest timepoint, n (%)
	Palbociclib + ET	Recurrent	88	NR	NR	20.9 (NR)	NR	5 years: NR (14.69)	36.1 (NR)	NR	5 years: NR (37.98)
	Abemaciclib + ET	Recurrent	8	NR	NR	Not reached	NR	NR	30.7 (NR)	NR	NR
	Palbociclib + ET	ER+/PR+	114	NR	NR	30.2 (NR)	NR	5 years: NR (22.66)	62.6 (NR)	NR	5 years: NR (50.41)
	Abemaciclib + ET	ER+/PR+	15	NR	NR	Not reached	NR	NR	Not reached	NR	NR
	Palbociclib + ET	ER+/PR-	48	NR	NR	21.3 (NR)	NR	5 years: NR (21.07)	49.5 (NR)	NR	5 years: NR (39.48)
	Abemaciclib + ET	ER+/PR-	4	NR	NR	28.3 (NR)	NR	NR	30.2 (NR)	NR	NR
	Palbociclib + ET	≤65 years	62	NR	NR	30.2 (NR)	NR	5 years: NR (25.56)	77.4 (NR)	NR	5 years: NR (55.27)
	Abemaciclib + ET	≤65 years	8	NR	NR	Not reached	NR	NR	36.5 (NR)	NR	NR
	Palbociclib + ET	66-79 years	75	NR	NR	28.2 (NR)	NR	5 years: NR (25.61)	61.7 (NR)	NR	5 years: NR (50.17)
	Abemaciclib + ET	66-79 years	6	NR	NR	Not reached	NR	NR	Not reached	NR	NR
	Palbociclib + ET	≥80 years	25	NR	NR	14.5 (NR)	NR	5 years: (0)	29.6 (NR)	NR	5 years: NR (23.34)

Study Name; Reference	Treatment	Subgroup	Sample size	Follow-up Time	Starting dose for CDK4/6i, n (%)	PFS			OS		
						Median (95% CI), months	HR (95% CI); P value	At latest timepoint, n (%)	Median (95% CI), months	HR (95% CI); P value	At latest timepoint, n (%)
	Abemaciclib + ET	≥80 years	5	NR	NR	Not reached	NR	NR	Not reached	NR	NR
5893-Skocilic-2024; Croatia	Abemaciclib + ET	Liver metastasis	NR	NR	NR	NR	NR	NR	3.9 (2.8-not reached)	NR	NR
	Palbociclib + ET		NR	NR	NR	NR	NR	NR	1.4 (0.5-1.7)	NR	NR
	Abemaciclib + ET	Progesterone <10%	NR	NR	NR	NR	NR	NR	3.9 (1.4-3.9)	NR	NR
	Palbociclib + ET		NR	NR	NR	NR	NR	NR	1.5 (0.5-1.5)	NR	NR
YOUNGBC-28; 6637-Chen-2024; China	Palbociclib + ET	Primary resistance	13	NR	NR	7.6 (6.4-NA)	NR	NR	NR	NR	NR
	Abemaciclib + ET	Primary resistance	14	NR	NR	9 (6.0-NA)	NR	NR	NR	NR	NR
	Palbociclib + ET	Endocrine naïve	30	NR	NR	20.9 (12.7-NA)	NR	NR	NR	NR	NR
	Abemaciclib + ET	Endocrine naïve	19	NR	NR	22.0 (15.0-NA)	NR	NR	NR	NR	NR
	Palbociclib + ET	Secondary resistance	45	NR	NR	22.0 (13.0-30)	NR	NR	NR	NR	NR
	Abemaciclib + ET	Secondary resistance	46	NR	NR	23.5 (16.0-NA)	NR	NR	NR	NR	NR

Study Name; Reference	Treatment	Subgroup	Sample size	Follow-up Time	Starting dose for CDK4/6i , n (%)	PFS			OS		
						Median (95% CI), months	HR (95% CI); P value	At latest timepoint , n (%)	Median (95% CI), months	HR (95% CI); P value	At latest timepoin t, n (%)
	Palbociclib + ET	Visceral crisis	9	NR	NR	22.5 (19.0-NA)	NR	NR	NR	NR	NR
	Abemaciclib + ET	Visceral crisis	15	NR	NR	6.1 (5.0-NA)	NR	NR	NR	NR	NR
	Palbociclib + ET	No visceral crisis	79	NR	NR	14.6 (12.4-22.6)	NR	NR	NR	NR	NR
	Abemaciclib + ET	No visceral crisis	64	NR	NR	20.5 (16.0-NA)	NR	NR	NR	NR	NR
PALMARES-2 540-Provenzano-2025; Italy	Abemaciclib + ET	Endocrine sensitive	287	Median 22.4 (IQR: 12.3-33.8) months	NR	NR	Abemaciclib better: 0.75 (0.64-0.87); P<0.001	NR	NR	NR	NR
	Palbociclib + ET	Endocrine sensitive	479	45.7 (IQR: 28.0-59.6) months	NR	NR		NR	NR	NR	NR
	Abemaciclib + ET	Endocrine resistant	170	Median 22.4 (IQR: 12.3-33.8) months	NR	NR	Abemaciclib better: 0.77 (0.63-0.93); P=0.008	NR	NR	NR	NR
	Palbociclib + ET	Endocrine resistant	310	45.7 (IQR: 28.0-59.6) months	NR	NR		NR	NR	NR	NR

Study Name; Reference	Treatment	Subgroup	Sample size	Follow-up Time	Starting dose for CDK4/6i, n (%)	PFS			OS		
						Median (95% CI), months	HR (95% CI); P value	At latest timepoint, n (%)	Median (95% CI), months	HR (95% CI); P value	At latest timepoint, n (%)
	Abemaciclib + ET	Luminal B-like	NR	Median 22.4 (IQR: 12.3-33.8) months	NR	NR	Abemaciclib better: 0.76 (0.65-0.90); P=0.002	NR	NR	NR	NR
	Palbociclib + ET	Luminal B-like	NR	45.7 (IQR: 28.0-59.6) months	NR	NR		NR	NR	NR	NR
	Abemaciclib + ET	Liver metastasis	NR	Median 22.4 (IQR: 12.3-33.8) months	NR	NR	Abemaciclib better: 0.73 (0.45-1.17); P=0.193	NR	NR	NR	NR
	Palbociclib + ET	Liver metastasis	NR	45.7 (IQR: 28.0-59.6) months	NR	NR		NR	NR	NR	NR
	Abemaciclib + ET	Premenopausal	NR	Median 22.4 (IQR: 12.3-33.8) months	NR	NR	Abemaciclib better: 0.59 (0.39-0.89); P=0.013	NR	NR	NR	NR
	Palbociclib + ET	Premenopausal	NR	45.7 (IQR: 28.0-59.6) months	NR	NR		NR	NR	NR	NR

Study Name; Reference	Treatment	Subgroup	Sample size	Follow-up Time	Starting dose for CDK4/6i, n (%)	PFS			OS		
						Median (95% CI), months	HR (95% CI); P value	At latest timepoint, n (%)	Median (95% CI), months	HR (95% CI); P value	At latest timepoint, n (%)
	Abemaciclib + ET	Poor ECOG PS	NR	Median 22.4 (IQR: 12.3-33.8) months	NR	NR	Abemaciclib better: 0.74 (0.55-0.99); P=0.048	NR	NR	NR	NR
	Palbociclib + ET	Poor ECOG PS	NR	45.7 (IQR: 28.0-59.6) months	NR	NR		NR	NR	NR	NR
	Abemaciclib + ET	Older	NR	Median 22.4 (IQR: 12.3-33.8) months	NR	NR	Abemaciclib better: 0.89 (0.72-1.11); P=0.304	NR	NR	NR	NR
	Palbociclib + ET	Older	NR	45.7 (IQR: 28.0-59.6) months	NR	NR		NR	NR	NR	NR
	Abemaciclib + ET	Bone only	NR	Median 22.4 (IQR: 12.3-33.8) months	NR	NR	Abemaciclb better: 0.88 (0.60-1.27); P=0.489	NR	NR	NR	NR
	Palbociclib + ET	Bone only	NR	45.7 (IQR: 28.0-59.6) months	NR	NR		NR	NR	NR	NR

Study Name; Reference	Treatment	Subgroup	Sample size	Follow-up Time	Starting dose for CDK4/6i, n (%)	PFS			OS		
						Median (95% CI), months	HR (95% CI); P value	At latest timepoint, n (%)	Median (95% CI), months	HR (95% CI); P value	At latest timepoint, n (%)
	Abemaciclib + ET	De novo metastatic	NR	Median 22.4 (IQR: 12.3-33.8) months	NR	NR	Abemaciclib better: 0.52 (0.37-0.73); P<0.001	NR	NR	NR	NR
	Palbociclib + ET	De novo metastatic	NR	45.7 (IQR: 28.0-59.6) months	NR	NR		NR	NR	NR	NR
PALMARES-2 655-Vernieri-2025; Italy	Palbociclib + ET	ILC, IPTW	NR	Median 29.8 months	NR	NR	Palbociclib better: 1.13 (0.75-1.71); P=0.551	NR	NR	NR	NR
	Abemaciclib + ET	ILC, IPTW	NR		NR	NR		NR	NR	NR	NR
	Palbociclib + ET	ILC, Cox regression	NR		NR	NR	Palbociclib better: 1.16 (0.86-1.70); P=0.447	NR	NR	NR	NR
	Abemaciclib + ET	ILC, Cox regression	NR		NR	NR		NR	NR	NR	NR
	Palbociclib + ET	Non-ILC, IPTW	NR	Median 31.5 months	NR	NR	Abemaciclib better: 0.70 (0.55-0.88); P=0.002	NR	NR	NR	NR
	Abemaciclib + ET	Non-ILC, IPTW	NR		NR	NR		NR	NR	NR	NR
	Palbociclib + ET	Non-ILC, Cox regression	NR		NR	NR	Abemaciclib better:	NR	NR	NR	NR

Study Name; Reference	Treatment	Subgroup	Sample size	Follow-up Time	Starting dose for CDK4/6i , n (%)	PFS			OS		
						Median (95% CI), months	HR (95% CI); P value	At latest timepoint , n (%)	Median (95% CI), months	HR (95% CI); P value	At latest timepoint, n (%)
	Abemaciclib + ET	Non-ILC, Cox regression	NR		NR	NR	0.64 (0.52-0.80); P<0.001	NR	NR	NR	NR
P-VERIFY 1132-Rugo-2025; United States	Abemaciclib + AI	sIPTW - Age 18-49 years	118	NR	NR	NR	Abemaciclib better: 0.78 (0.58-1.05); NR	NR	NR	NR	NR
	Palbociclib + AI	sIPTW - Age 18-49 years	775	NR	NR	NR		NR	NR	NR	NR
	Abemaciclib + AI	sIPTW - Age 50-64 years	359	NR	NR	NR	Abemaciclib similar: 0.99 (0.83-1.17); NR	NR	NR	NR	NR
	Palbociclib + AI	sIPTW - Age 50-64 years	2333	NR	NR	NR		NR	NR	NR	NR
	Abemaciclib + AI	sIPTW - Age 65-74 years	316	NR	NR	NR	Abemaciclib better: 0.86 (0.71-1.04); NR	NR	NR	NR	NR
	Palbociclib + AI	sIPTW - Age 65-74 years	2108	NR	NR	NR		NR	NR	NR	NR
	Abemaciclib + AI	sIPTW - Age ≥ 75 years	246	NR	NR	NR	Palbociclib better: 1.15 (0.94-1.41); NR	NR	NR	NR	NR
	Palbociclib + AI	sIPTW - Age ≥ 75 years	1616	NR	NR	NR		NR	NR	NR	NR
	Abemaciclib + AI	sIPTW - Race, White	654	NR	NR	NR	Abemaciclib better: 0.88 (0.76-1.00); NR	NR	NR	NR	NR
	Palbociclib + AI	sIPTW - Race, White	4272	NR	NR	NR		NR	NR	NR	NR

Study Name; Reference	Treatment	Subgroup	Sample size	Follow-up Time	Starting dose for CDK4/6i, n (%)	PFS			OS		
						Median (95% CI), months	HR (95% CI); P value	At latest timepoint, n (%)	Median (95% CI), months	HR (95% CI); P value	At latest timepoint, n (%)
	Abemaciclib + AI	sIPTW - Race, Black	94	NR	NR	NR	Palbociclib better: 1.17 (0.87-1.57); NR	NR	NR	NR	NR
	Palbociclib + AI	sIPTW - Race, Black	638	NR	NR	NR		NR	NR	NR	NR
	Abemaciclib + AI	sIPTW - Race, other	290	NR	NR	NR	Palbociclib better: 1.09 (0.92-1.29); NR	NR	NR	NR	NR
	Palbociclib + AI	sIPTW - Race, other	1922	NR	NR	NR		NR	NR	NR	NR
	Abemaciclib + AI	sIPTW - ECOG PS at baseline, 0	369	NR	NR	NR	Abemaciclib better: 0.79 (0.67-0.93); NR	NR	NR	NR	NR
	Palbociclib + AI	sIPTW - ECOG PS at baseline, 0	2444	NR	NR	NR		NR	NR	NR	NR
	Abemaciclib + AI	sIPTW - ECOG PS at baseline, 1	275	NR	NR	NR	Palbociclib better: 1.19 (0.99-1.42); NR	NR	NR	NR	NR
	Palbociclib + AI	sIPTW - ECOG PS at baseline, 1	1806	NR	NR	NR		NR	NR	NR	NR
	Abemaciclib + AI	sIPTW - ECOG PS at baseline, 2-4	119	NR	NR	NR	Palbociclib better: 1.09 (0.81-1.45); NR	NR	NR	NR	NR

Study Name; Reference	Treatment	Subgroup	Sample size	Follow-up Time	Starting dose for CDK4/6i, n (%)	PFS			OS		
						Median (95% CI), months	HR (95% CI); P value	At latest timepoint, n (%)	Median (95% CI), months	HR (95% CI); P value	At latest timepoint, n (%)
	Palbociclib + AI	sIPTW - ECOG PS at baseline, 2-4	780	NR	NR	NR		NR	NR	NR	NR
	Abemaciclib + AI	sIPTW - ECOG PS at baseline, ND	275	NR	NR	NR	Abemaciclib better: 0.94 (0.76-1.18); NR	NR	NR	NR	NR
	Palbociclib + AI	sIPTW - ECOG PS at baseline, ND	1801	NR	NR	NR		NR	NR	NR	NR
	Abemaciclib + AI	sIPTW - De novo metastatic	523	NR	NR	NR	Palbociclib better: 1.04 (0.91-1.20); NR	NR	NR	NR	NR
	Palbociclib + AI	sIPTW - De novo metastatic	3473	NR	NR	NR		NR	NR	NR	NR
	Abemaciclib + AI	sIPTW - No visceral disease	680	NR	NR	NR	Abemaciclib better: 0.90 (0.79-1.03); NR	NR	NR	NR	NR
	Palbociclib + AI	sIPTW - No visceral disease	4458	NR	NR	NR		NR	NR	NR	NR
	Abemaciclib + AI	sIPTW - Visceral disease	358	NR	NR	NR	Palbociclib better: 1.07 (0.91-1.25); NR	NR	NR	NR	NR
	Palbociclib + AI	sIPTW - Visceral disease	2374	NR	NR	NR		NR	NR	NR	NR
	Abemaciclib + AI	sIPTW - No bone-only disease	557	NR	NR	NR	Palbociclib better:	NR	NR	NR	NR

Study Name; Reference	Treatment	Subgroup	Sample size	Follow-up Time	Starting dose for CDK4/6i, n (%)	PFS			OS		
						Median (95% CI), months	HR (95% CI); P value	At latest timepoint, n (%)	Median (95% CI), months	HR (95% CI); P value	At latest timepoint, n (%)
	Palbociclib + AI	sIPTW - No bone-only disease	3661	NR	NR	NR	1.02 (0.89-1.16); NR	NR	NR	NR	NR
	Abemaciclib + AI	sIPTW - Bone-only disease	481	NR	NR	NR	Abemaciclib better: 0.88 (0.75-1.03); NR	NR	NR	NR	NR
	Palbociclib + AI	sIPTW - Bone-only disease	3171	NR	NR	NR		NR	NR	NR	NR
	Abemaciclib + AI	sIPTW - Metastatic sites, 1	611	NR	NR	NR	Abemaciclib better: 0.95 (0.83-1.10); NR	NR	NR	NR	NR
	Palbociclib + AI	sIPTW - Metastatic sites, 1	4010	NR	NR	NR		NR	NR	NR	NR
	Abemaciclib + AI	sIPTW - Metastatic sites, 2	240	NR	NR	NR	Abemaciclib better: 0.97 (0.80-1.17); NR	NR	NR	NR	NR
	Palbociclib + AI	sIPTW - Metastatic sites, 2	1585	NR	NR	NR		NR	NR	NR	NR
	Abemaciclib + AI	sIPTW - Metastatic sites, ≥ 3	92	NR	NR	NR	Palbociclib better: 1.14 (0.86-1.50); NR	NR	NR	NR	NR
	Palbociclib + AI	sIPTW - Metastatic sites, ≥ 3	615	NR	NR	NR		NR	NR	NR	NR
	Abemaciclib + AI	sIPTW - Metastatic sites, ND	95	NR	NR	NR	Abemaciclib better: 0.77 (0.52-1.15); NR	NR	NR	NR	NR
	Palbociclib + AI	sIPTW - Metastatic sites, ND	622	NR	NR	NR		NR	NR	NR	NR

Study Name; Reference	Treatment	Subgroup	Sample size	Follow-up Time	Starting dose for CDK4/6i , n (%)	PFS			OS		
						Median (95% CI), months	HR (95% CI); P value	At latest timepoint , n (%)	Median (95% CI), months	HR (95% CI); P value	At latest timepoin t, n (%)
P-VERIFY 563-Rugo-2025; United States	Abemaciclib + AI	sIPTW - Age 18-49 years	118	NR	NR	NR	NR	NR	NR	Abemaciclib better: 0.68 (0.46-0.99); NR	NR
	Palbociclib + AI	sIPTW - Age 18-49 years	775	NR	NR	NR	NR	NR	NR		NR
	Abemaciclib + AI	sIPTW - Age 50-64 years	359	NR	NR	NR	NR	NR	NR	Abemaciclib better: 0.94 (0.76-1.17); NR	NR
	Palbociclib + AI	sIPTW - Age 50-64 years	2333	NR	NR	NR	NR	NR	NR		NR
	Abemaciclib + AI	sIPTW - Age 65-74 years	316	NR	NR	NR	NR	NR	NR	Abemaciclib better: 0.90 (0.70-1.14); NR	NR
	Palbociclib + AI	sIPTW - Age 65-74 years	2108	NR	NR	NR	NR	NR	NR		NR
	Abemaciclib + AI	sIPTW - Age ≥ 75 years	246	NR	NR	NR	NR	NR	NR	Palbociclib better: 1.14 (0.89-1.45); NR	NR
	Palbociclib + AI	sIPTW - Age ≥ 75 years	1616	NR	NR	NR	NR	NR	NR		NR
	Abemaciclib + AI	sIPTW - Race, White	654	NR	NR	NR	NR	NR	NR	Abemaciclib better: 0.88 (0.74-1.05); NR	NR
	Palbociclib + AI	sIPTW - Race, White	4272	NR	NR	NR	NR	NR	NR		NR
	Abemaciclib + AI	sIPTW - Race, Black	94	NR	NR	NR	NR	NR	NR	Palbociclib better:	NR

Study Name; Reference	Treatment	Subgroup	Sample size	Follow-up Time	Starting dose for CDK4/6i, n (%)	PFS			OS		
						Median (95% CI), months	HR (95% CI); P value	At latest timepoint, n (%)	Median (95% CI), months	HR (95% CI); P value	At latest timepoint, n (%)
	Palbociclib + AI	sIPTW - Race, Black	638	NR	NR	NR	NR	NR	NR	1.29 (0.92-1.81); NR	NR
	Abemaciclib + AI	sIPTW - Race, other	290	NR	NR	NR	NR	NR	NR	Abemaciclib similar: 1.00 (0.80-1.25); NR	NR
	Palbociclib + AI	sIPTW - Race, other	1922	NR	NR	NR	NR	NR	NR		NR
	Abemaciclib + AI	sIPTW - ECOG PS at baseline, 0	369	NR	NR	NR	NR	NR	NR	Abemaciclib better: 0.61 (0.48-0.78); NR	NR
	Palbociclib + AI	sIPTW - ECOG PS at baseline, 0	2444	NR	NR	NR	NR	NR	NR		NR
	Abemaciclib + AI	sIPTW - ECOG PS at baseline, 1	275	NR	NR	NR	NR	NR	NR	Palbociclib better: 1.22 (0.96-1.54); NR	NR
	Palbociclib + AI	sIPTW - ECOG PS at baseline, 1	1806	NR	NR	NR	NR	NR	NR		NR
	Abemaciclib + AI	sIPTW - ECOG PS at baseline, 2-4	119	NR	NR	NR	NR	NR	NR	Palbociclib better: 1.15 (0.81-1.62); NR	NR
	Palbociclib + AI	sIPTW - ECOG PS at baseline, 2-4	780	NR	NR	NR	NR	NR	NR		NR

Study Name; Reference	Treatment	Subgroup	Sample size	Follow-up Time	Starting dose for CDK4/6i, n (%)	PFS			OS		
						Median (95% CI), months	HR (95% CI); P value	At latest timepoint, n (%)	Median (95% CI), months	HR (95% CI); P value	At latest timepoint, n (%)
	Abemaciclib + AI	sIPTW - ECOG PS at baseline, ND	275	NR	NR	NR	NR	NR	NR	Palbociclib better: 1.03 (0.80-1.32); NR	NR
	Palbociclib + AI	sIPTW - ECOG PS at baseline, ND	1801	NR	NR	NR	NR	NR	NR		NR
	Abemaciclib + AI	sIPTW - De novo metastatic	523	NR	NR	NR	NR	NR	NR	Palbociclib better: 1.06 (0.90-1.24); NR	NR
	Palbociclib + AI	sIPTW - De novo metastatic	3473	NR	NR	NR	NR	NR	NR		NR
	Abemaciclib + AI	sIPTW - No visceral disease	680	NR	NR	NR	NR	NR	NR	Abemaciclib better: 0.88 (0.74-1.05); NR	NR
	Palbociclib + AI	sIPTW - No visceral disease	4458	NR	NR	NR	NR	NR	NR		NR
	Abemaciclib + AI	sIPTW - Visceral disease	358	NR	NR	NR	NR	NR	NR	Palbociclib better: 1.07 (0.88-1.29); NR	NR
	Palbociclib + AI	sIPTW - Visceral disease	2374	NR	NR	NR	NR	NR	NR		NR
	Abemaciclib + AI	sIPTW - No bone-only disease	557	NR	NR	NR	NR	NR	NR	Abemaciclib better: 0.96 (0.81-1.13); NR	NR
	Palbociclib + AI	sIPTW - No bone-only disease	3661	NR	NR	NR	NR	NR	NR		NR

Study Name; Reference	Treatment	Subgroup	Sample size	Follow-up Time	Starting dose for CDK4/6i, n (%)	PFS			OS		
						Median (95% CI), months	HR (95% CI); P value	At latest timepoint, n (%)	Median (95% CI), months	HR (95% CI); P value	At latest timepoint, n (%)
	Abemaciclib + AI	sIPTW - Bone-only disease	481	NR	NR	NR	NR	NR	NR	Abemaciclib better: 0.93 (0.76-1.14); NR	NR
	Palbociclib + AI	sIPTW - Bone-only disease	3171	NR	NR	NR	NR	NR	NR		NR
	Abemaciclib + AI	sIPTW - Metastatic sites, 1	611	NR	NR	NR	NR	NR	NR	Abemaciclib better: 0.92 (0.77-1.11); NR	NR
	Palbociclib + AI	sIPTW - Metastatic sites, 1	4010	NR	NR	NR	NR	NR	NR		NR
	Abemaciclib + AI	sIPTW - Metastatic sites, 2	240	NR	NR	NR	NR	NR	NR	Abemaciclib better: 0.98 (0.78-1.24); NR	NR
	Palbociclib + AI	sIPTW - Metastatic sites, 2	1585	NR	NR	NR	NR	NR	NR		NR
	Abemaciclib + AI	sIPTW - Metastatic sites, ≥ 3	92	NR	NR	NR	NR	NR	NR	Palbociclib better: 1.16 (0.83-1.63); NR	NR
	Palbociclib + AI	sIPTW - Metastatic sites, ≥ 3	615	NR	NR	NR	NR	NR	NR		NR
	Abemaciclib + AI	sIPTW - Metastatic sites, ND	95	NR	NR	NR	NR	NR	NR	Abemaciclib better: 0.73 (0.44-1.22); NR	NR
	Palbociclib + AI	sIPTW - Metastatic sites, ND	622	NR	NR	NR	NR	NR	NR		NR
P-VERIFY	Palbociclib + AI	sIPTW - Age 18-49 years	775	NR	NR	NR	NR	NR	NR	Abemaciclib better:	NR

Study Name; Reference	Treatment	Subgroup	Sample size	Follow-up Time	Starting dose for CDK4/6i, n (%)	PFS			OS		
						Median (95% CI), months	HR (95% CI); P value	At latest timepoint, n (%)	Median (95% CI), months	HR (95% CI); P value	At latest timepoint, n (%)
SABS24-102-Rugo-2024[a]; United States	Abemaciclib + AI	sIPTW - Age 18-49 years	118	NR	NR	NR	NR	NR	NR	0.68 (0.46-0.99); NR	NR
	Palbociclib + AI	sIPTW - Age 50-64 years	2333	NR	NR	NR	NR	NR	NR	Abemaciclib better: 0.94 (0.76-1.17); NR	NR
	Abemaciclib + AI	sIPTW - Age 50-64 years	359	NR	NR	NR	NR	NR	NR		NR
	Palbociclib + AI	sIPTW - Age 65-75 years	2108	NR	NR	NR	NR	NR	NR	Abemaciclib better: 0.90 (0.70-1.14); NR	NR
	Abemaciclib + AI	sIPTW - Age 65-75 years	316	NR	NR	NR	NR	NR	NR		NR
	Palbociclib + AI	sIPTW - Age ≥ 75 years	1616	NR	NR	NR	NR	NR	NR	Palbociclib better: 1.14 (0.89-1.45); NR	NR
	Abemaciclib + AI	sIPTW - Age ≥ 75 years	246	NR	NR	NR	NR	NR	NR		NR
	Palbociclib + AI	sIPTW - Race, White	4272	NR	NR	NR	NR	NR	NR	Abemaciclib better: 0.88 (0.74-1.05); NR	NR
	Abemaciclib + AI	sIPTW - Race, White	654	NR	NR	NR	NR	NR	NR		NR
	Palbociclib + AI	sIPTW - Race, Black	638	NR	NR	NR	NR	NR	NR	Palbociclib better: 1.29 (0.92-1.81); NR	NR
	Abemaciclib + AI	sIPTW - Race, Black	94	NR	NR	NR	NR	NR	NR		NR

Study Name; Reference	Treatment	Subgroup	Sample size	Follow-up Time	Starting dose for CDK4/6i, n (%)	PFS			OS		
						Median (95% CI), months	HR (95% CI); P value	At latest timepoint, n (%)	Median (95% CI), months	HR (95% CI); P value	At latest timepoint, n (%)
	Palbociclib + AI	sIPTW - Race, other	1922	NR	NR	NR	NR	NR	NR	Abemaciclib similar: 1.00 (0.80-1.25); NR	NR
	Abemaciclib + AI	sIPTW - Race, other	290	NR	NR	NR	NR	NR	NR		NR
	Palbociclib + AI	sIPTW - ECOG PS at baseline, 0	2444	NR	NR	NR	NR	NR	NR	Abemaciclib better: 0.61 (0.48-0.78); NR	NR
	Abemaciclib + AI	sIPTW - ECOG PS at baseline, 0	369	NR	NR	NR	NR	NR	NR		NR
	Palbociclib + AI	sIPTW - ECOG PS at baseline, 1	1806	NR	NR	NR	NR	NR	NR	Palbociclib better: 1.22 (0.96-1.54); NR	NR
	Abemaciclib + AI	sIPTW - ECOG PS at baseline, 1	275	NR	NR	NR	NR	NR	NR		NR
	Palbociclib + AI	sIPTW - ECOG PS at baseline, 2 -4	780	NR	NR	NR	NR	NR	NR	Palbociclib better: 1.15 (0.81-1.62); NR	NR
	Abemaciclib + AI	sIPTW - ECOG PS at baseline, 2 -4	119	NR	NR	NR	NR	NR	NR		NR
	Palbociclib + AI	sIPTW - ECOG PS at baseline, ND	1801	NR	NR	NR	NR	NR	NR	Palbociclib better: 1.03 (0.80-1.32); NR	NR

Study Name; Reference	Treatment	Subgroup	Sample size	Follow-up Time	Starting dose for CDK4/6i, n (%)	PFS			OS		
						Median (95% CI), months	HR (95% CI); P value	At latest timepoint, n (%)	Median (95% CI), months	HR (95% CI); P value	At latest timepoint, n (%)
	Abemaciclib + AI	sIPTW - ECOG PS at baseline, ND	275	NR	NR	NR	NR	NR	NR		NR
	Palbociclib + AI	sIPTW - De novo metastatic	3473	NR	NR	NR	NR	NR	NR	Palbociclib better: 1.06 (0.90-1.24); NR	NR
	Abemaciclib + AI	sIPTW - De novo metastatic	523	NR	NR	NR	NR	NR	NR		NR
	Palbociclib + AI	sIPTW - No visceral disease	4458	NR	NR	NR	NR	NR	NR	Abemaciclib better: 0.88 (0.74-1.05); NR	NR
	Abemaciclib + AI	sIPTW - No visceral disease	680	NR	NR	NR	NR	NR	NR		NR
	Palbociclib + AI	sIPTW - Visceral disease	2374	NR	NR	NR	NR	NR	NR	Palbociclib better: 1.07 (0.88-1.29); NR	NR
	Abemaciclib + AI	sIPTW - Visceral disease	358	NR	NR	NR	NR	NR	NR		NR
	Palbociclib + AI	sIPTW - No bone-only disease	3661	NR	NR	NR	NR	NR	NR	Abemaciclib better: 0.96 (0.81-1.13); NR	NR
	Abemaciclib + AI	sIPTW - No bone-only disease	557	NR	NR	NR	NR	NR	NR		NR
	Palbociclib + AI	sIPTW - Bone only disease	3171	NR	NR	NR	NR	NR	NR	Abemaciclib better:	NR

Study Name; Reference	Treatment	Subgroup	Sample size	Follow-up Time	Starting dose for CDK4/6i, n (%)	PFS			OS		
						Median (95% CI), months	HR (95% CI); P value	At latest timepoint, n (%)	Median (95% CI), months	HR (95% CI); P value	At latest timepoint, n (%)
	Abemaciclib + AI	sIPTW - Bone only disease	481	NR	NR	NR	NR	NR	NR	0.93 (0.76-1.14); NR	NR
	Palbociclib + AI	sIPTW - Metastatic sites, 1	4010	NR	NR	NR	NR	NR	NR	Abemaciclib better: 0.92 (0.77-1.11); NR	NR
	Abemaciclib + AI	sIPTW - Metastatic sites, 1	611	NR	NR	NR	NR	NR	NR		NR
	Palbociclib + AI	sIPTW - metastatic sites, 2	1585	NR	NR	NR	NR	NR	NR	Abemaciclib better: 0.98 (0.78-1.24); NR	NR
	Abemaciclib + AI	sIPTW - metastatic sites, 2	240	NR	NR	NR	NR	NR	NR		NR
	Palbociclib + AI	sIPTW - metastatic sites, ≥3	615	NR	NR	NR	NR	NR	NR	Palbociclib better: 1.16 (0.83-1.63); NR	NR
	Abemaciclib + AI	sIPTW - metastatic sites, ≥3	92	NR	NR	NR	NR	NR	NR		NR
	Palbociclib + AI	sIPTW - metastatic sites, NS	622	NR	NR	NR	NR	NR	NR	Abemaciclib better: 0.73 (0.44-1.22); NR	NR
	Abemaciclib + AI	sIPTW - metastatic sites, NS	95	NR	NR	NR	NR	NR	NR		NR

Note: For records with a high volume of subgroup data, subgroups outlined in Appendix F were prioritized.
Note: The “Study Name; Reference” cells shaded in grey highlight full text records, while non-shaded are abstracts/manuscripts.
Note: Overall follow-up time was used where subgroup-specific follow-up times were not reported.
[a] Different data reported in the poster and the abstract.
Abbreviations: AI = aromatase inhibitor; CDK4/6i = cyclin-dependent kinase 4/6 inhibitors; CI = confidence interval; ECOG PS = eastern cooperative oncology group performance status; ER = estrogen receptor; ET = endocrine therapy; HR = hazard ratio; ILC = invasive lobular

carcinoma; IQR = interquartile range; IPTW = inverse probability of treatment weighting; NA = not available; ND = not documented; NR = not reported; NS = not specified; OS = overall survival; PFS = progression-free survival; PR = progesterone receptor; RWE = real-world evidence; sIPTW = stabilized inverse probability treatment weighting.

Table S8. Progression-free survival and overall survival for first-line ribociclib in comparative RWE studies versus abemaciclib (overall population; studies with manuscript data available)

Study Name; Reference	Treatment	Subgroup	Sample size	Follow-up Time	Starting dose for CDK4/6i, n (%)	PFS			OS		
						Median (95% CI), months	HR (95% CI); P value	At latest timepoint, n (%)	Median (95% CI), months	HR (95% CI); P value	At latest timepoint, n (%)
4112-Cejuela-2023; Spain	Abemaciclib + ET	All patients	56	Median 40.28 months	NR	39.49 (NR)	NR	NR	Not reached	NR	NR
	Ribociclib + ET	All patients	54	Median 64.4 months	NR	31.14 (NR)	NR	NR	Not reached	NR	NR
4132-Buller-2023; United States	Ribociclib + ET	All patients	28	NR	NR	31.10 (NR)	NR	NR	NR	NR	NR
	Abemaciclib + ET	All patients	44	NR	NR	17.00 (10.41-23.59)	NR	NR	34.30 (NR)	NR	NR
4506-Tang-2023; United Kingdom	Ribociclib + ET	All patients	46	Median 49.5 months	NR	25.7 (NR)	NR	5 years: NR (32.58)	50.2 (NR)	NR	5 years: NR (42.33)
	Abemaciclib + ET	All patients	19		NR	Not reached	NR	5 years: NR (66.8)	Not reached	NR	5 years: NR (66.9)
6590-Gehrchen-2024; Denmark	Abemaciclib + ET	All patients	322	Median 24.8 (95%CI: 24.1-25.8) months	NR	Not reached	NR	24 months: NR (65)	37.8 (32.5-NA)	NR	NR
	Ribociclib+ ET	All patients	359	Median 47.9 (95%CI: 45.8-49.7) months	NR	42.4 (35.1-52.9)	NR	24 months: NR (66)	54.4 (47.9-NA)	NR	NR

Study Name; Reference	Treatment	Subgroup	Sample size	Follow-up Time	Starting dose for CDK4/6i, n (%)	PFS			OS		
						Median (95% CI), months	HR (95% CI); P value	At latest timepoint, n (%)	Median (95% CI), months	HR (95% CI); P value	At latest timepoint, n (%)
CDK-PREDICT study 633-Tolosa-2025; Spain	Ribociclib + ET	All patients	82	Median 38.5 (IQR: 26.5-53.8) months	NR	22.9 (17.2-29.2)	NR	NR	55.4 (42.8-75.1)	NR	NR
	Abemaciclib + ET	All patients	17		NR	12.4 (8.0-not reached)	NR	NR	46.9 (22.5-not reached)	NR	NR
PALMARES-2 540-Provenzano-2025[a]; Italy	Abemaciclib + ET	IPTW-adjusted analyses	NR	Median 22.4 (IQR: 12.3-33.8) months	NR	46.6 (33.6-NA)	Abemaciclib better: 0.94 (0.76-1.17); P=0.571	NR	NR	NR	NR
	Ribociclib + ET	IPTW-adjusted analyses	NR	Median 25.2 (IQR: 12.7-44.4) months	NR	37.4 (32.0-47.2)		NR	NR	NR	NR
PALMARES-2 883-Vernieri-2025; Italy	Ribociclib + ET	IPTW	1408	Median 31.8 months	NR	NR	Abemaciclib similar: 0.99 (0.87-1.13); P=0.91	NR	NR	Ribociclib better: 1.21 (0.99-1.49); P=0.06	NR
	Abemaciclib + ET	IPTW	798	Median 29.6 months	NR	NR		NR	NR		NR
PALMARES-2; 5854-Vernieri-2024a; Italy	Ribociclib + ET	All patients	676	NR	NR	NR	Abemaciclib better: 0.91 (0.70-1.19); P = 0.505	NR	NR	NR	NR
	Abemaciclib + ET	All patients	424	NR	NR	NR		NR	NR	NR	NR
P-VERIFY 1132-Rugo-2025[a]; United States	Abemaciclib + AI	After sIPTW adjustment	1038	Median 21.5 (IQR:	NR	22.9 (20.2-26.5)	Abemaciclib better:	NR	NR	NR	NR

Study Name; Reference	Treatment	Subgroup	Sample size	Follow-up Time	Starting dose for CDK4/6i, n (%)	PFS			OS		
						Median (95% CI), months	HR (95% CI); P value	At latest timepoint, n (%)	Median (95% CI), months	HR (95% CI); P value	At latest timepoint, n (%)
				25.0) months			0.98 (0.86-1.12); P=0.8024				
	Ribociclib + AI	After sIPTW adjustment	1274	Median 15.7 (IQR: 20.8) months	NR	22.9 (21.0-25.6)		NR	NR	NR	NR
P-VERIFY 563-Rugo-2025[a]; United States	Abemaciclib + AI	After sIPTW adjustment	1038	Median 21.5 (IQR: 25.0) months	NR	NR	NR	NR	64.5 (55.4-NE)	Abemaciclib better: 0.97 (0.82-1.14); P=0.6956	30 months: NR (71.5)
	Ribociclib + AI	After sIPTW adjustment	1274	Median 15.7 (IQR: 20.8) months	NR	NR	NR	NR	59.0 (50.9-66.1)		30 months: NR (72.2)
P-VERIFY SABS24-102-Rugo-2024[a,b]; United States	Ribociclib + AI	sIPTW	1274	Median 15.7 (IQR: 20.8) months	NR	NR	NR	NR	59.0 (50.9-66.1)	Abemaciclib better: 0.97 (0.82-1.14); P = 0.6956	30 months: NR (72.2)
	Abemaciclib + AI	sIPTW	1038	Median 21.5 (IQR: 25.0) months	NR	NR	NR	NR	64.5 (55.4-NE)		30 month: NR (71.5)

Note: The “Study Name; Reference” cells shaded in grey highlight full text records, while non-shaded are abstracts/manuscripts of the parent study.
[a] Record also reports unadjusted data.
[b] Different data reported in the poster and the abstract.
Abbreviations: AI = aromatase inhibitor; CDK4/6i = cyclin-dependent kinase 4/6 inhibitors; CI = confidence interval; ET = endocrine therapy; HR = hazard ratio; IQR = interquartile range; IPTW = inverse probability of treatment weighting; NA = not available; NE = not estimable; NR = not reported; OS = overall survival; PFS = progression-free survival; RWE = real-world evidence; sIPTW = stabilized inverse probability treatment weighting.

Table S9. Progression-free survival and overall survival for first-line ribociclib in comparative RWE studies versus abemaciclib (subgroups; studies with manuscript data available)

Study Name; Reference	Treatment	Subgroup	Sample size	Follow-up Time	Starting dose for CDK4/6i, n (%)	PFS			OS		
						Median (95% CI), months	HR (95% CI); P value	At latest timepoint, n (%)	Median (95% CI), months	HR (95% CI); P value	At latest timepoint, n (%)
4112-Cejuela-2023; Spain	Abemaciclib + ET	Endocrine sensitive	35	NR	NR	NR	Abemaciclib better: 2.19 (0.91-5.31); P=0.091	NR	NR	NR	NR
	Ribociclib + ET	Endocrine sensitive	34	NR	NR	NR		NR	NR	NR	NR
	Abemaciclib + ET	Endocrine resistant	21	NR	NR	Not reached	NR; P=0.07	NR	NR	NR	NR
	Ribociclib + ET	Endocrine resistant	20	NR	NR	10.38 (NR)		NR	NR	NR	NR
	Abemaciclib + ET	Visceral disease	34	NR	NR	39.49 (NR)	NR; P=0.307	NR	NR	NR	NR
	Ribociclib + ET	Visceral disease	32	NR	NR	23.16 (NR)		NR	NR	NR	NR
	Abemaciclib + ET	Non-visceral disease	22	NR	NR	Not reached	NR; P=0.038	NR	NR	NR	NR
	Ribociclib + ET	Non-visceral disease	22	NR	NR	36.01 (NR)		NR	NR	NR	NR
4506-Tang-2023; United Kingdom	Ribociclib + ET	De novo	12	NR	NR	Not reached	NR	5 years: NR (74.07)	Not reached	NR	5 years: NR (82.5)
	Abemaciclib + ET	De novo	11	NR	NR	Not reached	NR	NR	Not reached	NR	NR

Study Name; Reference	Treatment	Subgroup	Sample size	Follow-up Time	Starting dose for CDK4/6i, n (%)	PFS			OS		
						Median (95% CI), months	HR (95% CI); P value	At latest timepoint, n (%)	Median (95% CI), months	HR (95% CI); P value	At latest timepoint, n (%)
	Ribociclib + ET	Recurrent	34	NR	NR	18.85 (NR)	NR	5 years: NR (19.1)	44.6 (NR)	NR	5 years: NR (27.98)
	Abemaciclib + ET	Recurrent	8	NR	NR	Not reached	NR	NR	30.7 (NR)	NR	NR
	Ribociclib + ET	ER+/PR+	31	NR	NR	44 (NR)	NR	5 years: NR (41.82)	54.8 (NR)	NR	5 years: NR (45.37)
	Abemaciclib + ET	ER+/PR+	15	NR	NR	Not reached	NR	NR	Not reached	NR	NR
	Ribociclib + ET	ER+/PR-	11	NR	NR	10.1 (NR)	NR	5 years: NR (13.33)	34.3 (NR)	NR	5 years: NR (40)
	Abemaciclib + ET	ER+/PR-	4	NR	NR	28.3 (NR)	NR	NR	30.2 (NR)	NR	NR
	Ribociclib + ET	≤65 years	25	NR	NR	20.5 (NR)	NR	5 years: NR (27.93)	44.6 (NR)	NR	5 years: NR (36.86)
	Abemaciclib + ET	≤65 years	8	NR	NR	Not reached	NR	NR	36.5 (NR)	NR	NR
	Ribociclib + ET	66-79 years	16	NR	NR	24.7 (NR)	NR	5 years: NR (34.29)	54.8 (NR)	NR	5 years: NR (49.36)
	Abemaciclib + ET	66-79 years	6	NR	NR	Not reached	NR	NR	Not reached	NR	NR
	Ribociclib + ET	≥80 years	5	NR	NR	68.2 (NR)	NR	5 years: NR (60)	Not reached	NR	5 years: NR (60)

Study Name; Reference	Treatment	Subgroup	Sample size	Follow-up Time	Starting dose for CDK4/6i , n (%)	PFS			OS		
						Median (95% CI), months	HR (95% CI); P value	At latest timepoint, n (%)	Median (95% CI), months	HR (95% CI); P value	At latest timepoint, n (%)
	Abemaciclib + ET	≥80 years	5	NR	NR	Not reached	NR	NR	Not reached	NR	NR
5893-Skocilic-2024; Croatia	Abemaciclib + ET	Progesterone <10%	NR	Median: 3.9 months	NR	NR	NR	NR	3.9 (1.4-3.9)	NR	NR
	Ribociclib + ET		NR	Median: 3.7 months	NR	NR	NR	NR	3.7 (1.5-3.8)	NR	NR
	Abemaciclib + ET	Liver metastasis	NR	Median: 3.9 months	NR	NR	NR	NR	3.9 (2.8-not reached)	NR	NR
	Ribociclib + ET		NR	Median: 1.6 months	NR	NR	NR	NR	1.6 (0.7-1.6)	NR	NR
PALMARES-2 540-Provenzano-2025; Italy	Abemaciclib + ET	Endocrine sensitive	NR	Median 22.4 (IQR: 12.3-33.8) months	NR	NR	Abemaciclib better:<br><br>0.85 (0.59-1.24); P=0.401	NR	NR	NR	NR
	Ribociclib + ET	Endocrine sensitive	NR	Median 25.2 (IQR: 12.7-44.4) months	NR	NR		NR	NR	NR	NR
	Abemaciclib + ET	Endocrine resistant	NR	Median 22.4 (IQR: 12.3-33.8) months	NR	NR	Ribociclib better:<br><br>1.02 (0.95-1.09); P=0.624	NR	NR	NR	NR

Study Name; Reference	Treatment	Subgroup	Sample size	Follow-up Time	Starting dose for CDK4/6i, n (%)	PFS			OS		
						Median (95% CI), months	HR (95% CI); P value	At latest timepoint, n (%)	Median (95% CI), months	HR (95% CI); P value	At latest timepoint, n (%)
	Ribociclib + ET	Endocrine resistant	NR	Median 25.2 (IQR: 12.7-44.4) months	NR	NR		NR	NR	NR	NR
	Abemaciclib + ET	Luminal B-like	NR	Median 22.4 (IQR: 12.3-33.8) months	NR	NR	Abemaciclib better: 0.94 (0.75-1.18); P=0.324	NR	NR	NR	NR
	Ribociclib + ET	Luminal B-like	NR	Median 25.2 (IQR: 12.7-44.4) months	NR	NR		NR	NR	NR	NR
	Abemaciclib + ET	Liver metastasis	NR	Median 22.4 (IQR: 12.3-33.8) months	NR	NR	Abemaciclib better: 0.82 (0.47-1.44); P=0.488	NR	NR	NR	NR
	Ribociclib + ET	Liver metastasis	NR	Median 25.2 (IQR: 12.7-44.4) months	NR	NR		NR	NR	NR	NR
	Abemaciclib + ET	Premenopausal	NR	Median 22.4 (IQR: 12.3-	NR	NR	Ribociclib better:	NR	NR	NR	NR

Study Name; Reference	Treatment	Subgroup	Sample size	Follow-up Time	Starting dose for CDK4/6i, n (%)	PFS			OS		
						Median (95% CI), months	HR (95% CI); P value	At latest timepoint, n (%)	Median (95% CI), months	HR (95% CI); P value	At latest timepoint, n (%)
				33.8) months			1.05 (0.78-1.39); P=0.759				
	Ribociclib + ET	Premenopausal	NR	Median 25.2 (IQR: 12.7-44.4) months	NR	NR		NR	NR	NR	NR
	Abemaciclib + ET	Poor ECOG PS	NR	Median 22.4 (IQR: 12.3-33.8) months	NR	NR	Abemaciclib better: 0.90 (0.68-1.20); P=0.485	NR	NR	NR	NR
	Ribociclib + ET	Poor ECOG PS	NR	Median 25.2 (IQR: 12.7-44.4) months	NR	NR		NR	NR	NR	NR
	Abemaciclib + ET	Older	NR	Median 22.4 (IQR: 12.3-33.8) months	NR	NR	Abemacilcib better: 0.82 (0.64-1.05); P=0.120	NR	NR	NR	NR
	Ribociclib + ET	Older	NR	Median 25.2 (IQR: 12.7-44.4) months	NR	NR		NR	NR	NR	NR

Study Name; Reference	Treatment	Subgroup	Sample size	Follow-up Time	Starting dose for CDK4/6i, n (%)	PFS			OS		
						Median (95% CI), months	HR (95% CI); P value	At latest timepoint, n (%)	Median (95% CI), months	HR (95% CI); P value	At latest timepoint, n (%)
	Abemaciclib + ET	Bone only	NR	Median 22.4 (IQR: 12.3-33.8) months	NR	NR	Ribociclib better: 1.08 (0.58-1.99); P=0.810	NR	NR	NR	NR
	Ribociclib + ET	Bone only	NR	Median 25.2 (IQR: 12.7-44.4) months	NR	NR		NR	NR	NR	NR
	Abemaciclib + ET	De novo metastatic	NR	Median 22.4 (IQR: 12.3-33.8) months	NR	NR	Abemaciclib better: 0.69 (0.60-0.79); P<0.001	NR	NR	NR	NR
	Ribociclib + ET	De novo metastatic	NR	Median 25.2 (IQR: 12.7-44.4) months	NR	NR		NR	NR	NR	NR
PALMARES-2 655-Vernieri-2025; Italy	Ribociclib + ET	ILC, IPTW	NR	Median 29.8 months	NR	NR	Ribociclib better: 1.15 (0.73-1.80); P=0.549	NR	NR	NR	NR
	Abemaciclib + ET	ILC, IPTW	NR		NR	NR		NR	NR	NR	NR
	Ribociclib + ET	ILC, Cox regression	NR		NR	NR	Ribociclib better:	NR	NR	NR	NR

Study Name; Reference	Treatment	Subgroup	Sample size	Follow-up Time	Starting dose for CDK4/6i , n (%)	PFS			OS		
						Median (95% CI), months	HR (95% CI); P value	At latest timepoint, n (%)	Median (95% CI), months	HR (95% CI); P value	At latest timepoint, n (%)
	Abemaciclib + ET	ILC, Cox regression	NR		NR	NR	1.20 (0.79-1.88); P=0.393	NR	NR	NR	NR
	Ribociclib + ET	Non-ILC, IPTW	NR	Median 31.5 months	NR	NR	Abemaciclib better: 0.90 (0.70-1.15); P=0.388	NR	NR	NR	NR
	Abemaciclib + ET	Non-ILC, IPTW	NR		NR	NR		NR	NR	NR	NR
	Ribociclib + ET	Non-ILC, Cox regression	NR		NR	NR	Abemaciclib better: 0.81 (0.64-1.03); P=0.081	NR	NR	NR	NR
	Abemaciclib + ET	Non-ILC, Cox regression	NR		NR	NR		NR	NR	NR	NR
P-VERIFY 1132-Rugo-2025; United States	Abemaciclib + AI	sIPTW - Age 18-49 years	118	NR	NR	NR	Abemaciclib better: 0.77 (0.55-1.08); NR	NR	NR	NR	NR
	Ribociclib + AI	sIPTW - Age 18-49 years	148	NR	NR	NR		NR	NR	NR	NR
	Abemaciclib + AI	sIPTW - Age 50-64 years	359	NR	NR	NR	Ribociclib better: 1.03 (0.82-1.29); NR	NR	NR	NR	NR
	Ribociclib + AI	sIPTW - Age 50-64 years	437	NR	NR	NR		NR	NR	NR	NR
	Abemaciclib + AI	sIPTW - Age 65-74 years	316	NR	NR	NR	Abemaciclib better:	NR	NR	NR	NR

Study Name; Reference	Treatment	Subgroup	Sample size	Follow-up Time	Starting dose for CDK4/6i, n (%)	PFS			OS		
						Median (95% CI), months	HR (95% CI); P value	At latest timepoint, n (%)	Median (95% CI), months	HR (95% CI); P value	At latest timepoint, n (%)
	Ribociclib + AI	sIPTW - Age 65-74 years	390	NR	NR	NR	0.89 (0.69-1.15); NR	NR	NR	NR	NR
	Abemaciclib + AI	sIPTW - Age ≥ 75 years	246	NR	NR	NR	Ribociclib better: 1.17 (0.88-1.55); NR	NR	NR	NR	NR
	Ribociclib + AI	sIPTW - Age ≥ 75 years	299	NR	NR	NR		NR	NR	NR	NR
	Abemaciclib + AI	sIPTW - Race, White	654	NR	NR	NR	Abemaciclib better: 0.90 (0.75-1.07); NR	NR	NR	NR	NR
	Ribociclib + AI	sIPTW - Race, White	797	NR	NR	NR		NR	NR	NR	NR
	Abemaciclib + AI	sIPTW - Race, Black	94	NR	NR	NR	Ribociclib better: 1.25 (0.84-1.88); NR	NR	NR	NR	NR
	Ribociclib + AI	sIPTW - Race, Black	117	NR	NR	NR		NR	NR	NR	NR
	Abemaciclib + AI	sIPTW - Race, other	290	NR	NR	NR	Ribociclib better: 1.11 (0.88-1.39); NR	NR	NR	NR	NR
	Ribociclib + AI	sIPTW - Race, other	360	NR	NR	NR		NR	NR	NR	NR
	Abemaciclib + AI	sIPTW - ECOG PS at baseline, 0	369	NR	NR	NR	Abemaciclib better: 0.83 (0.66-1.03); NR	NR	NR	NR	NR

Study Name; Reference	Treatment	Subgroup	Sample size	Follow-up Time	Starting dose for CDK4/6i, n (%)	PFS			OS		
						Median (95% CI), months	HR (95% CI); P value	At latest timepoint, n (%)	Median (95% CI), months	HR (95% CI); P value	At latest timepoint, n (%)
	Ribociclib + AI	sIPTW - ECOG PS at baseline, 0	457	NR	NR	NR		NR	NR	NR	NR
	Abemaciclib + AI	sIPTW - ECOG PS at baseline, 1	275	NR	NR	NR	Ribociclib better: 1.23 (0.96-1.57); NR	NR	NR	NR	NR
	Ribociclib + AI	sIPTW - ECOG PS at baseline, 1	329	NR	NR	NR		NR	NR	NR	NR
	Abemaciclib + AI	sIPTW - ECOG PS at baseline, 2-4	119	NR	NR	NR	Abemaciclib better: 0.96 (0.67-1.39); NR	NR	NR	NR	NR
	Ribociclib + AI	sIPTW - ECOG PS at baseline, 2-4	147	NR	NR	NR		NR	NR	NR	NR
	Abemaciclib + AI	sIPTW - ECOG PS at baseline, ND	275	NR	NR	NR	Ribociclib better: 1.04 (0.77-1.39); NR	NR	NR	NR	NR
	Ribociclib + AI	sIPTW - ECOG PS at baseline, ND	341	NR	NR	NR		NR	NR	NR	NR
	Abemaciclib + AI	sIPTW - De novo metastatic	523	NR	NR	NR	Ribociclib better: 1.16 (0.96-1.39); NR	NR	NR	NR	NR
	Ribociclib + AI	sIPTW - De novo metastatic	645	NR	NR	NR		NR	NR	NR	NR

Study Name; Reference	Treatment	Subgroup	Sample size	Follow-up Time	Starting dose for CDK4/6i, n (%)	PFS			OS		
						Median (95% CI), months	HR (95% CI); P value	At latest timepoint, n (%)	Median (95% CI), months	HR (95% CI); P value	At latest timepoint, n (%)
	Abemaciclib + AI	sIPTW - No visceral disease	680	NR	NR	NR	Abemaciclib better: 0.98 (0.82-1.17); NR	NR	NR	NR	NR
	Ribociclib + AI	sIPTW - No visceral disease	831	NR	NR	NR		NR	NR	NR	NR
	Abemaciclib + AI	sIPTW - Visceral disease	358	NR	NR	NR	Abemaciclib similar: 1.00 (0.82-1.23); NR	NR	NR	NR	NR
	Ribociclib + AI	sIPTW - Visceral disease	443	NR	NR	NR		NR	NR	NR	NR
	Abemaciclib + AI	sIPTW - No bone-only disease	557	NR	NR	NR	Ribociclib better: 1.03 (0.86-1.23); NR	NR	NR	NR	NR
	Ribociclib + AI	sIPTW - No bone-only disease	683	NR	NR	NR		NR	NR	NR	NR
	Abemaciclib + AI	sIPTW - Bone-only disease	481	NR	NR	NR	Abemaciclib better: 0.92 (0.75-1.13); NR	NR	NR	NR	NR
	Ribociclib + AI	sIPTW - Bone-only disease	591	NR	NR	NR		NR	NR	NR	NR
	Abemaciclib + AI	sIPTW - Metastatic sites, 1	611	NR	NR	NR	Abemaciclib better: 0.98 (0.81-1.17); NR	NR	NR	NR	NR
	Ribociclib + AI	sIPTW - Metastatic sites, 1	745	NR	NR	NR		NR	NR	NR	NR
	Abemaciclib + AI	sIPTW - Metastatic sites, 2	240	NR	NR	NR	Abemaciclib better:	NR	NR	NR	NR

Study Name; Reference	Treatment	Subgroup	Sample size	Follow-up Time	Starting dose for CDK4/6i, n (%)	PFS			OS		
						Median (95% CI), months	HR (95% CI); P value	At latest timepoint, n (%)	Median (95% CI), months	HR (95% CI); P value	At latest timepoint, n (%)
	Ribociclib + AI	sIPTW - Metastatic sites, 2	304	NR	NR	NR	0.93 (0.73-1.19); NR	NR	NR	NR	NR
	Abemaciclib + AI	sIPTW - Metastatic sites, ≥ 3	92	NR	NR	NR	Ribociclib better: 1.08 (0.74-1.59); NR	NR	NR	NR	NR
	Ribociclib + AI	sIPTW - Metastatic sites, ≥ 3	111	NR	NR	NR		NR	NR	NR	NR
	Abemaciclib + AI	sIPTW - Metastatic sites, ND	95	NR	NR	NR	Ribociclib better: 1.18 (0.67-2.09); NR	NR	NR	NR	NR
	Ribociclib + AI	sIPTW - Metastatic sites, ND	114	NR	NR	NR		NR	NR	NR	NR
P-VERIFY 563-Rugo-2025; United States	Abemaciclib + AI	sIPTW - Age 18-49 years	118	NR	NR	NR	NR	NR	NR	Abemaciclib better: 0.86 (0.55-1.36); NR	NR
	Ribociclib + AI	sIPTW - Age 18-49 years	148	NR	NR	NR	NR	NR	NR		NR
	Abemaciclib + AI	sIPTW - Age 50-64 years	359	NR	NR	NR	NR	NR	NR	Abemaciclib better: 0.98 (0.73-1.31); NR	NR
	Ribociclib + AI	sIPTW - Age 50-64 years	437	NR	NR	NR	NR	NR	NR		NR
	Abemaciclib + AI	sIPTW - Age 65-74 years	316	NR	NR	NR	NR	NR	NR	Abemaciclib better: 0.91 (0.67-1.24); NR	NR
	Ribociclib + AI	sIPTW - Age 65-74 years	390	NR	NR	NR	NR	NR	NR		NR

Study Name; Reference	Treatment	Subgroup	Sample size	Follow-up Time	Starting dose for CDK4/6i, n (%)	PFS			OS		
						Median (95% CI), months	HR (95% CI); P value	At latest timepoint, n (%)	Median (95% CI), months	HR (95% CI); P value	At latest timepoint, n (%)
	Abemaciclib + AI	sIPTW - Age ≥ 75 years	246	NR	NR	NR	NR	NR	NR	Ribociclib better: 1.06 (0.77-1.47); NR	NR
	Ribociclib + AI	sIPTW - Age ≥ 75 years	299	NR	NR	NR	NR	NR	NR		NR
	Abemaciclib + AI	sIPTW - Race, White	654	NR	NR	NR	NR	NR	NR	Abemaciclib better: 0.83 (0.67-1.03); NR	NR
	Ribociclib + AI	sIPTW - Race, White	797	NR	NR	NR	NR	NR	NR		NR
	Abemaciclib + AI	sIPTW - Race, Black	94	NR	NR	NR	NR	NR	NR	Ribociclib better: 1.43 (0.87-2.35); NR	NR
	Ribociclib + AI	sIPTW - Race, Black	117	NR	NR	NR	NR	NR	NR		NR
	Abemaciclib + AI	sIPTW - Race, other	290	NR	NR	NR	NR	NR	NR	Ribociclib better: 1.19 (0.88-1.59); NR	NR
	Ribociclib + AI	sIPTW - Race, other	360	NR	NR	NR	NR	NR	NR		NR
	Abemaciclib + AI	sIPTW - ECOG PS at baseline, 0	369	NR	NR	NR	NR	NR	NR	Abemaciclib better: 0.63 (0.47-0.85); NR	NR
	Ribociclib + AI	sIPTW - ECOG PS at baseline, 0	457	NR	NR	NR	NR	NR	NR		NR

Study Name; Reference	Treatment	Subgroup	Sample size	Follow-up Time	Starting dose for CDK4/6i, n (%)	PFS			OS		
						Median (95% CI), months	HR (95% CI); P value	At latest timepoint, n (%)	Median (95% CI), months	HR (95% CI); P value	At latest timepoint, n (%)
	Abemaciclib + AI	sIPTW - ECOG PS at baseline, 1	275	NR	NR	NR	NR	NR	NR	Ribociclib better: 1.13 (0.83-1.53); NR	NR
	Ribociclib + AI	sIPTW - ECOG PS at baseline, 1	329	NR	NR	NR	NR	NR	NR		NR
	Abemaciclib + AI	sIPTW - ECOG PS at baseline, 2-4	119	NR	NR	NR	NR	NR	NR	Ribociclib better: 1.09 (0.69-1.72); NR	NR
	Ribociclib + AI	sIPTW - ECOG PS at baseline, 2-4	147	NR	NR	NR	NR	NR	NR		NR
	Abemaciclib + AI	sIPTW - ECOG PS at baseline, ND	275	NR	NR	NR	NR	NR	NR	Ribociclib better: 1.25 (0.88-1.77); NR	NR
	Ribociclib + AI	sIPTW - ECOG PS at baseline, ND	341	NR	NR	NR	NR	NR	NR		NR
	Abemaciclib + AI	sIPTW - De novo metastatic	523	NR	NR	NR	NR	NR	NR	Ribociclib better: 1.16 (0.93-1.45); NR	NR
	Ribociclib + AI	sIPTW - De novo metastatic	645	NR	NR	NR	NR	NR	NR		NR
	Abemaciclib + AI	sIPTW - No visceral disease	680	NR	NR	NR	NR	NR	NR	Abemaciclib better:	NR

Study Name; Reference	Treatment	Subgroup	Sample size	Follow-up Time	Starting dose for CDK4/6i, n (%)	PFS			OS		
						Median (95% CI), months	HR (95% CI); P value	At latest timepoint, n (%)	Median (95% CI), months	HR (95% CI); P value	At latest timepoint, n (%)
	Ribociclib + AI	sIPTW - No visceral disease	831	NR	NR	NR	NR	NR	NR	0.89 (0.72-1.11); NR	NR
	Abemaciclib + AI	sIPTW - Visceral disease	358	NR	NR	NR	NR	NR	NR	Ribociclib better: 1.09 (0.84-1.42); NR	NR
	Ribociclib + AI	sIPTW - Visceral disease	443	NR	NR	NR	NR	NR	NR		NR
	Abemaciclib + AI	sIPTW - No bone-only disease	557	NR	NR	NR	NR	NR	NR	Abemaciclib better: 0.98 (0.78-1.22); NR	NR
	Ribociclib + AI	sIPTW - No bone-only disease	683	NR	NR	NR	NR	NR	NR		NR
	Abemaciclib + AI	sIPTW - Bone-only disease	481	NR	NR	NR	NR	NR	NR	Abemaciclib better: 0.94 (0.73-1.22); NR	NR
	Ribociclib + AI	sIPTW - Bone-only disease	591	NR	NR	NR	NR	NR	NR		NR
	Abemaciclib + AI	sIPTW - Metastatic sites, 1	611	NR	NR	NR	NR	NR	NR	Abemaciclib better: 0.93 (0.74-1.16); NR	NR
	Ribociclib + AI	sIPTW - Metastatic sites, 1	745	NR	NR	NR	NR	NR	NR		NR
	Abemaciclib + AI	sIPTW - Metastatic sites, 2	240	NR	NR	NR	NR	NR	NR	Abemaciclib better: 0.97 (0.72-1.32); NR	NR
	Ribociclib + AI	sIPTW - Metastatic sites, 2	304	NR	NR	NR	NR	NR	NR		NR

Study Name; Reference	Treatment	Subgroup	Sample size	Follow-up Time	Starting dose for CDK4/6i, n (%)	PFS			OS		
						Median (95% CI), months	HR (95% CI); P value	At latest timepoint, n (%)	Median (95% CI), months	HR (95% CI); P value	At latest timepoint, n (%)
	Abemaciclib + AI	sIPTW - Metastatic sites, ≥ 3	92	NR	NR	NR	NR	NR	NR	Ribociclib better: 1.10 (0.68-1.78); NR	NR
	Ribociclib + AI	sIPTW - Metastatic sites, ≥ 3	111	NR	NR	NR	NR	NR	NR		NR
	Abemaciclib + AI	sIPTW - Metastatic sites, ND	95	NR	NR	NR	NR	NR	NR	Abemaciclib better: 0.94 (0.47-1.85); NR	NR
	Ribociclib + AI	sIPTW - Metastatic sites, ND	114	NR	NR	NR	NR	NR	NR		NR
P-VERIFY SABS24-102-Rugo-2024[a]; United States	Ribociclib + AI	sIPTW - Age 18-49 years	148	NR	NR	NR	NR	NR	NR	Abemaciclib better: 0.86 (0.55-1.36); NR	NR
	Abemaciclib + AI	sIPTW - Age 18-49 years	118	NR	NR	NR	NR	NR	NR		NR
	Ribociclib + AI	sIPTW - Age 50-64 years	437	NR	NR	NR	NR	NR	NR	Abemaciclib better: 0.98 (0.73-1.31); NR	NR
	Abemaciclib + AI	sIPTW - Age 50-64 years	359	NR	NR	NR	NR	NR	NR		NR
	Ribociclib + AI	sIPTW - Age 65-75 years	390	NR	NR	NR	NR	NR	NR	Abemaciclib better: 0.91 (0.67-1.24); NR	NR
	Abemaciclib + AI	sIPTW - Age 65-75 years	316	NR	NR	NR	NR	NR	NR		NR
	Ribociclib + AI	sIPTW - Age ≥ 75 years	299	NR	NR	NR	NR	NR	NR	Ribociclib better:	NR

Study Name; Reference	Treatment	Subgroup	Sample size	Follow-up Time	Starting dose for CDK4/6i, n (%)	PFS			OS		
						Median (95% CI), months	HR (95% CI); P value	At latest timepoint, n (%)	Median (95% CI), months	HR (95% CI); P value	At latest timepoint, n (%)
	Abemaciclib + AI	sIPTW - Age ≥ 75 years	246	NR	NR	NR	NR	NR	NR	1.06 (0.77-1.47); NR	NR
	Ribociclib + AI	sIPTW - Race, White	797	NR	NR	NR	NR	NR	NR	Abemaciclib better: 0.83 (0.67-1.03); NR	NR
	Abemaciclib + AI	sIPTW - Race, White	654	NR	NR	NR	NR	NR	NR		NR
	Ribociclib + AI	sIPTW - Race, Black	115	NR	NR	NR	NR	NR	NR	Ribociclib better: 1.43 (0.87-2.35); NR	NR
	Abemaciclib + AI	sIPTW - Race, Black	94	NR	NR	NR	NR	NR	NR		NR
	Ribociclib + AI	sIPTW - Race, other	360	NR	NR	NR	NR	NR	NR	Ribociclib better: 1.19 (0.88-1.59); NR	NR
	Abemaciclib + AI	sIPTW - Race, other	290	NR	NR	NR	NR	NR	NR		NR
	Ribociclib + AI	sIPTW - ECOG PS at baseline, 0	457	NR	NR	NR	NR	NR	NR	Abemaciclib better: 0.63 (0.47-0.85); NR	NR
	Abemaciclib + AI	sIPTW - ECOG PS at baseline, 0	369	NR	NR	NR	NR	NR	NR		NR
	Ribociclib + AI	sIPTW - ECOG PS at baseline, 1	329	NR	NR	NR	NR	NR	NR	Ribociclib better: 1.13 (0.83-1.53); NR	NR

Study Name; Reference	Treatment	Subgroup	Sample size	Follow-up Time	Starting dose for CDK4/6i , n (%)	PFS			OS		
						Median (95% CI), months	HR (95% CI); P value	At latest timepoint, n (%)	Median (95% CI), months	HR (95% CI); P value	At latest timepoint, n (%)
	Abemaciclib + AI	sIPTW - ECOG PS at baseline, 1	275	NR	NR	NR	NR	NR	NR		NR
	Ribociclib + AI	sIPTW - ECOG PS at baseline, 2 -4	147	NR	NR	NR	NR	NR	NR	Ribociclib better: 1.09 (0.69-1.72); NR	NR
	Abemaciclib + AI	sIPTW - ECOG PS at baseline, 2 -4	119	NR	NR	NR	NR	NR	NR		NR
	Ribociclib + AI	sIPTW - ECOG PS at baseline, ND	341	NR	NR	NR	NR	NR	NR	Ribociclib better: 1.25 (0.88-1.77); NR	NR
	Abemaciclib + AI	sIPTW - ECOG PS at baseline, ND	275	NR	NR	NR	NR	NR	NR		NR
	Ribociclib + AI	sIPTW - De novo metastatic	645	NR	NR	NR	NR	NR	NR	Ribociclib better: 1.16 (0.93-1.45); NR	NR
	Abemaciclib + AI	sIPTW - De novo metastatic	523	NR	NR	NR	NR	NR	NR		NR
	Ribociclib + AI	sIPTW - No visceral disease	831	NR	NR	NR	NR	NR	NR	Abemaciclib better: 0.89 (0.72-1.11); NR	NR
	Abemaciclib + AI	sIPTW - No visceral disease	680	NR	NR	NR	NR	NR	NR		NR

Study Name; Reference	Treatment	Subgroup	Sample size	Follow-up Time	Starting dose for CDK4/6i, n (%)	PFS			OS		
						Median (95% CI), months	HR (95% CI); P value	At latest timepoint, n (%)	Median (95% CI), months	HR (95% CI); P value	At latest timepoint, n (%)
	Ribociclib + AI	sIPTW - Visceral disease	443	NR	NR	NR	NR	NR	NR	Ribociclib better: 1.09 (0.84-1.42); NR	NR
	Abemaciclib + AI	sIPTW - Visceral disease	358	NR	NR	NR	NR	NR	NR		NR
	Ribociclib + AI	sIPTW - No bone-only disease	683	NR	NR	NR	NR	NR	NR	Abemaciclib better: 0.98 (0.78-1.22); NR	NR
	Abemaciclib + AI	sIPTW - No bone-only disease	557	NR	NR	NR	NR	NR	NR		NR
	Ribociclib + AI	sIPTW - Bone only disease	591	NR	NR	NR	NR	NR	NR	Abemaciclib better: 0.94 (0.73-1.22); NR	NR
	Abemaciclib + AI	sIPTW - Bone only disease	481	NR	NR	NR	NR	NR	NR		NR
	Ribociclib + AI	sIPTW - Metastatic sites, 1	745	NR	NR	NR	NR	NR	NR	Abemaciclib better: 0.93 (0.74-1.16); NR	NR
	Abemaciclib + AI	sIPTW - Metastatic sites, 1	611	NR	NR	NR	NR	NR	NR		NR
	Ribociclib + AI	sIPTW - metastatic sites, 2	304	NR	NR	NR	NR	NR	NR	Abemaciclib better: 0.97 (0.72-1.32); NR	NR
	Abemaciclib + AI	sIPTW - metastatic sites, 2	240	NR	NR	NR	NR	NR	NR		NR
	Ribociclib + AI	sIPTW - metastatic sites, ≥3	111	NR	NR	NR	NR	NR	NR	Ribociclib better:	NR

Study Name; Reference	Treatment	Subgroup	Sample size	Follow-up Time	Starting dose for CDK4/6i, n (%)	PFS			OS		
						Median (95% CI), months	HR (95% CI); P value	At latest timepoint, n (%)	Median (95% CI), months	HR (95% CI); P value	At latest timepoint, n (%)
	Abemaciclib + AI	sIPTW - metastatic sites, ≥3	92	NR	NR	NR	NR	NR	NR	1.10 (0.68-1.78); NR	NR
	Ribociclib + AI	sIPTW - metastatic sites, NS	114	NR	NR	NR	NR	NR	NR	Abemaciclib better: 0.94 (0.47-1.85); NR	NR
	Abemaciclib + AI	sIPTW - metastatic sites, NS	95	NR	NR	NR	NR	NR	NR		NR

Note: For records with a high volume of subgroup data, subgroups outlined in Appendix F were prioritized.
Note: The “Study Name; Reference” cells shaded in grey highlight full text records, while non-shaded are abstracts/manuscripts.
Note: Overall follow-up time was used where subgroup-specific follow-up times were not reported.
[a] Different data reported in the poster and the abstract.
Abbreviations: AI = aromatase inhibitor; CDK4/6i = cyclin-dependent kinase 4/6 inhibitors; CI = confidence interval; ECOG PS = eastern cooperative oncology group performance status; ER = estrogen receptor; ET = endocrine therapy; HR = hazard ratio; ILC = invasive lobular carcinoma; IQR = interquartile range; IPTW = inverse probability of treatment weighting; ND = not documented; NR = not reported; NS = not specified; OS = overall survival; PFS = progression-free survival; PR = progesterone receptor; RWE = real-world evidence; sIPTW = stabilized inverse probability treatment weighting

Progression-free survival and overall survival for first-line in comparative RWE studies (studies with only abstract data available)

Table S10. Progression-free survival and overall survival for first-line palbociclib in comparative RWE studies versus ribociclib (overall population; studies with only abstract data available)

Study Name; Reference	Treatment	Subgroup	Sample size	Follow-up Time	Starting dose for CDK4/6i, n (%)	PFS			OS		
						Median (95% CI), months	HR (95% CI); P value	At latest timepoint, n (%)	Median (95% CI), months	HR (95% CI); P value	At latest timepoint, n (%)
5942-Plavetic-2024; Croatia	Palbociclib +ET	All patients	76	NR	NR	47 (30-49)	NR	NR	52 (42-54)	NR	NR
	Ribociclib + ET	All patients	72	NR	NR	48 (27-53)	NR	NR	62 (40-62)	NR	NR
5957-Orlova-2024; Russia	Palbociclib + ET	All patients	NR	NR	NR	Not reached	NR; P=0.602	NR	NR	NR	NR
	Ribociclib + ET	All patients	NR	NR	NR	Not reached		NR	NR	NR	NR
6118-Duchnowska-2024[a]; Poland	Palbociclib + AI	All patients	NR	Median 29.6 months	NR	29.0 (21.6-37.0)	Ribociclib better: 0.97 (0.76-1.25); P=0.8	NR	48.9 (42.9-not reached)	Ribociclib similar: 0.99 (0.74-1.33); P= 0.9	NR
	Ribociclib + AI	All patients	NR		NR	32.0 (24.5-36.9)		NR	48.6 (41.9-not reached)		NR
	Palbociclib + FUL	All patients	NR		NR	NR	Ribociclib better: 0.57 (0.41-0.79); P<0.001	NR	NR	Ribociclib better: 0.65 (0.44-0.95); P=0.026	NR
	Ribociclib + FUL	All patients	NR		NR	NR		NR	NR		NR
ESMO23-022-Georgina Gullick-	Palbociclib + ET	All patients	473	Median 28 (range	NR	31 (25-35)	NR	NR	NR	NR	NR

Study Name; Reference	Treatment	Subgroup	Sample size	Follow-up Time	Starting dose for CDK4/6i , n (%)	PFS			OS		
						Median (95% CI), months	HR (95% CI); P value	At latest timepoint , n (%)	Median (95% CI), months	HR (95% CI); P value	At latest timepoin t, n (%)
2023; United Kingdom	Ribociclib + ET	All patients	38	0-76) months	NR	44 (21- not reached)	NR	NR	NR	NR	NR
ESMO23-066-Lenza-2023; Spain	Palbociclib + ET	All patients	282[b]	NR	NR	16 (NR)	NR	NR	44 (NR)	NR	NR
	Ribociclib + ET	All patients	216[b]	NR	NR	14 (NR)	NR	NR	52 (NR)	NR	NR
ESMOAsia24-002-Tsareva-2024; Russia	Palbociclib + ET	All patients	218	Median 24 months	NR	30 (NR)	Palbociclib better: 1.1 (0.8-1.5); NR	2 year: NR (56)	54 (NR)	Palbociclib better: 1.28 (0.87-1.89); NR	2 year: NR (76)
	Ribociclib + ET	All patients	159		NR	26 (NR)		2 year: NR (51)	39 (NR)		2 year: NR (78)
GOIRC-04-2019; SABCS23-009-L. Moscetti-2023; Europe	Palbociclib + AI/FUL	All patients	61	NR	NR	23.43 (15.4-31.5)	NR	NR	NR	NR	NR
	Ribociclib + AI/FUL	All patients	44	NR	NR	39.9 (30.9-49.0)	NR	NR	NR	NR	NR
SABCS23-065-Weipert-2023; Spain	Palbociclib + ET	All patients	608	Median 30.1 months	NR	NR	NR	NR	58 (58 – not reached)	Palbociclib better: 1.04 (0.61-1.77); P=0.899	NR
	Ribociclib + ET	All patients	91	Median 28.5 months	NR	NR	NR	NR	Not reached		NR
SABCS24-040-Brufsky-2024[a]; United States	Palbociclib + ET	Flatiron Health database	3504	Median 27.4 (range: 0.2-96.7) months	NR	NR	NR	NR	43.8 (41.7–46.6)	Ribociclib better: 1.07 (0.90-1.28); P=0.422	NR

Study Name; Reference	Treatment	Subgroup	Sample size	Follow-up Time	Starting dose for CDK4/6i, n (%)	PFS			OS		
						Median (95% CI), months	HR (95% CI); P value	At latest timepoint, n (%)	Median (95% CI), months	HR (95% CI); P value	At latest timepoint, n (%)
	Ribociclib + ET	Flatiron Health database	488	Median 14.2 (range: 0.2-74.9) months	NR	NR	NR	NR	44.2 (37.3-51.1)		NR
	Palbociclib + AI	Flatiron Health database	2334	NR	NR	NR	NR	NR	47.4 (45.0-49.9)	Ribociclib better: 1.08 (0.88-1.33); P=0.448	NR
	Ribociclib + AI	Flatiron Health database	355	NR	NR	NR	NR	NR	49.8 (42.0-53.8)		NR
	Palbociclib + ET	Tempus database	475	Median 24.4 (range: 11.6-37.5) months	NR	NR	NR	NR	NR	Ribociclib better: 0.85 (0.44-1.63); P=0.62	NR
	Ribociclib + ET	Tempus database	32	Median 25.6 (range: 17.4-33.7) months	NR	NR	NR	NR	NR		NR
	Palbociclib + AI	Tempus database	329	NR	NR	NR	NR	NR	NR	Ribociclib better: 0.72 (0.36-1.45); P=0.36	NR
	Ribociclib + AI	Tempus database	23	NR	NR	NR	NR	NR	NR		NR
SABCS24-056-Betiol-2024; Global	Ribociclib + ET	Analysis 2: Ribociclib vs Palbociclib	980	NR	NR	NR	NR	NR	1946 days	Ribociclib better: 1.441 (1.200 -1.731); NR	NR

Study Name; Reference	Treatment	Subgroup	Sample size	Follow-up Time	Starting dose for CDK4/6i , n (%)	PFS			OS		
						Median (95% CI), months	HR (95% CI); P value	At latest timepoint , n (%)	Median (95% CI), months	HR (95% CI); P value	At latest timepoin t, n (%)
	Palbociclib + ET	Analysis 2: Ribociclib vs Palbociclib	980	NR	NR	NR	NR	NR	1286 days		NR
3400-Tang-2023; United Kingdom	Palbociclib + AI	Whole cohort	114	Median 49.8 months	NR	23.9 (NR)	Palbociclib better: 0.88 (0.56-1.40); P=0.60	NR	49.5 (NR)	Palbociclib better: 0.94 (0.55-1.62); P=0.94	NR
	Ribociclib + AI	Whole cohort	38		NR	19.8 (NR)		NR	40.4 (NR)		NR
1118-Trigueros-2025; Costa Rica	Ribociclib + ET	All patients	NR	NR	NR	29.8 (23.1-37.5)	NR	NR	46.7 (31.5-59.3)	NR	NR
	Palbociclib + ET	All patients	NR	NR	NR	28.4 (21.2-34.6)	NR	NR	40.2 (32.1-52.8)	NR	NR

[a] The poster and abstract report different data for this study.
[b] Sample size values calculated from percentages provided in study.
Abbreviations: AI = aromatase inhibitor; CDK4/6i = cyclin-dependent kinase 4/6 inhibitors; CI = confidence interval; ET = endocrine therapy; FUL = fulvestrant; HR = hazard ratio; NR = not reported; OS = overall survival; PFS = progression-free survival; RWE = real-world evidence.

Table S11. Progression-free survival and overall survival for first-line palbociclib in comparative RWE studies versus ribociclib (subgroups; studies with only abstract data available)

Study Name; Reference	Treatment	Subgroup	Sample size	Follow-up Time	Starting dose for CDK4/6i, n (%)	PFS			OS		
						Median (95% CI), months	HR (95% CI); P value	At latest timepoint, n (%)	Median (95% CI), months	HR (95% CI); P value	At latest timepoint, n (%)
ESMO23-066-Lenza-2023; Spain	Palbociclib + ET	<2 year hormone therapy	NR	NR	NR	8 (NR)	NR	NR	22 (NR)	NR	NR
	Ribociclib + ET	<2 year hormone therapy	NR	NR	NR	6 (NR)	NR	NR	28 (NR)	NR	NR
	Palbociclib + ET	Visceral	NR	NR	NR	NR	NR	NR	29 (NR)	NR	NR
	Ribociclib + ET	Visceral	NR	NR	NR	NR	NR	NR	53 (NR)	NR	NR
ESMOAsia24-002-Tsareva-2024; Russia	Palbociclib + ET	Liver metastases	NR	NR	NR	16 (NR)	Palbociclib better: 0.88 (0.4-1.9); NR	NR	NR	NR	NR
	Ribociclib + ET	Liver metastases	NR	NR	NR	20 (NR)		NR	NR	NR	NR
	Palbociclib + ET	Primary endocrine resistance	NR	NR	NR	9 (NR)	Ribociclib better: 1.83 (0.7-4.5); NR	NR	NR	NR	NR
	Ribociclib + ET	Primary endocrine resistance	NR	NR	NR	15 (NR)		NR	NR	NR	NR
3400-Tang-2023; United Kingdom	Palbociclib + AI	Recurrent disease cohort	NR	NR	NR	NR	NR	NR	48.4 (NR)	Ribociclib better:	NR

Study Name; Reference	Treatment	Subgroup	Sample size	Follow-up Time	Starting dose for CDK4/6i, n (%)	PFS			OS		
						Median (95% CI), months	HR (95% CI); P value	At latest timepoint, n (%)	Median (95% CI), months	HR (95% CI); P value	At latest timepoint, n (%)
	Ribociclib + AI	Recurrent disease cohort	NR	NR	NR	NR	NR	NR	40.4 (NR)	1.12 (0.63-1.98); P=0.69	NR
	Palbociclib + AI	De novo disease	NR	NR	NR	NR	NR	NR	77.4 (NR)	Palbociclib better: 0.83 (0.17-4.05); P=0.80	NR
	Ribociclib + AI	De novo disease	NR	NR	NR	NR	NR	NR	Not reached		NR

Note: For records with a high volume of subgroup data, subgroups outlined in Appendix F were prioritized.
Abbreviations: AI = aromatase inhibitor; CDK4/6i = cyclin-dependent kinase 4/6 inhibitors; CI = confidence interval; ET = endocrine therapy; HR = hazard ratio; NR = not reported; OS = overall survival; PFS = progression-free survival; RWE = real-world evidence.

Table S12. Progression-free survival and overall survival for first-line palbociclib in comparative RWE studies versus abemaciclib (overall population; studies with only abstract data available)

Study Name; Reference	Treatment	Subgroup	Sample size	Follow-up Time	Starting dose for CDK4/6i, n (%)	PFS			OS		
						Median (95% CI), months	HR (95% CI); P value	At latest timepoint, n (%)	Median (95% CI), months	HR (95% CI); P value	At latest timepoint, n (%)
5942-Plavetic-2024; Croatia	Palbociclib +ET	All patients	76	NR	NR	47 (30-49)	NR	NR	52 (42-54)	NR	NR
	Abemaciclib + ET	All patients	24	NR	NR	Not reached	NR	NR	Not reached	NR	NR
6118-Duchnowska-2024[a]; Poland	Palbociclib + AI	All patients	NR	Median 29.6 months	NR	29.0 (21.6-37.0)	Abemaciclib better: 0.80 (0.55-1.18); P=0.3	NR	48.9 (42.9-not reached)	Abemaciclib better: 0.90 (0.56-1.44); P=0.7	NR
	Abemaciclib + AI	All patients	NR		NR	Not reached (21.9- not reached)		NR	Not reached (36.7-not reached)		NR
	Palbociclib + FUL	All patients	NR		NR	NR	Abemaciclib better: 0.93 (0.65-1.34); P=0.7	NR	NR	Abemaciclib better: 0.96 (0.63-1.46); P=0.9	NR
	Abemaciclib + FUL	All patients	NR		NR	NR		NR	NR		NR
ESMO23-022-Georgina Gullick-	Palbociclib + ET	All patients	473	Median 28 (range 0-	NR	31 (25-35)	NR	NR	NR	NR	NR

Study Name; Reference	Treatment	Subgroup	Sample size	Follow-up Time	Starting dose for CDK4/6i, n (%)	PFS			OS		
						Median (95% CI), months	HR (95% CI); P value	At latest timepoint, n (%)	Median (95% CI), months	HR (95% CI); P value	At latest timepoint, n (%)
2023; United Kingdom	Abemaciclib + ET	All patients	33	76) months	NR	16 (9-not reached)	NR	NR	NR	NR	NR
ESMO23-066-Lenza-2023; Spain	Palbociclib + ET	All patients	282[b]	NR	NR	16 (NR)	NR	NR	44 (NR)	NR	NR
	Abemaciclib + ET	All patients	86[b]	NR	NR	17 (NR)	NR	NR	Not reached	NR	NR
GOIRC-04-2019; SABCS23-009-L. Moscetti-2023; ; Europe	Palbociclib + AI/FUL	All patients	61	NR	NR	23.43 (15.4-31.5)	NR	NR	NR	NR	NR
	Abemaciclib + AI/FUL	All patients	25	NR	NR	23.2 (13.3-33.1)	NR	NR	NR	NR	NR
SABCS23-065-Weipert-2023; Spain	Palbociclib + ET	All patients	608	Median 30.1 months	NR	NR	NR	NR	58 (58 – not reached)	Palbociclib better: 1.29 (0.85-1.96); P=0.229	NR
	Abemaciclib + ET	All patients	133	Median 24.1 months	NR	NR	NR	NR	Not reached (56 – not reached)		NR
SABCS24-040-Brufsky-2024[a]; United States	Palbociclib + ET	Flatiron Health database	3504	Median 27.4 (range: 0.2-96.7) months	NR	NR	NR	NR	43.8 (41.7–46.6)	Abemaciclib better: 1.09 (0.89-1.33); P=0.402	NR
	Abemaciclib + ET	Flatiron Health database	575	Median 19.4 (range: 0.2-70.9) months	NR	NR	NR	NR	43.2 (34.5–48.8)		NR

Study Name; Reference	Treatment	Subgroup	Sample size	Follow-up Time	Starting dose for CDK4/6i, n (%)	PFS			OS		
						Median (95% CI), months	HR (95% CI); P value	At latest timepoint, n (%)	Median (95% CI), months	HR (95% CI); P value	At latest timepoint, n (%)
	Palbociclib + AI	Flatiron Health database	2334	NR	NR	NR	NR	NR	47.4 (45.0-49.9)	Palbociclib better: 0.9 (0.66-1.23); P=0.506	NR
	Abemaciclib + AI	Flatiron Health database	341	NR	NR	NR	NR	NR	48.3 (37.8-NA)		NR
	Palbociclib + ET	Tempus database	475	Median 24.4 (range: 11.6-37.5) months	NR	NR	NR	NR	NR	Abemaciclib better: 0.95 (0.41-2.21); P=0.91	NR
	Abemaciclib + ET	Tempus database	32	Median 20.7 (range: 11.0-30.0) months	NR	NR	NR	NR	NR		NR
	Palbociclib + AI	Tempus database	329	NR	NR	NR	NR	NR	NR	Palbociclib better: 1.21 (0.40-3.64); P=0.73	NR
	Abemaciclib + AI	Tempus database	14	NR	NR	NR	NR	NR	NR		NR
SABCS24-056-Betiol-2024; Global	Palbociclib + ET	Analysis 3: Palbociclib vs Abemaciclib	318	NR	NR	NR	NR	NR	1124 days	Abemaciclib better: 1.56 (1.165-2.091); NR	NR
	Abemaciclib + ET	Analysis 3: Palbociclib vs Abemaciclib	318	NR	NR	NR	NR	NR	1706 days		NR

[a] The poster and abstract report different data for this study.
[b] Sample size values calculated from percentages provided in study.
Abbreviations: AI = aromatase inhibitor; CDK4/6i = cyclin-dependent kinase 4/6 inhibitors; CI = confidence interval; ET = endocrine therapy; FUL = fulvestrant; HR = hazard ratio; NR = not reported; OS = overall survival; PFS = progression-free survival; RWE = real-world evidence.

Table S13. Progression-free survival and overall survival for first-line palbociclib in comparative RWE studies versus abemaciclib (subgroups; studies with only abstract data available)

Study Name; Reference	Treatment	Subgroup	Sample size	Follow-up Time	Starting dose for CDK4/6i, n (%)	PFS			OS		
						Median (95% CI), months	HR (95% CI); P value	At latest timepoint, n (%)	Median (95% CI), months	HR (95% CI); P value	At latest timepoint, n (%)
ESMO23-066-Lenza-2023; Spain	Palbociclib + ET	<2 year hormone therapy	NR	NR	NR	8 (NR)	NR	NR	22 (NR)	NR	NR
	Abemaciclib + ET	<2 year hormone therapy	NR	NR	NR	12 (NR)	NR	NR	Not reached	NR	NR
	Palbociclib + ET	Visceral	NR	NR	NR	NR	NR	NR	29 (NR)	NR	NR
	Abemaciclib + ET	Visceral	NR	NR	NR	NR	NR	NR	Not reached	NR	NR

Note: For records with a high volume of subgroup data, subgroups outlined in Appendix F were prioritized.
Abbreviations: CDK4/6i = cyclin-dependent kinase 4/6 inhibitors; CI = confidence interval; ET = endocrine therapy; HR = hazard ratio; NR = not reported; OS = overall survival; PFS = progression-free survival; RWE = real-world evidence.

Table S14. Progression-free survival and overall survival for first-line ribociclib in comparative RWE studies versus abemaciclib (overall population; studies with only abstract data available)

Study Name; Reference	Treatment	Subgroup	Sample size	Follow-up Time	Starting dose for CDK4/6i, n (%)	PFS			OS		
						Median (95% CI), months	HR (95% CI); P value	At latest timepoint, n (%)	Median (95% CI), months	HR (95% CI); P value	At latest timepoint, n (%)
5942-Plavetic-2024; Croatia	Abemaciclib + ET	All patients	24	NR	NR	Not reached	NR	NR	Not reached	NR	NR
	Ribociclib + ET	All patients	72	NR	NR	48 (27-53)	NR	NR	62 (40-62)	NR	NR
6118-Duchnowska-2024[a]; Poland	Ribociclib + AI	All patients	NR	Median 29.6 months	NR	32.0 (24.5-36.9)	Abemaciclib better: 1.21 (0.84-1.74); P=0.3	NR	48.6 (41.9-not reached)	Abemaciclib better: 1.10 (0.70-1.73); P=0.7	NR
	Abemaciclib + AI	All patients	NR		NR	Not reached (21.9-not reached)		NR	Not reached (36.7-not reached)		NR
	Ribociclib + FUL	All patients	NR		NR	NR	Ribociclib better: 0.61 (0.44-0.90); P=0.011	NR	NR	Ribociclib better: 0.67 (0.43-1.05); P= 0.084	NR
	Abemaciclib + FUL	All patients	NR		NR	NR		NR	NR		NR
ESMO23-022-Georgina Gullick-2023; United Kingdom	Abemaciclib + ET	All patients	33	Median 28 (range 0-76) months	NR	16 (9-not reached)	NR	NR	NR	NR	NR
	Ribociclib + ET	All patients	38		NR	44 (21-not reached)	NR	NR	NR	NR	NR
ESMO23-066-Lenza-2023; Spain	Ribociclib + ET	All patients	216[b]	NR	NR	14 (NR)	NR	NR	52 (NR)	NR	NR

Study Name; Reference	Treatment	Subgroup	Sample size	Follow-up Time	Starting dose for CDK4/6i , n (%)	PFS			OS		
						Median (95% CI), months	HR (95% CI); P value	At latest timepoint, n (%)	Median (95% CI), months	HR (95% CI); P value	At latest timepoint, n (%)
	Abemaciclib + ET	All patients	86[b]	NR	NR	17 (NR)	NR	NR	Not reached	NR	NR
GOIRC-04-2019; SABCS23-009-L. Moscetti-2023; ; Europe	Ribociclib + AI/FUL	All patients	44	NR	NR	39.9 (30.9-49.0)	NR	NR	NR	NR	NR
	Abemaciclib + AI/FUL	All patients	25	NR	NR	23.2 (13.3-33.1)	NR	NR	NR	NR	NR
SABCS23-065-Weipert-2023; Spain	Abemaciclib + ET	All patients	133	Median 24.1 months	NR	NR	NR	NR	Not reached (56 – not reached)	NR	NR
	Ribociclib + ET	All patients	91	Median 28.5 months	NR	NR	NR	NR	Not reached	NR	NR
SABCS24-040-Brufsky-2024[a]; United States	Abemaciclib + ET	Flatiron Health database	575	Median 19.4 (range: 0.2-70.9) months	NR	NR	NR	NR	43.2 (34.5–48.8)	NR	NR
	Ribociclib + ET	Flatiron Health database	488	Median 14.2 (range: 0.2-74.9) months	NR	NR	NR	NR	44.2 (37.3-51.1)	NR	NR
	Abemaciclib + AI	Flatiron Health database	341	NR	NR	NR	NR	NR	48.3 (37.8-NA)	NR	NR
	Ribociclib + AI	Flatiron Health database	355	NR	NR	NR	NR	NR	49.8 (42.0-53.8)	NR	NR

Study Name; Reference	Treatment	Subgroup	Sample size	Follow-up Time	Starting dose for CDK4/6i, n (%)	PFS			OS		
						Median (95% CI), months	HR (95% CI); P value	At latest timepoint, n (%)	Median (95% CI), months	HR (95% CI); P value	At latest timepoint, n (%)
SABCS24-056-Betiol-2024; Global	Ribociclib + ET	Analysis 1: Ribociclib vs Abemaciclib	271	NR	NR	NR	NR	NR	NR	Ribociclib better: 0.964 (0.634-1.467); NR	5 years: NR (61.82)
	Abemaciclib + ET	Analysis 1: Ribociclib vs Abemaciclib	271	NR	NR	NR	NR	NR	NR		5 years: NR (53.66)

[a] The poster and abstract report different data for this study.
[b] Sample size values calculated from percentages provided in study.
Abbreviations: AI = aromatase inhibitor; CDK4/6i = cyclin-dependent kinase 4/6 inhibitors; CI = confidence interval; ET = endocrine therapy; FUL = fulvestrant; HR = hazard ratio; NA = not available; NR = not reported; OS = overall survival; PFS = progression-free survival; RWE = real-world evidence.

Table S15. Progression-free survival and overall survival for first-line ribociclib in comparative RWE studies versus abemaciclib (subgroups; studies with only abstract data available)

Study Name; Reference	Treatment	Subgroup	Sample size	Follow-up Time	Starting dose for CDK4/6i, n (%)	PFS Median (95% CI), months	PFS HR (95% CI); P value	PFS At latest timepoint, n (%)	OS Median (95% CI), months	OS HR (95% CI); P value	OS At latest timepoint, n (%)
ESMO23-066-Lenza-2023; Spain	Ribociclib + ET	<2 year hormone therapy	NR	NR	NR	6 (NR)	NR	NR	28 (NR)	NR	NR
	Abemaciclib + ET	<2 year hormone therapy	NR	NR	NR	12 (NR)	NR	NR	Not reached	NR	NR
	Ribociclib + ET	Visceral	NR	NR	NR	NR	NR	NR	53 (NR)	NR	NR
	Abemaciclib + ET	Visceral	NR	NR	NR	NR	NR	NR	Not reached	NR	NR

[a] Sample size values calculated from percentages provided in study.

Note: For records with a high volume of subgroup data, subgroups outlined in Appendix F were prioritized.

Abbreviations: CDK4/6i = cyclin-dependent kinase 4/6 inhibitors; CI = confidence interval; ET = endocrine therapy; HR = hazard ratio; NR = not reported; OS = overall survival; PFS = progression-free survival; RWE = real-world evidence.

Supplementary Information S2. Summary of subgroup OS and PFS results

PFS Subgroup Results

Ribociclib demonstrated superior PFS compared to palbociclib in select high-risk populations. In the PALMARES-2 study, ribociclib showed statistically significant benefit in premenopausal patients (HR 0.57, 95%CI: 0.46-0.70; P<0.001), luminal B-like tumors (HR 0.81, 95%CI: 0.75-0.88; P<0.001), patients with endocrine resistance (HR: 0.75, 95%CI: 0.58-0.98; P=0.034), patients with non-invasive lobular carcinoma disease (HR 0.78, 95%CI: 0.96-0.93; P=0.005), and patients with liver metastases (HR 0.89, 95%CI: 0.79-0.99; P=0.036). Patients with de novo metastatic disease treated with ribociclib were also found to have favorable PFS outcomes (HR 0.76, 95%CI: 0.61-0.94; P=0.010). In contrast, PALMARES-2 found palbociclib to provide a statistically significant benefit in older patients (HR 1.09, 95%CI: 1.02-1.17; P=0.008).

Abemaciclib demonstrated superior PFS compared to palbociclib, particularly in high-risk and endocrine-sensitive populations. In the PALMARES-2 study, abemaciclib showed statistically significant benefit in endocrine-sensitive patients (HR 0.75, 95%CI: 0.64-0.87; P<0.001), endocrine-resistant patients (HR 0.77, 95%CI: 0.63-0.93; P=0.008), those with luminal B-like tumors (HR 0.76, 95%CI: 0.65-0.90; P=0.002), and those with non-invasive lobular carcinoma disease (HR 0.70, 95%CI: 0.55-0.88; P=0.002). Additional significant findings favoring abemaciclib were observed in premenopausal patients (HR 0.59, 95%CI: 0.39-0.89; P=0.013), patients with poor ECOG performance status (HR 0.74, 95%CI: 0.55-0.99; P=0.048), and those with de novo metastatic disease (HR 0.52, 95%CI: 0.37-0.73; P<0.001). Real-world data from two additional studies also supported abemaciclib benefit in endocrine-sensitive patients (HR 2.41, 95%CI: 1.09-5.31; P=0.029) and patients with an ECOG performance status of 0 at baseline (HR 0.79, 95%CI: 0.67-0.93).

Across patient subgroup analyses, abemaciclib demonstrated a modest trend toward improved PFS compared to ribociclib, though significant findings were not common. In patients with non-visceral disease, abemaciclib showed significantly superior PFS (P=0.038), with median PFS not reached, versus 36.01 months for ribociclib. In the PALMARES-2 study, abemaciclib showed a favorable trend in patients with de novo metastatic disease (HR 0.69, 95%CI: 0.60-0.79; P<0.001).

Despite the small number of statistically significant HRs, PFS was generally comparable across all three agents in subgroup analyses. These analyses were limited by variable sample sizes (12 to 4,458 patients, across subgroups reporting HRs), with many analyses not reporting sample sizes, which introduces considerable heterogeneity and heavily limits the reliability of these results.

Additional details can be found in **Appendix G and Appendix H**.

OS Subgroup Results Summary

Across patient subgroups, differences in OS between ribociclib and palbociclib were generally not statistically significant. However, in the CEPRA study, ribociclib demonstrated a favorable trend in patients according to ECOG performance status (per 1 ECOG) (HR 3.01, 95%CI: 1.08-8.37; P=0.03).

For abemaciclib and palbociclib, differences in OS were inconsistent and often not statistically significant. In the P-VERIFY study, patients aged 18-49 years (HR 0.68, 95%CI: 0.46-0.99) and those with ECOG 0 at baseline (HR 0.61, 95%CI: 0.48-0.78) treated with abemaciclib had favourable OS outcomes compared to palbociclib. Overall, OS outcomes were comparable between agents, with no definitive superiority across most subgroups.

Comparisons between abemaciclib and ribociclib also showed largely non-significant differences. In the IPTW analyses, hazard ratios for OS were generally close to 1.0, with no general trend in statistical significance. It was noted that in patients with ECOG performance score of 0, abemaciclib was favored (HR 0.63, 95%CI: 0.47-0.85). Overall, OS outcomes appeared comparable between agents, with some subgroup-specific nuances.

Across subgroups, OS outcomes were largely similar among the three agents, with only a handful of significant hazard ratios. These findings are constrained by inconsistent sample sizes (12 to 4,458 across all reported HRs) and incomplete sample size reporting, which adds heterogeneity and limits the robustness of comparisons.

Additional subgroup details can be found in **Appendix G and Appendix H**.

Table S16. Full-text study definitions of rwPFS

Study name; Author	rwPFS Definition
NR; Buller, 2023	NR
NR; Cejuela, 2023	Time in months from the start of treatment with CDK4/6i to the cutoff date, disease progression, or death.
NR; Kahraman, 2023	Time from CDK4/6i treatment initiation to disease progression or death, whichever was earlier.
NR; Tang, 2023	Time from initiation of CDK4/6i therapy to radiological progression, clinical progression or death.
YOUNGBC-28; Chen, 2024	Time from treatment initiation to disease progression or death.
NR; Coutinho-Almeida, 2024	NR
CEPRA; Dajsakdipon, 2024	NR
NR; Gehrchen, 2024	Date of metastatic disease to progression or death in first line.
NR; Karhan, 2024	Time from starting CDK4/6is until disease progression or death from any cause, whichever first occurred.
NR; Oner, 2024	Duration from the commencement of treatment to disease progression, discontinuation of therapy due to any cause, or death.
NR; Skocilic, 2024	Time from the start of CDK4/6is to clinical or radiologic progression.
OPAL; Thill, 2024	Interval between start of first-line treatment and date of progression or death.
TOG study; Yildirim, 2024	Time from CDK4/6i initiation until disease progression or death from any cause.
NR; Yildirim, 2024	NR
NR; Guliyev, 2025	The time from the start of treatment to disease progression or death from any cause, whichever occurred first
NR; Inci, 2025	NR
PALMARES-2; NCT06805812 Provenzano, 2025	Time interval between the initiation of ET plus CDK4/6is and the detection of disease progression, as evaluated according to radiological (CT/PET scans), clinical (clinical tumor measurements and evolution of patient status) or biochemical criteria (CA15.3 measurements), or patient death, whichever occurred first.
P-VERIFY; NCT06495164 Rugo, 2025	Number of months from the start of CDK4/6i plus AI treatment to death from any cause or disease progression (based on clinical assessment, radiographic scan, or tissue biopsy), whichever occurred first.
CDK-PREDICT study; Tolosa, 2025	NR
NR; Yoshinami, 2025	Time from the start of the relevant treatment for a/mBC to death from any cause or disease progression (based on data collected from the medical records of each patient), whichever occurs earlier.

Abbreviations: a/mBC = advanced/metastatic breast cancer; AI = aromatase inhibitor; CA15.3 = cancer antigen 15.3; CDK4/6i = cyclin-dependent kinase 4/6 inhibitor; CT = computed tomography; ET = endocrine

therapy; NR = not reported; PET – positron emission tomography; rwPFS = real-world progression-free survival.

Forest Plots of OS and PFS Hazard Ratios

Figure S2. Forest plot of OS and PFS hazard ratios for overall first-line abemaciclib versus ribociclib

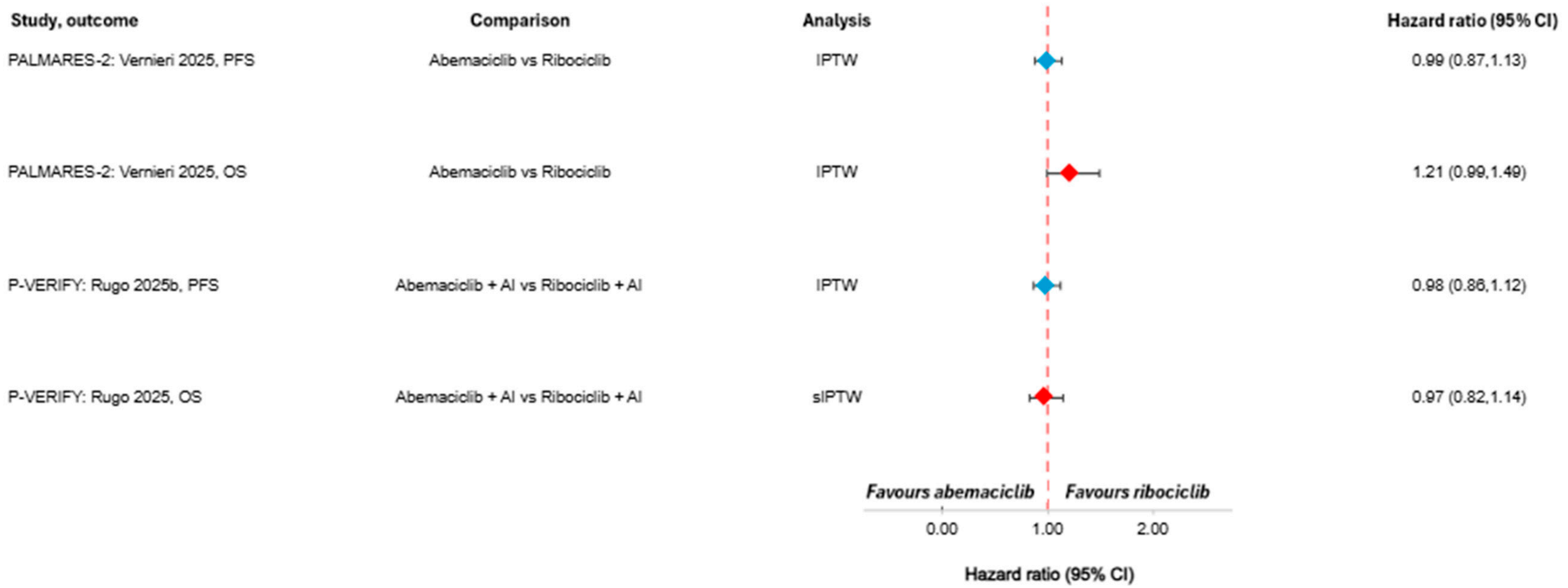

*Reported hazard ratios and associated confidence intervals were inverted to align with reporting style from other studies
